# Reported patterns of pregnancy termination from Demographic and Health Surveys

**DOI:** 10.1371/journal.pone.0221178

**Published:** 2019-08-19

**Authors:** David A. Sánchez-Páez, José Antonio Ortega

**Affiliations:** 1 Programa de doctorado en Economía, Departamento de Economía e Historia Económica, Universidad de Salamanca, Salamanca, Spain; 2 Departamento de Economía e Historia Económica and Instituto Multidisciplinar de Empresa (IME), Universidad de Salamanca, Salamanca, Spain; University of Cape Coast, GHANA

## Abstract

**Introduction:**

Demographic and Health Surveys, widely used for estimation of fertility and reproductive health indicators in developing countries, remain underutilized for the study of pregnancy termination. This is partly due to most surveys not reporting the type of pregnancy termination, whether spontaneous or induced. Reproductive calendar data makes it possible to examine termination patterns according to contraceptive use at the time of pregnancy. Contraceptive failure is expected to increase the likelihood of induced abortion helping in the interpretation of reported termination patterns.

**Materials and methods:**

We use individual-level calendar data regarding 623,966 pregnancies to analyze levels and differentials in reported patterns of pregnancy termination by age, union status, and contraceptive use in 107 DHS surveys from 50 countries. From the estimates of the probability of pregnancy termination, we compute derived reproductive health indicators providing an assessment of what is driving the differences by comparison to the few surveys reporting the type of pregnancy termination.

**Results:**

From our estimates, 10.9% of pregnancies do not end in live-birth and 63.7% of them are spontaneous terminations. Reported pregnancy termination is higher among women using contraceptives, consistent with expectations. Very low levels of reported PT in some countries, particularly in sub-Saharan Africa, suggests possible underreporting. Differential patterns emerging from cluster analysis and regional rates indicate high rates of pregnancy termination driven by induced abortion in countries from the Former Soviet Union and Asian countries with liberal laws. Most countries with restrictive abortion laws have low levels of reported termination. While the probabilities of pregnancy termination are higher at older ages, termination rates generally peak at younger ages due to higher conception rates.

**Discussion:**

This is the first large comparative study of the patterns of reported pregnancy termination in DHS surveys. While we have explored the extent to which differences arise from spontaneous terminations or induced abortion, more research is needed regarding the determinants of reported pregnancy termination.

## Introduction

Demographic analysis of fertility focuses on live births, but not all pregnancies are carried to term. A pregnancy ending before live-birth, regardless of the reason, is associated with a pregnancy termination (PT). PT includes both spontaneous terminations (ST) —miscarriage and stillbirth— and induced abortions (IA). The incidence of PT affects fertility levels since a sizable proportion of pregnancies, ranging between 4.9% and 52.0% in a comparative study of 20 countries, end in PT instead of a live birth [1]. In that study, high proportions of PT were only observed in countries with high levels of IA.

Much of what is known regarding fertility levels in developing countries is based on nationally representative demographic surveys. In particular, Demographic and Health Surveys (DHS) are, since 1985, a significant source of information regarding fertility and its proximate determinants like union formation, contraceptive use, and sterility. However, they are rarely used for the estimation of IA or ST [2–5]. There are several reasons for this. A first factor behind this limited use is the lack of information regarding the type of termination (IA versus ST) in most DHS surveys. This leads some sources to only use those few surveys reporting the type of termination [6]. A second one is a concern regarding the completeness of coverage and possible misclassification of outcomes. The only comparative survey of PT according to outcome based on retrospective survey data dates back to the World Fertility Survey [7]. It showed significant differences in the reported incidence of ST among countries and according to sociodemographic variables and generally low reported rates of IA. A recent DHS technical report has analyzed comparative levels of PT to check the consistency of reporting according to time since the interview [8]. This research finds signs of underreporting of PT when going back in time, particularly in some countries such as in sub-Saharan Africa. Probably due to these concerns and, in particular, low levels of reported IA in countries where abortion is illegal or heavily restricted, international monitoring efforts that use DHS and related surveys in monitoring reproductive health outcomes, prefer to use regional and subregional estimates derived from other indirect sources to impute the incidence of IA at the country-level in those countries [3, 6, 9]. In the period 2010-2014, subregional estimates of IA ranged between 12% and 39% of pregnancies [3].

While we share the concern regarding the completeness of coverage, we feel that data on PT has been dismissed as useless before studying it and we pretend to fill this data gap by analyzing the available information on DHS surveys on PT in order to identify patterns in reported PT. In particular, we make use of the information contained in DHS surveys on contraceptive use at the time of pregnancy. Since pregnancies arising from contraceptive failure are unintended, they are more likely to end in an IA [1, 10–12]. We use the few surveys that include details on the type of PT to highlight that differences across surveys in PT are, for the most part, connected to different levels of IA, but also that there remain important differences in levels of reported ST in countries with low reported IA. Previous studies on the incidence of IA highlight, among others, the effect of age and union status [13–17]. The likelihood of IA increases with age to the extent that it is used to limit family size. Pregnancies occurring outside of unions, on the other hand, might be more likely to be aborted irrespective of family size. Age is also a relevant predictor of the medical risk of ST with a U-shaped age-gradient [18–20]. For these reasons, we identify patterns of pregnancy termination according to age, union-status, and contraceptive use at the time of pregnancy.

Regarding the interpretation of differences in reported PT, little is known regarding the drivers of reported ST. It is recognized that cultural factors are important both as drivers of self-perception of ST and recall patterns [7, 21]. Despite a relevant share of pregnancies ending in miscarriage, a cultural norm of silence surrounds them [22, 23]. This could be related to grief after facing a loss and possible stigma [24, 25]. Moreover, memory could be affected after traumatic experiences so that events related to grief are forgotten [26, 27]. On the other hand, while it might be true that some part of differences in reporting might be due to forgetting in some cultural settings, and that for these reasons we should not expect annual time series derived from DHS to be reliable [8], that is only a small part of the variability in reported termination rates. Reported levels of ST tend to be relatively stable over time [21] and reported differentials according to socio-demographic characteristics tend to agree with medical knowledge [7, 21]. What remains poorly understood is the connection between reported levels, biological determinants of ST, cultural elements behind self-awareness and recall and the functioning of public health systems. In order to advance in this direction, it is necessary first to put the estimates on the table. Prospective cohort studies of ST and IA are often seen as an alternative, more objective way to measure PT. While large scale prospective cohort studies from developing countries are rare, detected levels of ST and IA in a recent comparative study are much lower than those reported in DHS surveys [28]. In the case of IA, intentional underreporting is even more likely than for ST [2]. In particular, we can fear that women are more reluctant to report an IA in a context where it is illegal. We will, therefore, look at differences in reported PT according to the legal status of IA [9]. However, women, particularly those from more deprived settings, might not be aware of changes in the law [29], and, in any case, we cannot be sure to what extent a relationship between reported PT and abortion-legality status is due to increased levels of underreporting or to a lower probability of IA. Problems in understanding concepts such as termination or induced abortion can also be at stake [30].

Regarding the implications of the study, universal access to Sexual and Reproductive Health by 2030 is part of the Sustainable Development Goals [31]. Also, the Family Planning 2020 global partnership includes as goals, among others, increasing contraceptive prevalence, reducing unintended pregnancies, and averting unsafe abortions [32]. Differences in PT according to contraceptive use highlight the consequences of contraceptive failure. The use of more effective methods of family planning can prevent unintended pregnancies and avoid IA. In this respect, it is important to differentiate between the conditional probability of pregnancy termination that will be of relevance in a medical context, and the underlying termination rates that have public health implications. While we find that the conditional probabilities increase with age, termination rates are generally higher for women at peak reproductive ages given their higher risk of conception [13]. Combining our estimates of the Total Termination Rate with fertility estimates, we can detect the relationship between modern contraceptive prevalence and the Total Pregnancy Rate.

Our research is also relevant regarding fertility estimation based on the proximate determinants framework [33, 34] at the core of aggregate models of reproductive health such as the Spectrum model [35]. This model is based on independence among proximate determinants such as union formation, contraceptive use, and abortion. In contrast, we explicitly measure differences in PT according to union status and contraceptive use.

## Materials and methods

### Data

DHS surveys are a rich source of information, especially regarding fertility and family planning. For most countries, DHS surveys collect information using monthly calendar data going back up to 72 months [36]. Our goal is to analyze the patterns of pregnancy termination according to contraceptive use at the time of pregnancy and according to age and union status. For this purpose we use three different calendars: The contraceptive use and reproductive history calendar (cal1), registers pregnancies, pregnancy outcomes, and contraceptive methods used. It identifies when a pregnancy begins and whether it ends in a live-birth or not. The second calendar (cal2) identifies the reasons for discontinuing or changing the contraceptive method used. Among others, cal2 indicates when a woman “became pregnant while using” so that contraceptive use at the time of pregnancy can be perfectly identified. The third calendar (cal3) records marital status. From cal3 we know if women were in-union or not-in-union at the time of pregnancy.

Unfortunately, not every survey includes the three calendars we need. In surveys where cal2 is absent, we assume a pregnancy occurred while using when a contraceptive method was being used in the month preceding the pregnancy. For surveys not including cal3, we impute union status based on the date of the first union and the duration of that union. On the other hand, some DHS surveys only represent women in union. We use all DHS surveys that include all women irrespective of union status and reporting at least cal1. S1 Table details the surveys included and the calendars used. After screening for these conditions, our database consists of 107 DHS surveys from 50 low- and middle-income countries, collected between 1990 and 2017, and includes individual-level information for 1,468,524 women aged 15-49 at the time of the interview (S2 Table). These surveys belong to Africa, Central and West Asia & Europe, Latin America and South and Southeast Asia.

We analyze all pregnancies that started in the 45 to 9 months preceding the interview. Pregnancies in the eight months preceding the interview are excluded to avoid right censoring. In this way, except for a small number of premature births, we capture all births occurring in the 3-years before the interview. That is the same framework used for fertility estimation in DHS. This allows us to move from probabilities of termination to age-specific termination rates. To ensure that the age-groups are comparable, we assign age according to imputed age at birth. This is equal to age at birth for pregnancies carried to term, and age at pregnancy plus nine months for the rest of pregnancies. We use standard five-year age-groups except for the 40-49 age-group due to the small number of pregnancies at age 40 and above. A few pregnancies with an imputed age at birth of less than 15 are excluded in line with DHS fertility estimation. Our sample includes 623,966 pregnancies, of which 555,908 are live-births (outcome B) and 68,058 pregnancy terminations (outcome PT) (S2 Table). Most DHS surveys do not collect the type of PT. In our case, only 16 DHS surveys identifying the type of PT meet our requirements, mostly from countries where abortion is legal. We use these surveys to assess specific patterns of IA and ST according to contraceptive use, and, most importantly, to shed light on the likely distribution of PT in the surveys not reporting the type of termination.

Pregnancies are further classified according to union status and contraceptive use at the time of pregnancy. According to DHS definitions, married women and those in consensual unions are grouped as in-union. Women that are never married, divorced, widowed, or separated are grouped as not-in-union. Regarding contraceptive use at pregnancy, users of any method at the time of pregnancy are classified as using. The reason is that, irrespective of the efficacy of the contraceptive method used, the use of any method hints at a desire to avoid pregnancy.

Age-specific termination rates (ASTR) and general termination rates (GTR) for all women are derived from the age-specific probabilities of PT and age-specific fertility rates (ASFR) computed by the DHS program for the three years before the survey. We obtain ASFR, general fertility rates (GFR) and contraceptive prevalence rates from the DHS API webpage using the R package rdhs [37].

### Methods

#### Probability of pregnancy termination

We estimate separate conditional probabilities of PT (*T*) for each combination of age-group, union status, and contraceptive use at the time of pregnancy at the survey level. DHS surveys are complex surveys representative at the national level with a stratified two-stage cluster design. Given unequal probabilities of selection we use women weights (*w*_*i*_) so that the conditional probability is computed as the ratio of the weighted number of pregnancies ending in termination to the total weighted number of pregnancies irrespective of outcome (*p*):

$$\begin{array}{c} T_{s,a,m,u}=\frac{\sum w_{i}\cdot {\left(p=PT\right)}_{s,a,m,u}}{\sum w_{i}\cdot {\left(p=PT\right)}_{s,a,m,u}+\sum w_{i}\cdot {\left(p=B\right)}_{s,a,m,u}} \end{array}$$ (1)

The subscripts *a*, *m*, and *u* refer to age-group, union-status, and contraceptive use at the time of pregnancy, respectively. *s* identifies the particular subpopulation analyzed. It can be a specific survey, a pooled regional sample or the total pooled sample. For surveys reporting the type of pregnancy termination, we follow the same approach to derive the conditional probabilities for each termination type, ST and IA. All calculations are carried out in R [38] using tidyverse packages [39] and purposely written functions for managing DHS reproductive calendar data.

Approximate binomial confidence intervals are derived from the unweighted number of cases using the Wilson method [40]. For this purpose, we use the binconf function from R package Hmisc [41].

#### Clustering

In order to identify common patterns of pregnancy termination at the survey level according to age-group, union-status, and contraceptive use at pregnancy, we use cluster analysis. Unfortunately, in many surveys sample size is too small for accurate estimation of *T*, especially among older women not-in-union, or among contraceptive users in countries with low contraceptive prevalence. With the view to minimize the problem, we have regrouped pregnancies to women not-in-union in only two age-groups before performing the cluster analysis: 15-24 and 25-49. There are still some combinations where the probability is based on less than 10 unweighted pregnancies. This happens for 12.1% of the categories. Given the considerable uncertainty involved in those estimates we have preferred to set them as missing data in combination with the use of a variant of the *k*-means cluster analysis algorithm, *k*-POD, that allows for missing data while simultaneously imputing the missing data to the cluster average [42]. *k*-POD uses a majorization-minimization algorithm to identify a clustering according to the observed data and retains the information without assuming any distribution over the missingness patterns. We have reprogrammed the algorithm in R package kpodclustr [43] to use multiple initial values in order to avoid issues of lack of convergence.

Regarding the choice of the number of clusters, we use the gap statistic method since it usually outperforms other methods proposed in the literature [44]. The optimal number of clusters is 4. The interpretation of the clusters is based on the cluster averages for each of the conditional probabilities, and Principal Component Analysis (PCA) that extracts the linear combinations of variables representing the largest possible variability present in the data [45]. In our case, the first two principal components represent 84.2% of the variance. The computations are carried out using R packages factoextra [46] and FactoMineR [47].

#### Termination and pregnancy rates

Given our choice of the time-window and our use of imputed age-at-birth instead of age-at-pregnancy, *T* can be combined with reported ASFRs for the 3-years before the survey to derive reproductive health indicators like *ASTR*, *GTR*, and the total termination rate (*TTR*). While *T* indicates what happens once the pregnancy takes place, the rates provide an estimate of the likelihood of a woman experiencing a termination in a given year. *TTR* can be interpreted as the expected number of terminations throughout the reproductive years in a synthetic cohort experiencing current *ASTRs*.

*ASTR* for a particular sub-group *i* can be defined as

$$\begin{array}{c} {\text{ASTR}}_{a}=\frac{PT_{a}}{N_{a}} \end{array}$$ (2)

where *PT*_*a*_ represents the number of terminated pregnancies in the subgroup of women of age *a*, and *N*_*a*_ is the number of woman-years of exposure. *ASFR*_*a*_ is defined equivalently as $\frac{B_{a}}{N_{a}}$ where *B*_*a*_ represents the number of births. Since *T*_*a*_ represents the probability of pregnancy termination, 1 − *T*_*a*_ represents the probability of a pregnancy ending in live-birth. Thus, we can estimate *ASTR*_*a*_ as:

$$\begin{array}{c} {\text{ASTR}}_{a}=\frac{PT_{a}}{B_{a}}\cdot \frac{B_{a}}{N_{a}}=\frac{T_{a}}{1-T_{a}}\cdot {\text{ASFR}}_{a} \end{array}$$ (3)

A similar calculation can be carried out for the *GTR* as a function of the *GFR*

$$\begin{array}{c} \text{GTR}=\frac{T}{1-T}\cdot \text{GFR} \end{array}$$ (4)

In this case, *T* is the probability of pregnancy termination based on all pregnancies.

*TTR* is obtained by aggregation of the respective *ASTR*s. In the case of 5-year age-groups, it is given by:

$$\begin{array}{c} \text{TTR}=\sum_{a}5\cdot {\text{ASTR}}_{a} \end{array}$$ (5)

This is a parallel definition to that of the Total Fertility Rate (*TFR*). An estimate of the number of lifetime pregnancies expected over a woman’s reproductive ages, the Total Pregnancy Rate (*TPR*), can be computed as the sum of *TFR* and *TTR*:

$$\begin{array}{c} \text{TPR}=\text{TFR}+\text{TTR} \end{array}$$ (6)

Note that *TPR* should conceptually include pregnancies ending in ST as in our case. Other investigators have used an estimate of *TPR* only including pregnancies resulting in birth or IA [5].

#### Tentative separation of terminations as induced or spontaneous

While DHS surveys do not provide information on the type of PT for most surveys, it is possible to use the information contained in those few surveys that report it for a tentative separation of terminations in induced and spontaneous. Based on the 16 DHS surveys with information on the type of outcome, we have estimated logistic regression models for the probability of IA conditional on termination. The simple idea is that higher values of *T* will be associated with a higher proportion of IA among PT. Since IA is expected to be more frequent among women who were using contraceptives at the time of pregnancy, we use the conditional probabilities according to contraceptive use providing a total of 32 data points. We estimate two models (Table 1).

**Table 1:** Model estimates of the probability of induced abortion from the probability of pregnancy termination (*T*).

	Model 1	Model 2
Intercept	−1.635*	−1.632**^+^
	(0.836)	(0.826)
	[−3.274; 0.004]	[−3.252; −0.013]
*T*	7.582**^+^	6.733**^+^
	(3.220)	(2.796)
	[1.271; 13.893]	[1.253; 12.212]
*use* = 1	−0.584	
	(1.007)	
	[−2.558; 1.390]	
AIC	29.716	28.028
BIC	34.113	30.959
Log Likelihood	-11.858	-12.014
Num. obs.	32	32

***p < 0.01,

**p < 0.05,

*p < 0.1,

^+^ 0 outside the confidence interval

The first model includes independent variables *T* and contraceptive use. The second model only *T*. Since contraceptive use is not statistically significant in the first model and its AIC value is higher, we keep the second model. We, then, compute a tentative probability of IA by multiplying the predicted values of the model by *T*. ST is the difference between *T* and the probability of IA. This simple approach provides an educated guess at what the relative proportions of IA and ST are in those surveys reporting all terminations together. While a simple approximation, it is complex enough to capture that the probabilities of ST decline when IA is very high due to the competing nature of both risks since women undergoing an IA are no longer at risk of ST [48].

## Results

### Patterns of pregnancy termination

**Fig 1:**
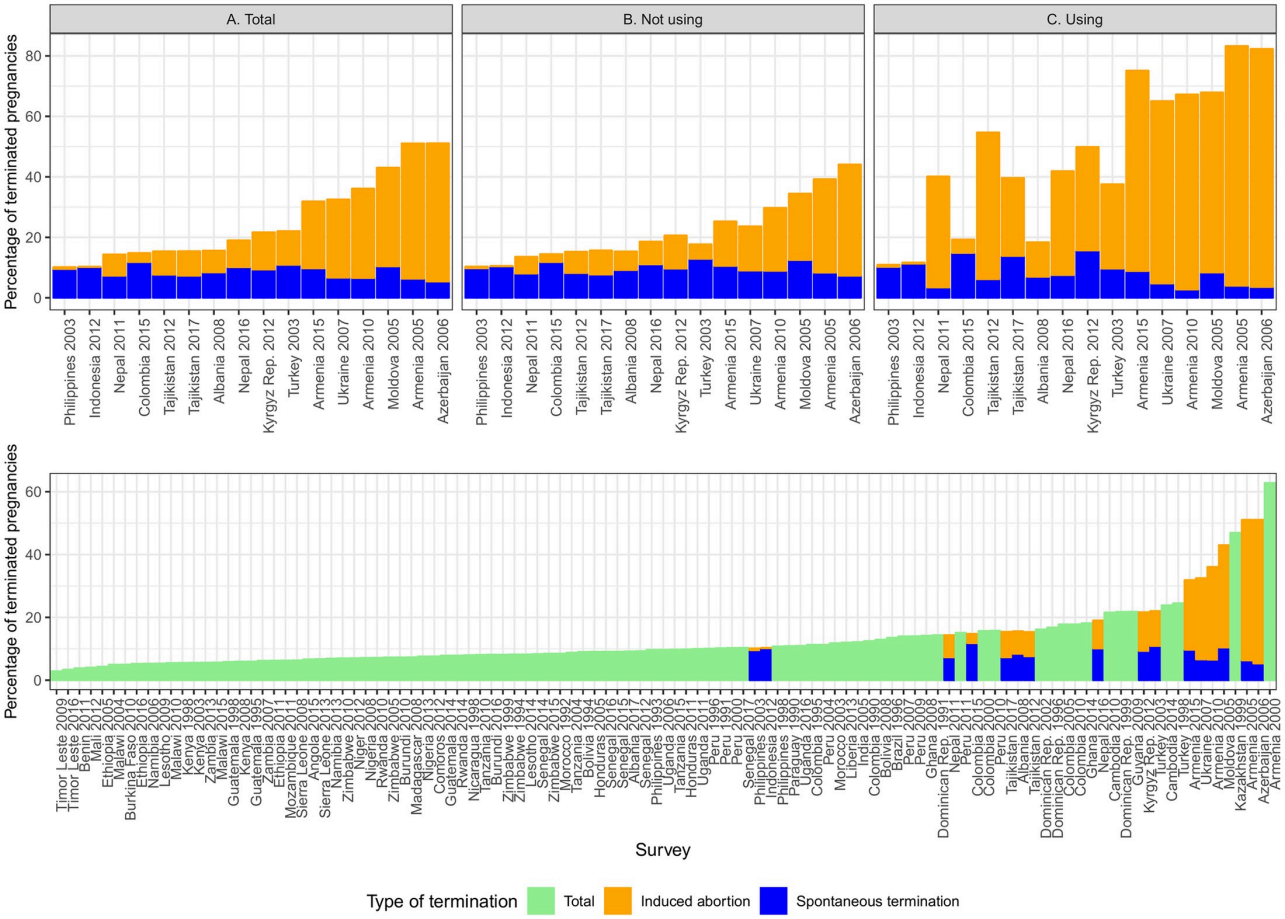
Probability of pregnancy termination by survey.

Levels of *T* at the survey level vary significantly between surveys and according to demographic characteristics (S3 Table). The lower panel of Fig 1 displays the overall percentage of terminated pregnancies, *T*, for the 107 surveys. For those surveys that report the type of outcome, the bars display the respective contribution of IA and ST to all terminations. A first pattern emerges: High values of *T* are connected with a high prevalence of IA, with ST levels not increasing or even decreasing in countries with high proportions of terminated pregnancies. We also see that most countries reporting the type of PT are high abortion countries except for Indonesia 2012 and Philippines 2003. However, most of the surveys not reporting the type of outcome have low proportions of PT suggesting that in those countries most reported terminations are spontaneous.

The upper panel of Fig 1 introduces the differences in the type of outcome according to contraceptive use at the time of pregnancy for those 16 surveys reporting the type of PT. Graph A contains the same information of the lower panel whereas graphs B and C refer to not-users and users of contraception respectively, the latter experiencing contraceptive failure. We can see that, consistent with our expectations, the probabilities of termination are much higher for women that were using contraceptives, indicating that they were not willing to get pregnant. The reason behind is a higher level of IA resulting in countries where most pregnancies occurring while using do not end in a live-birth. Indeed, those countries with an extremely high prevalence of IA have, if something, lower levels of ST probably due to the competing nature of the risks. Whereas women using have the highest rates of IA, and therefore T, countries with a high incidence of abortion among users tend also to have higher abortion rates among not users.

**Fig 2:**
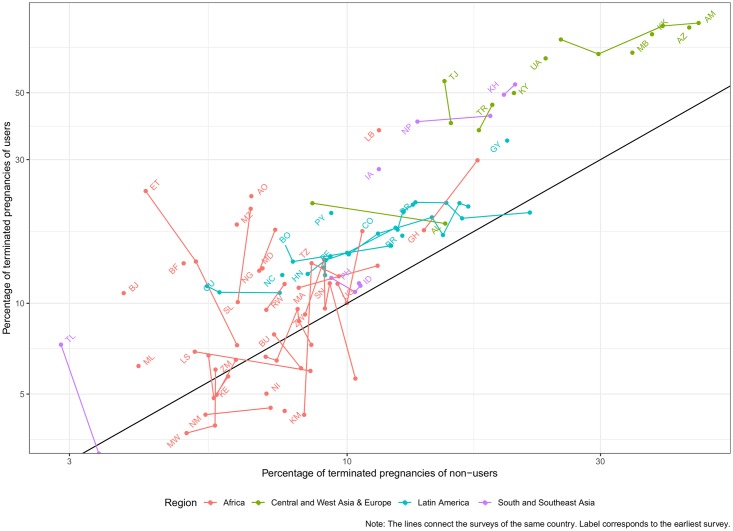
Probability of pregnancy termination by contraceptive use at pregnancy.

Fig 2 shows the relation between *T* of users and non-users in all surveys using a logarithmic scale. Almost all surveys are above the black diagonal (x = y). This means that women experiencing contraceptive failure are more likely to report terminations than women not using contraceptives. Given the patterns found in Fig 1 for surveys with information on the type of outcome, the most likely explanation is that contraceptive users are more likely to recur to IA. While the probability of termination is higher among users than not users, a positive association is observed in consonance with the results for the countries reporting the type of PT. This means that countries with relatively high levels of PT among users also tend to have high T for non-users. Regional differences can also be inspected by looking at color. Countries in Central and West Asia & Europe tend to have the highest levels of T both for users and non-users. Latin American countries tend to have medium levels of termination for both groups. All African countries have relatively low levels of T with relatively high variance in the differences according to contraceptive use. South and Southeast Asia is very heterogeneous with countries like Cambodia and Nepal having high reported termination rates, whereas Timor Leste reports the lowest levels for both users and not-users. Lines connect surveys of the same country and labels are placed in the point of the earliest survey. Ascending lines tend to predominate indicating that termination rates move together for users and non-users, but there are exceptions, mostly in countries with low levels of T, like in Africa or Asia. Regarding trends over time, there are countries with increasing termination rates like Ghana or Nepal with others like Armenia experiencing declining rates.

**Fig 3:**
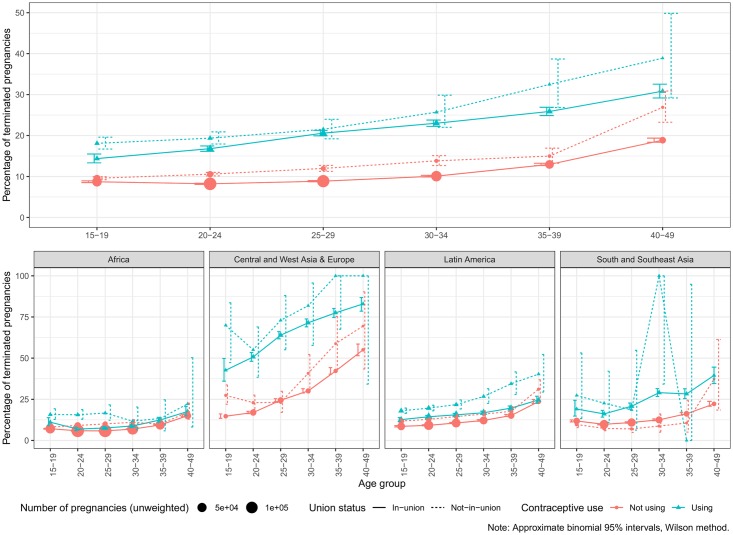
Probability of pregnancy termination according to age, union status, and contraceptive use at pregnancy.

Overall patterns of PT by age and union status are shown in the upper panel of Fig 3. We can see that contraceptive users are more likely to experience terminations for all combinations of age and union status confirming that contraceptive failure points to a more likely use of IA. The overall percentages of *T* are 20.9% and 9.8%, respectively. Regarding the patterns according to age, in the case of contraceptive users, the likelihood of termination increases monotonically with age irrespective of union status. This is consistent with the use of IA at older ages to limit family size. In the case of non-users in-union, the largest group, *T* is minimal for the age-group 20-24 increasing monotonically at older ages. This is consistent with medical evidence on a minimum risk of ST at peak fertility ages. Irrespective of union status, the minimum risk of PT is reached at ages 20-24 (9.3% of terminated pregnancies) reaching a maximum of 20.4% at ages 40-49. Regarding union status, and for all combinations of use and age, women not in union are at a slightly higher risk of termination. On average, *T* is 10.8% for in-union women and 12% for those not-in-union.

Results by region tend to share the same demographic patterns. In general terms, *T* increases with age beyond the 20-24 age-group, and it is higher for not-in-union women and women experiencing contraceptive failure (lower panel of Fig 3). Nevertheless, there are sharp regional differences in the likelihood of PT and the relative importance of these variables. Africa has the lowest average *T* in our sample, 7.4%. Also, it shows the least differences among contraceptive users and not-users suggesting very low reported IA, with one exception: Women 15-29 not-in-union using contraception report somewhat higher termination rates suggesting some use of IA to avoid births outside of an union. In contrast to Africa, Central and West Asia & Europe has the highest estimates of *T* in our sample, 30.7%, and the highest differences according to contraceptive use: 64.9% of terminated pregnancies for users compared to 23.9% for not-users. This, again, suggests a high incidence of IA. Latin America lies in middle-ground compared to the previous two regions with an average *T* of 12.7%. This region presents an increasing trend by age from 10.5% at ages 15-19 to 24.5% at 45-49. Also, there are differences in *T* by union status and contraceptive use, 12.2% and 15.1% for in-union and not-in-union women, and 17.1% and 11.5% for users and not-users. In the case of South and Southeast Asia, we notice large confidence intervals for women not-in-union due to a combination of almost universal marriage and low fertility outside of marriage. The average *T* is similar to Latin America with an average *T* of 12.4%. We find a higher probability of PT as women ages, going from 10% at ages 20-24 to 24.2% at 40-49. However, the difference by union status is unclear due to the scarcity of cases for not-in-union women. According to contraceptive use at pregnancy, *T* is 23.8% and 11.6% for users and not-users, respectively. Detailed estimates by survey are in S4 Table.

**Fig 4:**
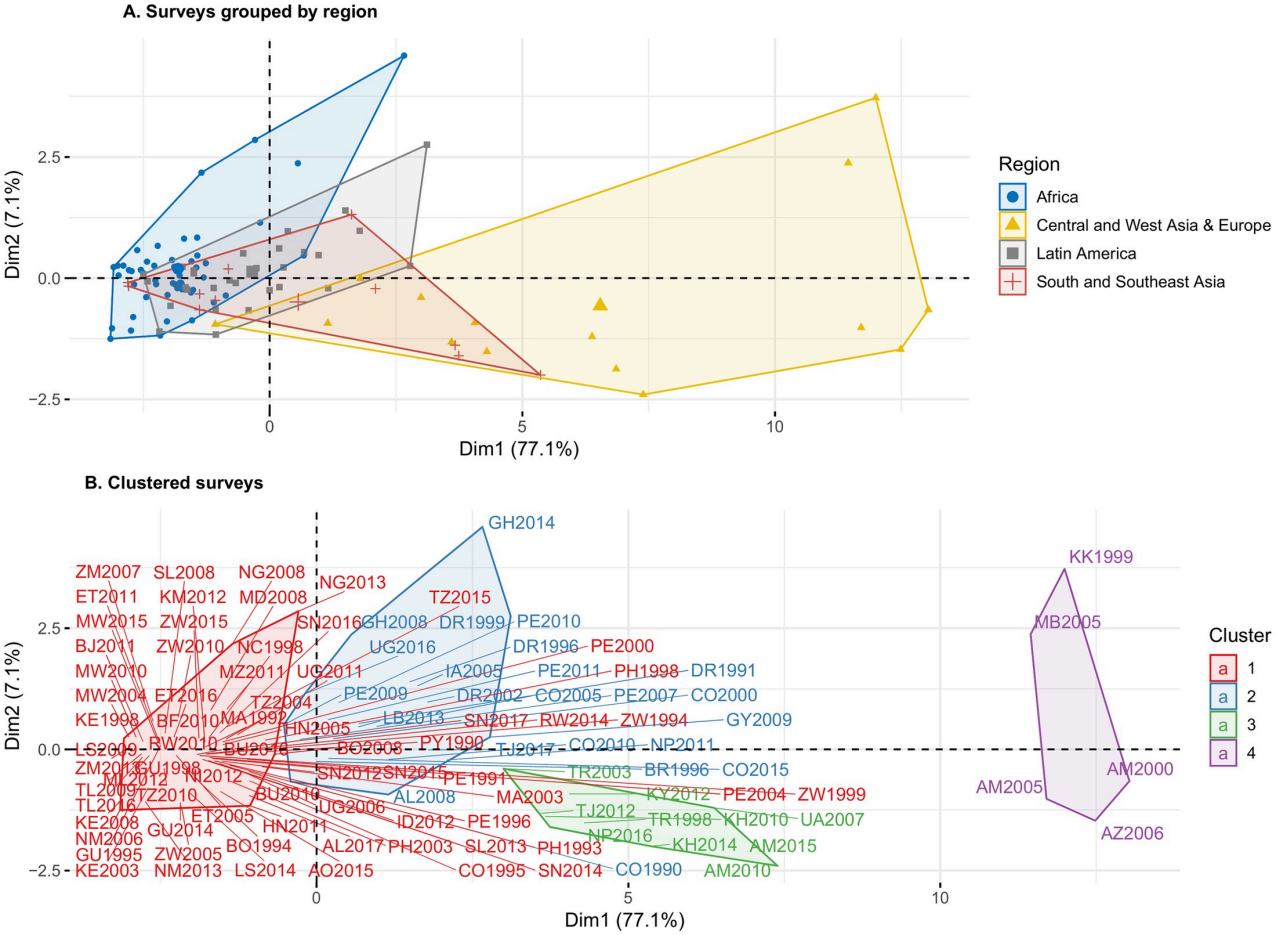
Principal components analysis by survey.

We identified earlier that some regions, and in particular Africa and South and Southeast Asia, are heterogeneous in terms of the risk of PT and the relative differences according to contraceptive use. Cluster analysis can help in characterizing more homogeneous groups. Given the low number of pregnancies in some categories of age and union-status at the country level, and as described in the methods section, we group women not-in-union in two large age-groups: 15-24 and 25-49. For the cluster analysis, each survey is characterized by 16 conditional probabilities: 8 for contraceptive users and 8 for non-users, for 6 age-groups in the case of women in-union and 2 age-groups for women not-in-union (see S5 Table for detailed estimates by survey). Four clusters emerge that have been labeled 1 to 4 in increasing order of T. These four clusters also have specific differentials according to age-group, union status, and contraceptive use at pregnancy. Such differential patterns are highlighted in the PCA. Fig 4 displays the surveys plotted according to the two first PCA dimensions. Principal component 1, capturing 77.1% of the variance, gives positive weight to all conditional probabilities providing a summary measure of terminations levels. Principal component 2 highlights differential patterns according to age, contraceptive use and union status, in particular, whether women not-in-union using contraceptives have higher *T* and the respective ages at which the risk of termination starts to increase (S1 Fig displays the analysis by variable).

**Fig 5:**
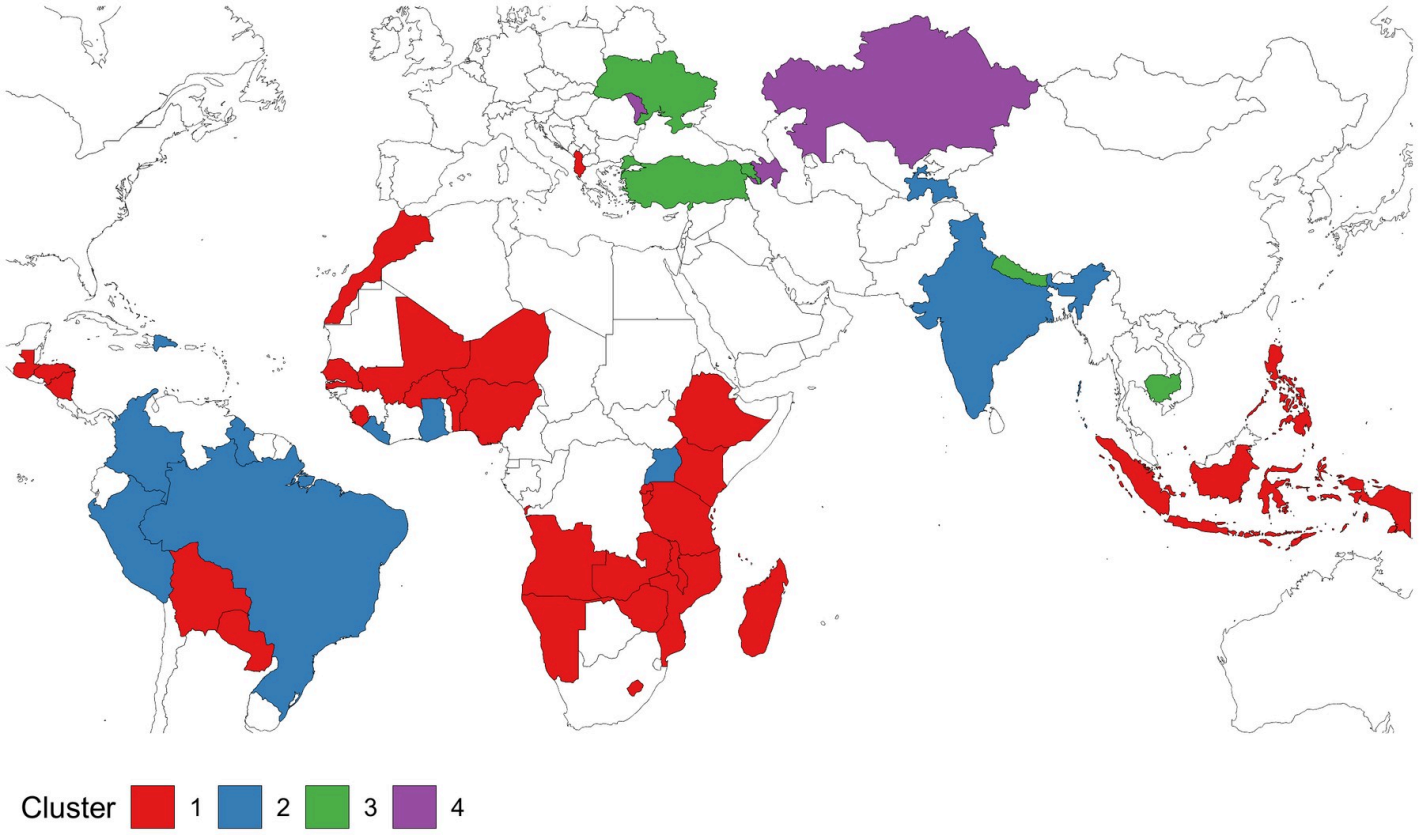
Countries by cluster in the latest DHS survey.

**Fig 6:**
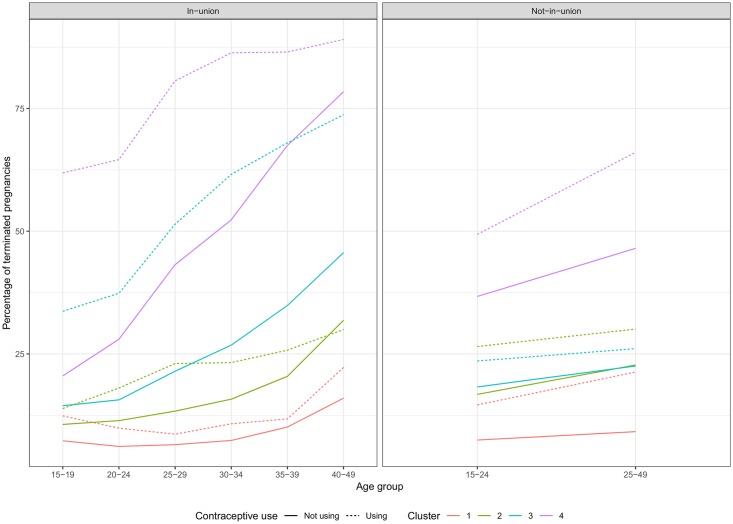
Cluster means by age, union status, and contraceptive use.

Graph A of Fig 4 shows surveys according to region whereas in graph B they are grouped according to cluster. Clusters are much more homogeneous than the regions, that overlap to a certain extent. This confirms that relatively homogeneous groups of countries can be found that are ranked according to the overall level of termination as suggested by dimension 1, but that also differ qualitatively according to dimension 2, as is the case of cluster 3. To better interpret the clusters, Fig 5 displays a map identifying the cluster to which the country belongs in the latest survey. Also, Fig 6 displays the cluster means for the different combinations of age-groups, union status, and contraceptive use. We notice how in all cases higher clusters have higher conditional probabilities of PT, but they differ in the relative differences from cluster to cluster. Cluster 1, red color, shows the lowest values of *T* with small differences according to union status. It is composed mainly of sub-Saharan Africa and insular Southeast Asia, but it also includes Central America, Bolivia, Paraguay, and Albania 2017. These would be countries reporting very few IA and very low levels of ST as well. In this cluster, reported pregnancies do not increase monotonically with age for women in-union. The minimum is observed at age 20-24 for not-users and 25-29 for contraceptive users. The only group that might be reporting some IA are contraceptive users not-in-union. Cluster 2, blue color, includes the rest of Latin American countries, South Asia, and some countries in sub-Saharan Africa (Ghana, Liberia, and Uganda 2016) with higher probabilities of termination than cluster 1. Minimum termination probabilities are observed in the youngest age group. Although termination rates are much lower than in cluster 3, particularly for in-union women using contraception, the differences disappear in the case of women not-in-union. Cluster 3, green color, includes some surveys from Europe and Asia characterized by high termination rates for women in-union with a large differential according to contraceptive use, and low probabilities of termination for women not-in-union. It includes Kyrgyzstan, Tajikistan, Turkey, Ukraine, Cambodia, Nepal 2016, and the latest Armenian surveys. Finally, cluster 4, purple color, includes surveys having high levels of *T* and large differentials according to age and contraceptive use. It includes countries in the Former-Soviet Union with a traditionally high incidence of IA like earlier Armenia, Azerbaijan, Kazakhstan, and Moldova. Both cluster 3 and 4 share high differentials in T according to age for women in-union suggesting the use of IA to limit family size.

It is interesting to document the few countries that change cluster over time since these tend to be associated with profound changes. Three countries are moving over time to a cluster with lower *T*: Armenia, from 4 to 3; Tajikistan, from 3 to 2; and Albania from 2 to 1. In contrast, there are also three countries moving upwards: Uganda from 1 to 2 in 2016, Peru from 1 to 2 in 2007, and Nepal from 2 to 3 in 2016. Colombia belongs in all six surveys to cluster 2 except for a temporary decline to cluster 1 in 1995.

**Fig 7:**
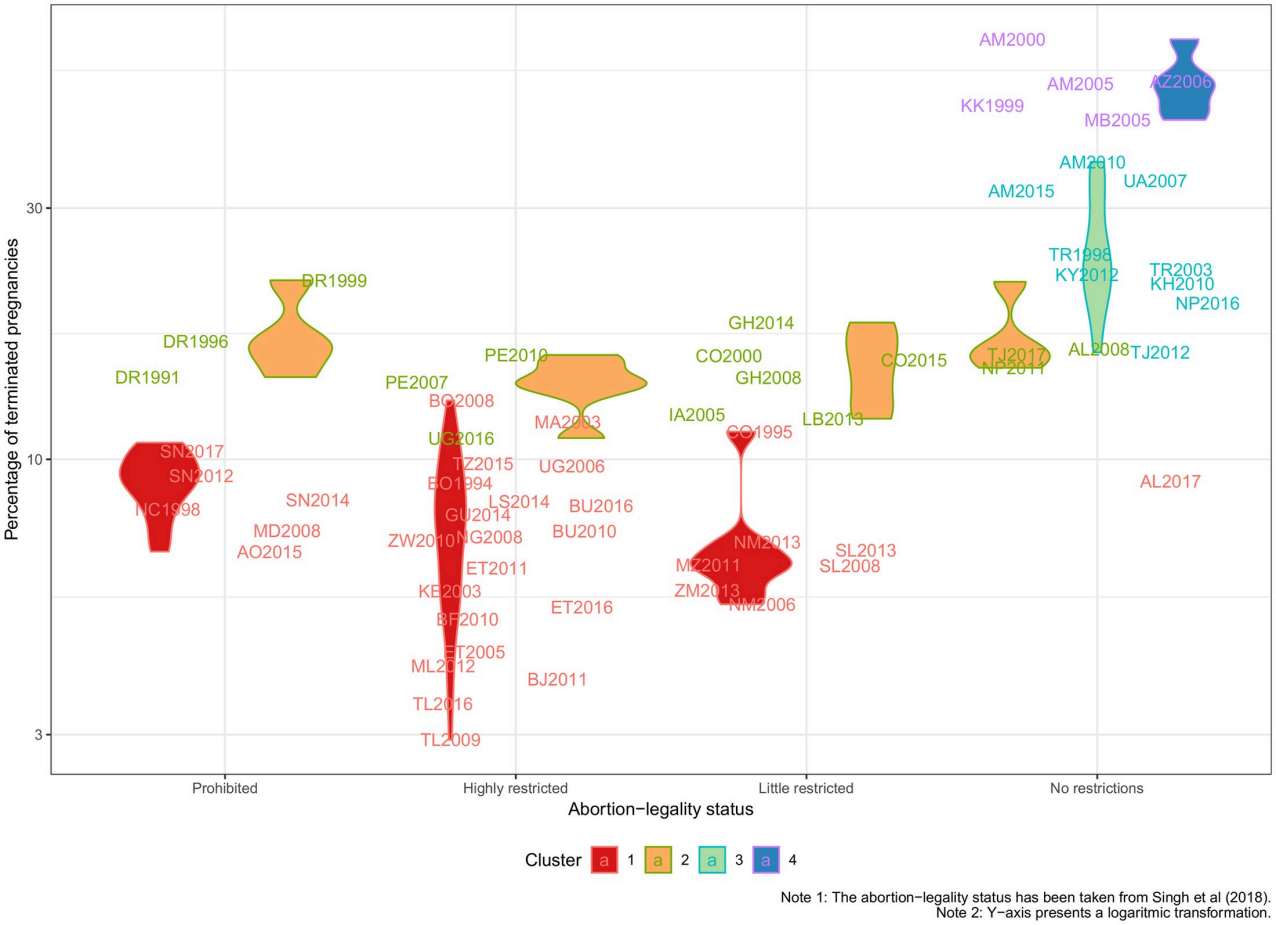
Probability of pregnancy termination by cluster and abortion-legality status.

Regarding possible explanations for the patterns found, we assess differences according to the legal status of abortion. Fig 7 displays violin plots of overall probabilities of termination in log-scale according to the cluster and how restrictive was the abortion law at the time of the survey. We see that all surveys in contexts of restrictive laws belong to clusters 1 and 2 of low termination. This suggests that in all countries with restrictive laws there are low reported levels of IA. As a result, differences in levels of reported ST must be behind the proportionally large differences in *T*, many of them too low even as estimates of ST only. While even in these countries with low reported terminations the magnitude and direction of differentials seem consistent, we cannot be sure based only on this evidence whether restrictive laws lead to low IA levels, or to underreporting of IA, due to concerns regarding legal implications. On the other hand, countries with less restrictive abortion laws are very heterogeneous, including countries belonging to all 4 clusters: Albania and Tajikistan are countries where abortion is legal but reporting low levels of termination. This suggests that a more liberal law does not necessarily mean high levels of IA. While underreporting might also be present here, there seems to be less rationale for the intentional omission of IA. At the other end of the spectrum, all the countries with a high incidence of termination driven by IA in clusters 3 and 4 are characterized by liberal abortion laws. Note that reported probabilities of termination can be extremely high, particularly for older women in-union using contraception.

There are also some countries with surveys that differ according to whether the type of PT is reported or not. It is the case of the Philippines, Colombia, Albania, Armenia, and Turkey. There does not seem to be systematic differences in reporting according to this dimension. In the Philippines, Colombia, and Turkey reported *T* are very similar in both cases indicating that this dimension does not drive the differences. In Albania, *T* is lower in the later survey not reporting the type of PT, but this is consistent with external evidence on the declining incidence of IA [49]. In the case of Armenia, the lower rates of *T* in later surveys including information on the type of outcome are internally consistent in pointing to declining abortion rates, although qualitative evidence points that there might be underreporting in later surveys connected with the growing importance of self-administered medication abortion [50].

**Fig 8:**
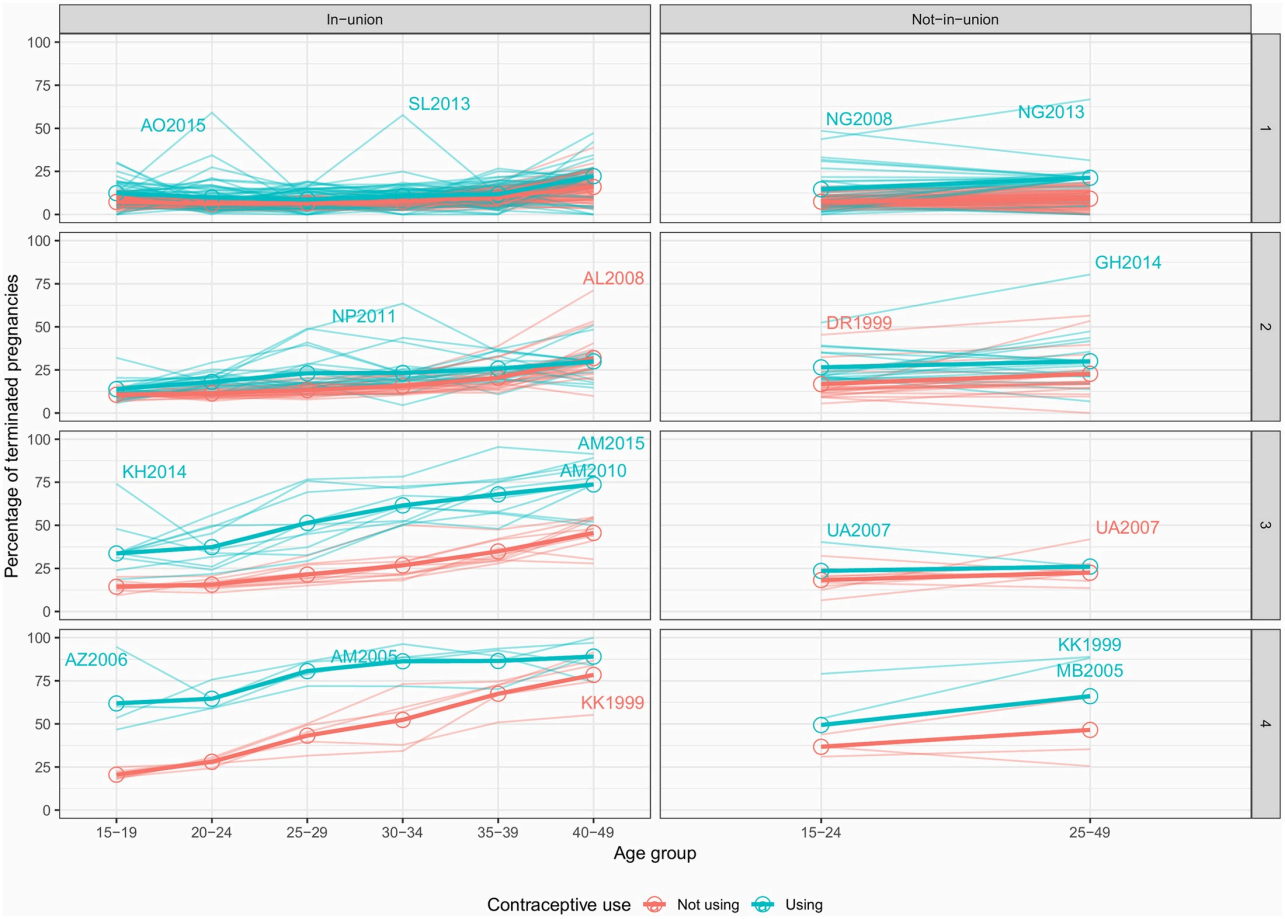
Probabilities of pregnancy termination by cluster and union status according to age and contraceptive use.

The survey-level variability at the cluster level can be appreciated in Fig 8, and it is reported in S4 and S5 Tables. Although each cluster includes only similar surveys, there are some outliers for a given age-group and union status. In particular, there are instances of countries with low overall levels of *T* in clusters 1 and 2 but having very large probabilities of IA for women not-in-union like Nigeria, Ghana, or the Dominican Republic. Albania belongs to the low termination clusters but shows relatively high termination rates for women in-union at ages 40-49. In clusters 1 and 2, the more considerable variability of probabilities for not-contraceptive users has to do with smaller numbers, therefore, showing more erratic patterns.

### Termination rates and tentative separation of terminations

**Fig 9:**
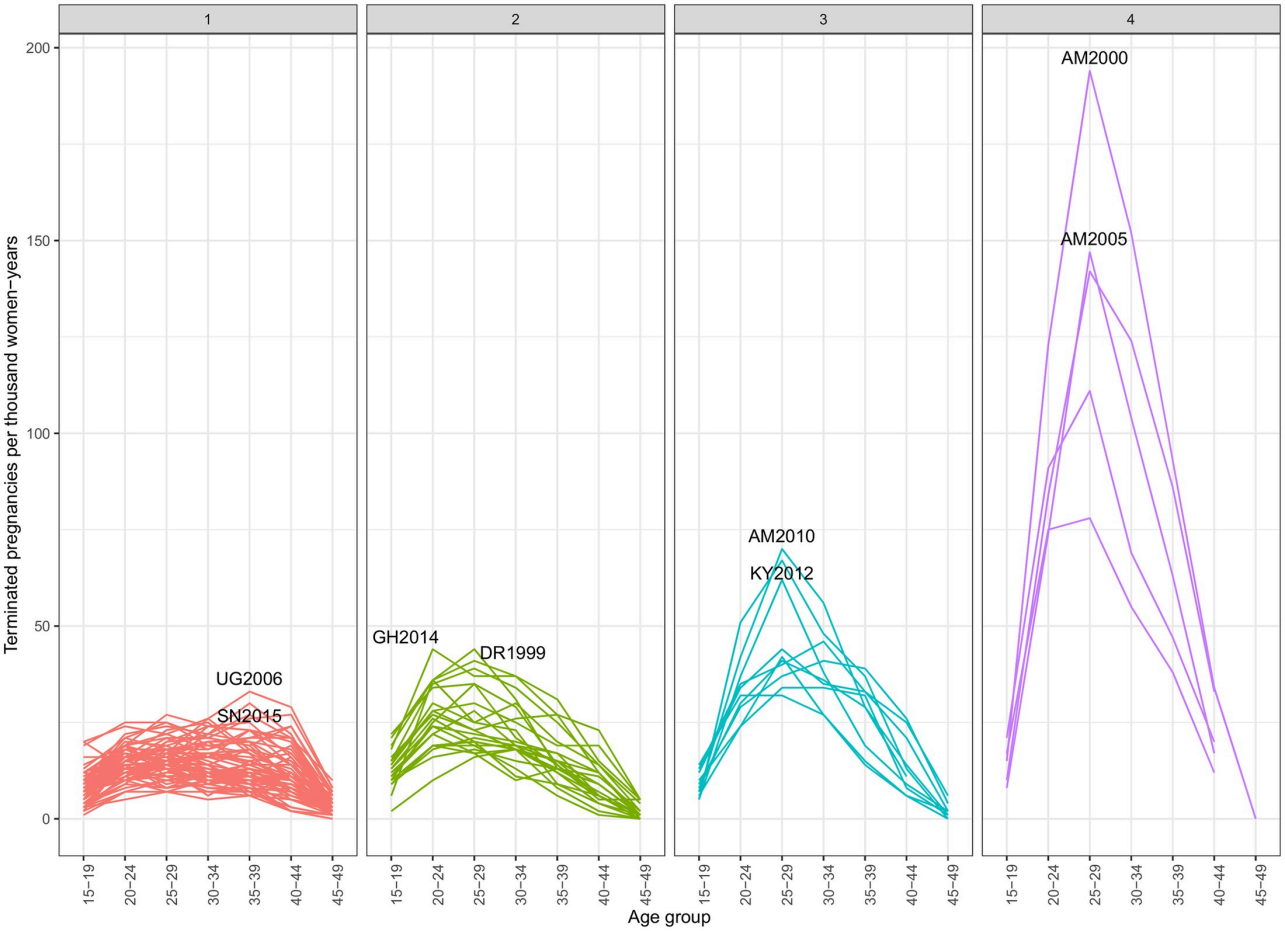
Age-specific termination rate by cluster.

The analysis of *T* suggests that PT are more common among older women consistent both with increased risk of ST and higher prevalence of IA to limit family size. However, there are relatively few pregnancies at older ages and many more pregnancies at peak reproductive ages. When *ASTRs* are computed, we find that termination rates tend to show an inverted U-shaped pattern peaking mostly in the 25-29 age-group for countries with high abortion rates, with more heterogeneity in peak ages for clusters 1 and 2 (Fig 9). Cluster 1 has the lowest *ASTR* and smooth trends by age with maximum values at ages 30-34, although Senegal and Uganda have the highest peaks at ages 35-39. Cluster 2 has the maximum values between the ages of 20-24 and 25-29, especially Ghana and Tajikistan. This suggests that whereas from a medical perspective we should expect a higher likelihood of termination in older pregnant women, from a public health perspective we should expect women experiencing terminations to be younger. Survey-specific *ASTRs* are shown together with the age-specific probabilities of termination in S2 Table and printed in S4 Table.

**Fig 10:**
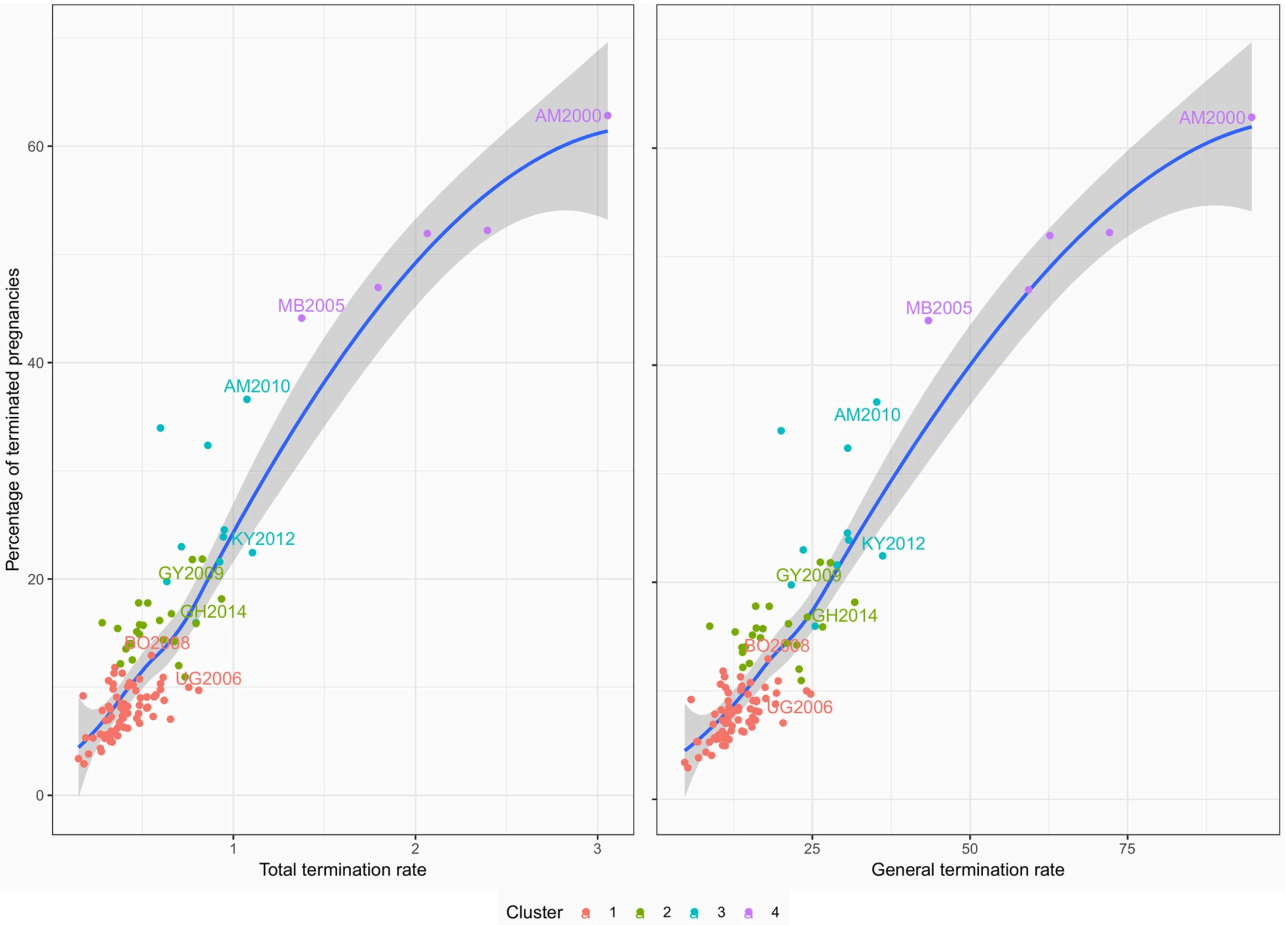
Total termination rate, general termination rate, and probability of pregnancy termination.

Termination rates provide two alternative indicators of the quantum of PT: *TTR* and *GTR*. Fig 10 compares *TTR* and *GTR* with *T*. *TTRs* indicate that in all countries in clusters 1 and 2, women are expected to experience on average less than one pregnancy loss over their reproductive life. *GFR* shows that this corresponds to a risk of less than 25 per thousand of experiencing a termination in a given year. In contrast, in high abortion countries, *TTR* can be higher than two terminations. There is generally a close association between *T* and both *TTR* and *GTR* as captured by the non-parametric regression line. Differences among the three quantum measures are driven by the population structure and the age-structure of women using contraception. *TTR* is not affected by construction by the age-structure, but might still be affected if the age-structure of contraceptors is different from the overall population of women. Note that we can think of *TTR* as the sum of a Total Induced Abortion Rate and a Total Spontaneous Termination Rate. *TPR* can be derived as the sum of *TTR* and *TFR*.

**Fig 11:**
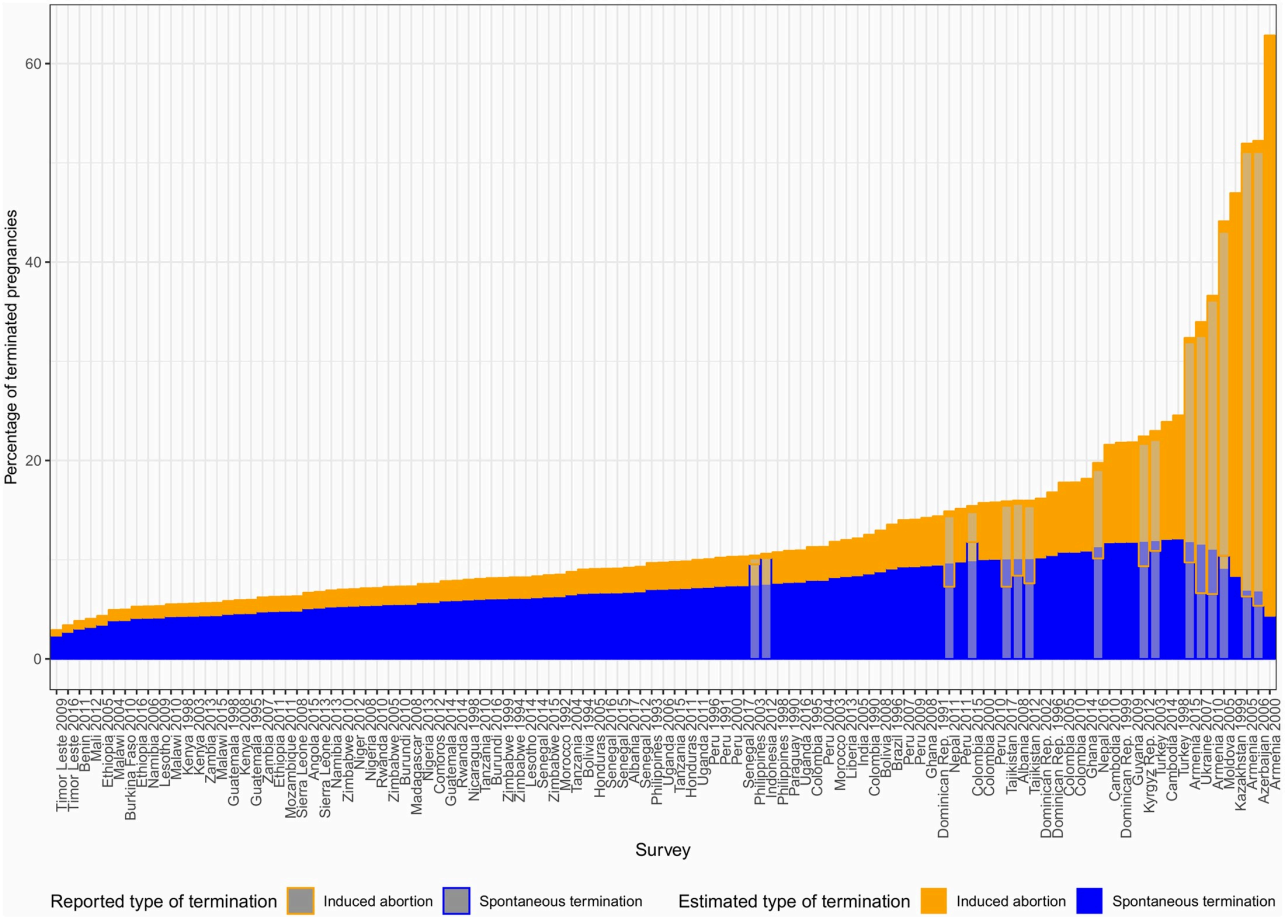
Induced abortion model estimates.

From a reproductive health perspective, the implications and determinants of ST and IA are very different, and it would be interesting to obtain separate estimates of the incidence of ST and IA. As presented in Fig 1, information from the 16 DHS surveys reporting separately IA and ST suggests that differences in IA are mainly driven by differences in *T*. That is the idea behind the proposed logistic regression model for the probability of IA conditional on termination as a function of *T*. Fig 11 presents the resulting IA estimates for all the surveys included in our sample corresponding to model 2. While the model fit is far from perfect, it provides a good approximate indication of the range of likely IA and ST. It suggests that the implicit reported proportion of pregnancies ending in ST increases slowly with *T* up to a maximum of around 10 percent, declining at very high levels of *T* due to competing risks. It also suggests a very low proportion of pregnancies reported to end as IA in countries with low *T*, like in clusters 1 and 2. Note that the gray shadows indicate the observed patterns and the model fits for the surveys reporting the type of outcome. Since there are only two surveys with very low probability of termination, model estimates are driven more by the patterns in surveys with higher values of *T*. For those two surveys the fitted probabilities of IA are higher than the observed values suggesting that the estimates should be taken as an upper bound for reported *IA* in countries with low reported *T*.

**Fig 12:**
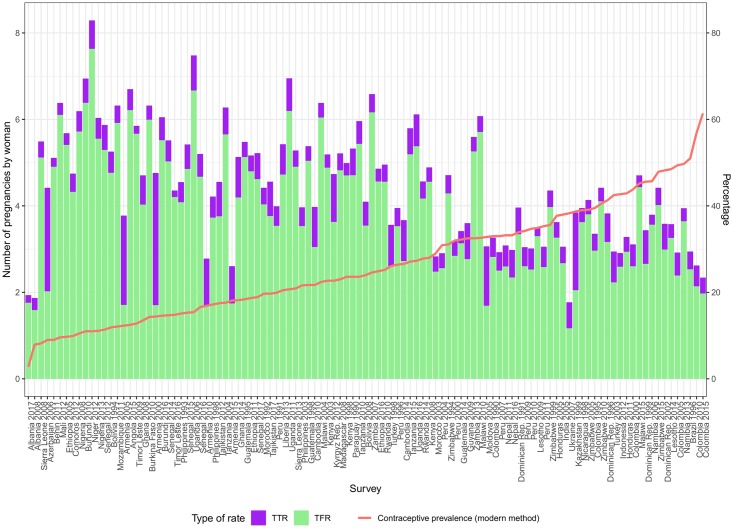
Total pregnancy rate (left-axis) and current contraceptive use of any modern method (right-axis) by survey.

We have finally estimated *TPR* by adding-up *TFR* and *TTR*. Our use of a consistent period for both measures makes this possible. Estimates at the survey level are provided in S6 Table. We can see in Fig 12 that *TPR* is higher in contexts with lower use of modern contraceptives indicating the role of contraception in preventing pregnancies. Once a pregnancy begins, IA provides a final mean of avoiding childbearing. The relative size of the *TFR* and *TTR* in the *TPR* bars indicates these different ways of managing reproduction. Note that our estimates of *TPR* also include reported ST. This will make them higher than alternative estimates only including IA and live-births [5]. On the other hand, those estimates combine DHS estimates of fertility with higher estimates of IA produced by the Guttmacher Institute [3]. While overall increasing levels of modern contraceptive prevalence are associated to a lower number of pregnancies the relation is far from perfect. Other proximate determinants such as union-formation and sexual activity are also expected to play a role.

## Discussion

We have analyzed reported patterns of PT according to age, union status, and contraceptive use prior to pregnancy. This is the first such comparative study based on reproductive calendar history from DHS surveys and including all surveys irrespective of whether the type of pregnancy outcome is reported or not. Moreover, our protocol to select pregnancies makes it possible to relate the estimated conditional probabilities of termination to the age-specific fertility rates in the 3-years before the interview in order to derive consistent estimates of age-specific termination rates, total termination rates, total pregnancy rates, and related measures of reproductive health. Also, the comparison of surveys reporting and not reporting the type of pregnancy termination and from different contexts regarding the legality of abortion helps in the interpretation of the patterns found.

Consistent with expectations and with available evidence [1, 10–12], we find for most surveys, and especially for surveys reporting a high incidence of pregnancy termination, that women that were using contraception at the time of pregnancy and experienced a contraceptive failure are much more likely to report a PT. This suggests increasing likelihood of IA for these women as confirmed in the few surveys reporting the type of termination.

We also find that, while reported termination rates are higher for women using contraception, higher probabilities of termination for contraceptive users move together with higher probabilities for non-contraceptive users. There can be different factors behind this such as differences in the legal framework and the cultural acceptability of abortion. However, there is also the presence, among non-users, of women with unmet need for contraception. Although they are not using contraception, they are not willing to get pregnant. Moreover, in terms of IA, they behave more similar to contraceptive users since in both cases the pregnancy is unintended [51].

Regarding differences according to the legal framework, we find low reported probabilities of termination in all countries with restrictive laws, but there are also countries where abortion is legal reporting low incidence, such as Albania or Tajikistan. While this is consistent with higher levels of underreporting in contexts where IA is not legal, legal consequences could also deter the practice of IA. Differences in the DHS interview protocol might also be behind some of these differences. While we have found no differences according to whether the survey reported IA and ST as separate outcomes, there are grounds for improvement in reporting making sure that the questions are understood, increasing the confidentiality of reporting, or including specific questions on self-administered medication abortion [2, 30, 50, 52].

Little is known behind the drivers of omssions in reported PT and more research is needed to determine to what extent differences in reported patterns are due to underlying differences in PT, in self-awareness of PT, or intentional and unintentional omissions. The use only of the most recent pregnancies in our research should minimize some of the problems connected to omissions that increase with time since the interview [8]. The fact that overall reported levels in ST tend to be stable over time suggests that cultural factors or the functioning of public health systems might be behind these changes [29]. Levels of reported *T* are relatively stable and different surveys from the same country or for neighboring countries tend to fall in the same termination cluster. For the few countries changing cluster adscription over time, external sources suggest that changes in the incidence of IA are behind these changes [49, 50, 53], except in the case of Uganda [54].

Demographic differences in reported PT are important and consistent with previous research [13–17]. For instance, as a woman ages, the probability of PT rises suggesting a higher risk of ST in low abortion countries, and the use of IA for limiting family size in high abortion settings. Also, not-in-union women have higher chances of ending their pregnancies before live-birth. However, these estimates consider exclusively the likelihood rather than the magnitude. In this regard, age-specific termination rates tend to be higher for women aged between 20 and 29 since pregnancy rates are much higher for them.

Cluster and PCA analysis suggest geographic proximity of patterns not only in reported levels but also in differentials according to age, union status, and contraceptive use at the time of pregnancy. However, there is some heterogeneity at the regional level. Latin American and African surveys belong to the two lowest PT clusters. Eurasia reports the maximum levels of PT, showing the largest differentials in countries in the former Soviet Union and where abortion is legal. Countries in insular Southeast Asia report some of the lowest levels. Cluster 2, in particular, shows that some countries reporting low levels of PT tend to report rates that are as high as in cluster 3 for women not-in-union using contraceptives. This suggests the use of IA to prevent out-of-union childbearing.

The use of a consistent framework for PT estimation and fertility estimation has allowed us to move from conditional probabilities of termination to age-specific termination rates, total termination rate, and the total pregnancy rate. While contraceptive use at pregnancy is associated with a higher likelihood of termination at the pregnancy level, the use of efficient contraceptive methods reduces the risk of getting pregnant contributing to a lower total pregnancy rate.

Given the observed pattern that high levels of reported *T* are associated with increasing IA levels, it is possible to interpret differences in *T* as differences in IA. In particular, clusters 3 and 4 include countries reporting high levels of termination and known to be high abortion countries. We propose a simple tentative approach to separate ST and IA based on total PT, based on surveys that report the type of termination. This model suggests that in most DHS surveys, especially those in clusters 1 and 2, reported IA is very low. It also suggests significant differences in reported ST from country to country. While some of these differences can be interpreted, such as low levels in high abortion countries due to competing risks of IA and ST, there is currently a lack of understanding of what lies behind these differences. More research would be needed to address the roles of culture, education, and differential access to reproductive health behind them. The fact that many of the countries reporting the lowest rates of PT are countries with the poorest levels of access to reproductive health, with high maternal mortality and infant mortality and low levels of antenatal care, such as many sub-Saharan African countries, suggests that cultural differences in the self-awareness of PT and clinical monitoring of pregnancies could be behind the differences more than real differences in the risk of PT. More research needs to be done in this respect, mainly due to the increased importance given to more sophisticated indicators of reproductive health, like stillbirth rates, unsafe abortions, or births and abortions prevented by using contraception in international monitoring efforts such as the Family Planning 2020 initiative [32]. Measuring accurately reproductive health indicators is key to well-informed decisions and adequately monitoring the progress in the achievement of internationally agreed objectives, like universal access to reproductive health [31].

Our research also has implications regarding fertility and family planning measurement. In particular, our results suggest the importance of treating separately contraceptive users and non-users when accounting for PT due to the significant connection between contraceptive use and terminations. Such connection is absent, for instance, in the proximate determinants framework of fertility analysis [33, 34]. It is also important to learn more behind the drivers of reported PT. Whereas current international monitoring tends to use DHS surveys for estimation of fertility, contraception, unintended pregnancies, and unmet need, estimates of PT are not used due to concerns regarding their completeness [3, 6, 9]. However, if reported PT is not complete, estimates of unmet need and unintended pregnancies will also not be complete, and the role of contraception in the prevention of pregnancies will be underestimated. While we do not claim reported PT levels to be complete, the patterns reported in this research are at least internally consistent and could be taken as a departure point. Note also that rates reported here are much higher than alternative estimates based on prospective cohort monitoring [28].

## Supporting information

S1 Fig Principal components analysis by categories. (PDF)

S2 Fig Age-specific termination rate (ASTR) and probability of pregnancy termination (T) by survey. (PDF)

S1 Table Calendars used by survey. (PDF)

S2 Table Weighted women by age and union status and weighted pregnancies by outcome. (PDF)

S3 Table Weighted pregnancies (P) and probability of termination (T) by contraceptive use, union status, and age-group. (PDF)

S4 Table Probability of pregnancy termination and age-specific termination rate from reported data. (PDF)

S5 Table Probability of pregnancy termination by age-group, union status, and contraceptive use after the clustering. (PDF)

S6 Table Contraceptive prevalence, total and general rates, and probability of pregnancy termination by survey. TFR: Total fertility rate. TTR: Total termination rate. TPR: Total pregnancy rate. GFR: General fertility rate. GTR: General termination rate. GPR: General pregnancy rate.
(PDF)
