# Supplementary material for: Reported patterns of pregnancy termination from Demographic and Health Surveys
Source: PLoS One. 2019 Aug 19;14(8):e0221178. doi: 10.1371/journal.pone.0221178 (PMC6699730; doi:10.1371/journal.pone.0221178)
Supplement: S1 Fig — (PDF) [file pone.0221178.s001.pdf]

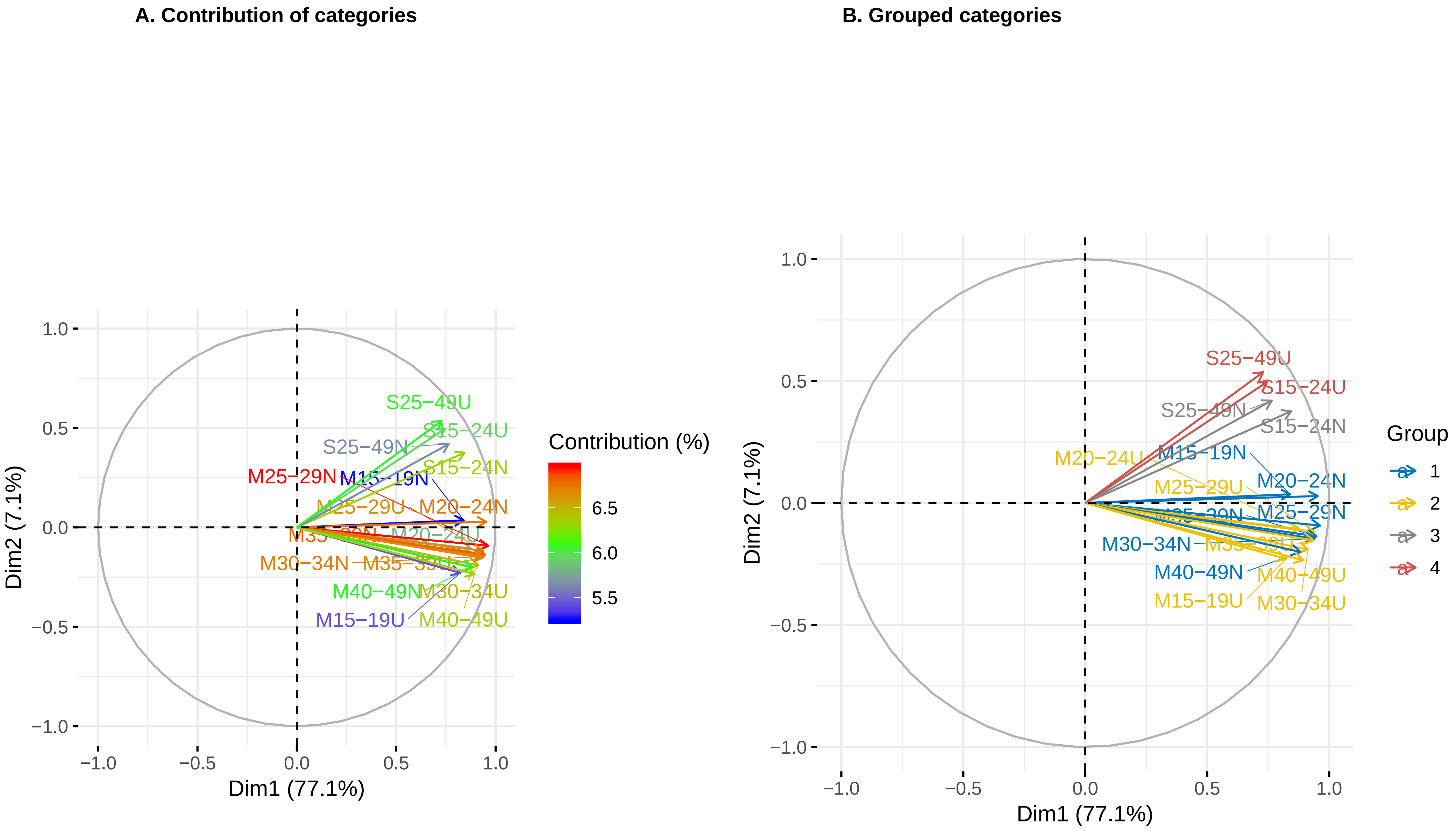

Note: The category name has the following structure: The first letter is the union status (M=in−union or S=not−in−union). Then, the age−group. The last letter refers to using (U) or not using (N) contraceptives.
