# Supplementary material for: Reported patterns of pregnancy termination from Demographic and Health Surveys
Source: PLoS One. 2019 Aug 19;14(8):e0221178. doi: 10.1371/journal.pone.0221178 (PMC6699730; doi:10.1371/journal.pone.0221178)
Supplement: S2 Fig — (PDF) [file pone.0221178.s002.pdf]

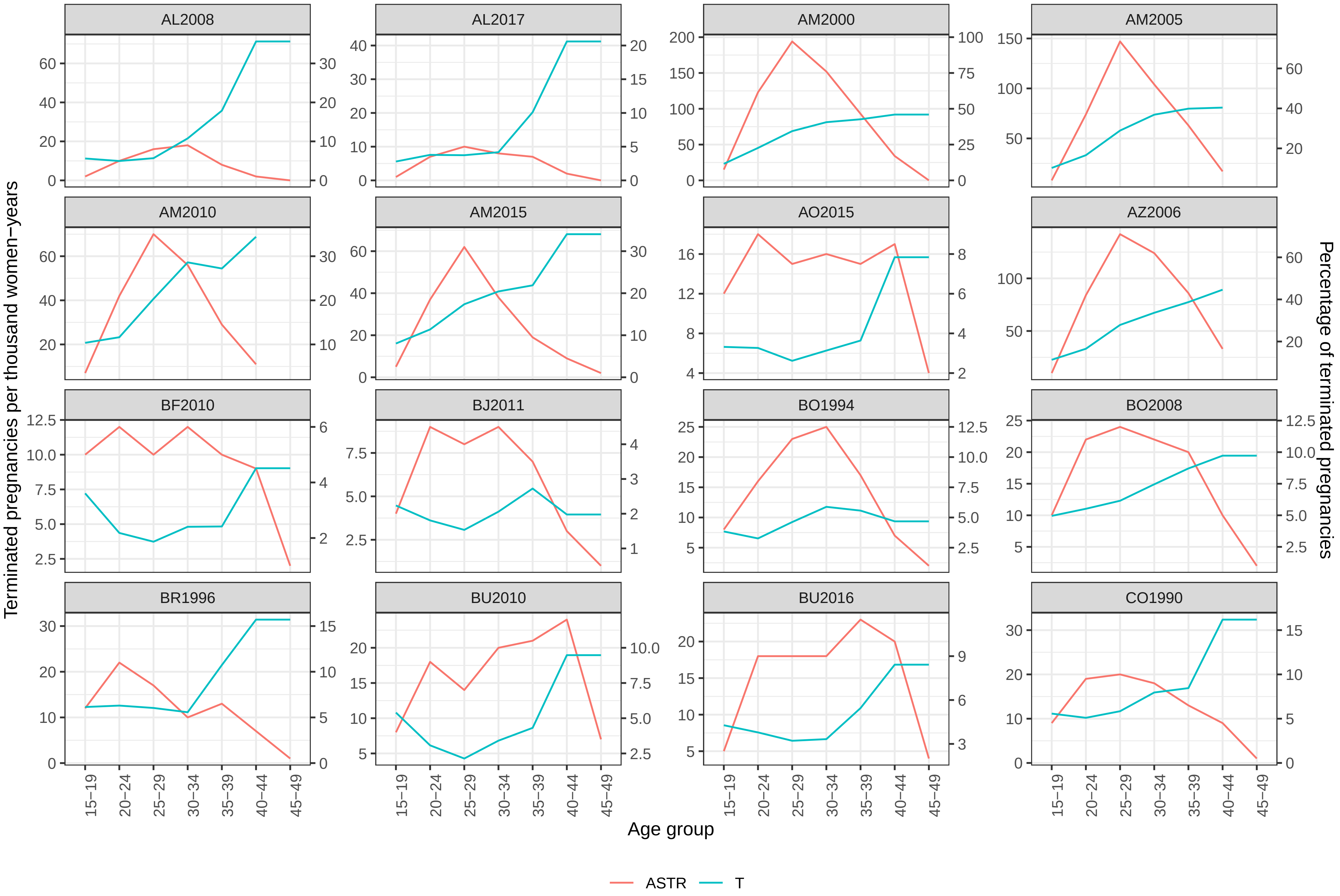
AL2008
AL2017
AM2000
AM2005
AM2010
AM2015
AO2015
AZ2006
BF2010
BJ2011
BO1994
BO2008
BR1996
BU2010
BU2016
CO1990
Terminated pregnancies per thousand women−years
Percentage of terminated pregnancies
15−19
20−24
25−29
30−34
35−39
40−44
45−49
Age group
ASTR
T

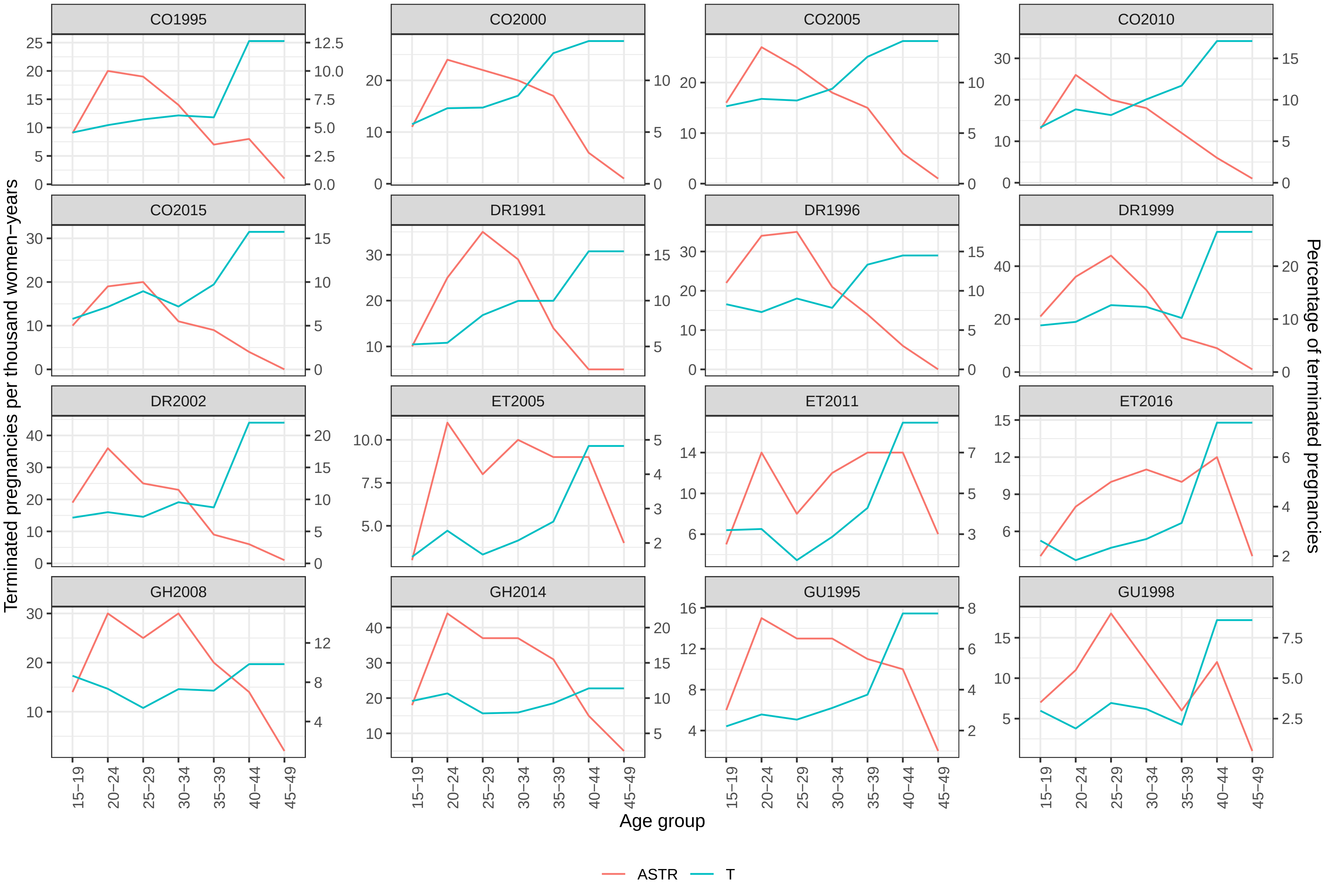
CO1995
CO2000
CO2005
CO2010
CO2015
DR1991
DR1996
DR1999
DR2002
ET2005
ET2011
ET2016
GH2008
GH2014
GU1995
GU1998
Terminated pregnancies per thousand women−years
Percentage of terminated pregnancies
15−19
20−24
25−29
30−34
35−39
40−44
45−49
Age group
ASTR
T

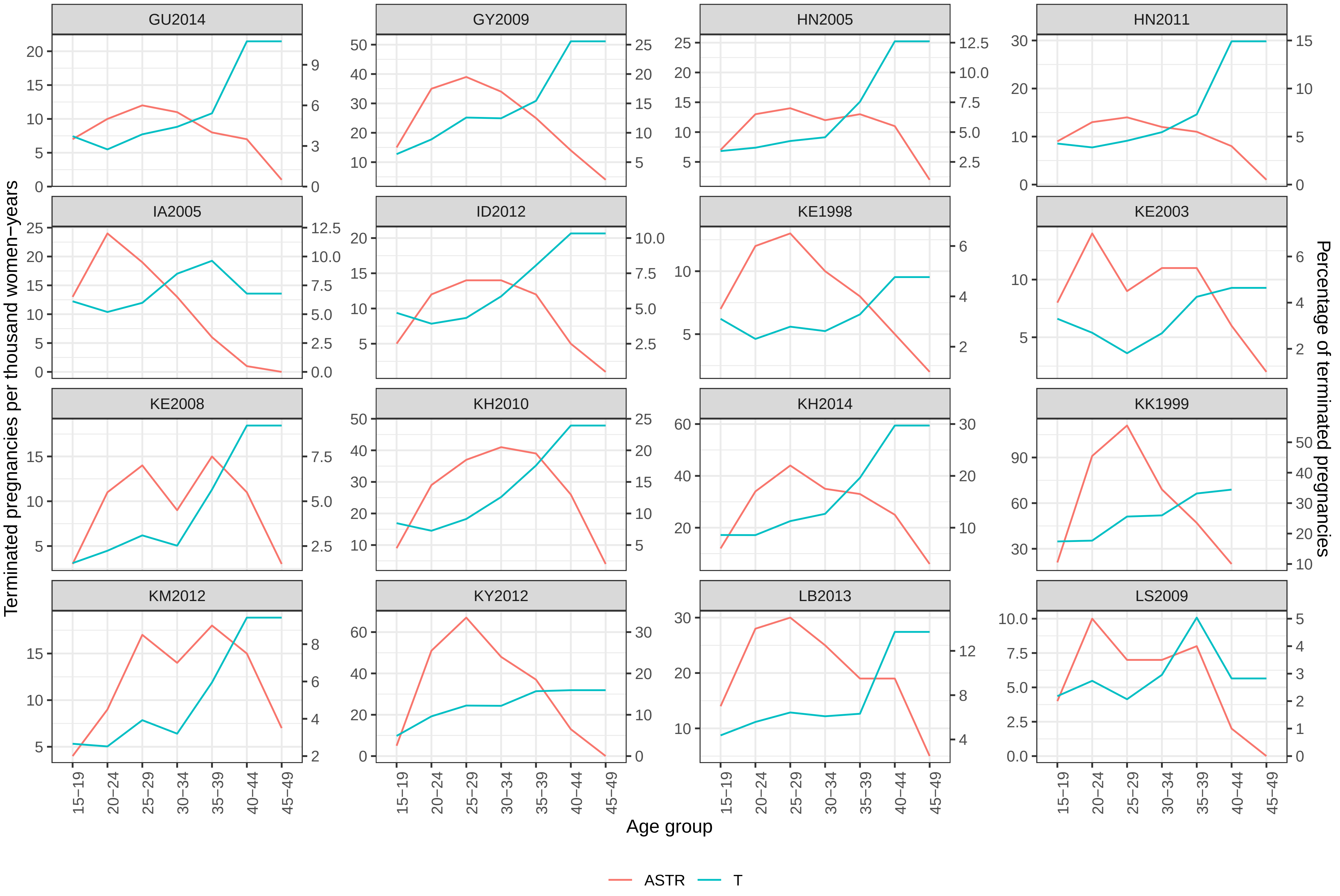

GU2014
GY2009
HN2005
HN2011
IA2005
ID2012
KE1998
KE2003
KE2008
KH2010
KH2014
KK1999
KM2012
KY2012
LB2013
LS2009
Terminated pregnancies per thousand women−years
Percentage of terminated pregnancies
Age group
15−19
20−24
25−29
30−34
35−39
40−44
45−49
ASTR
T

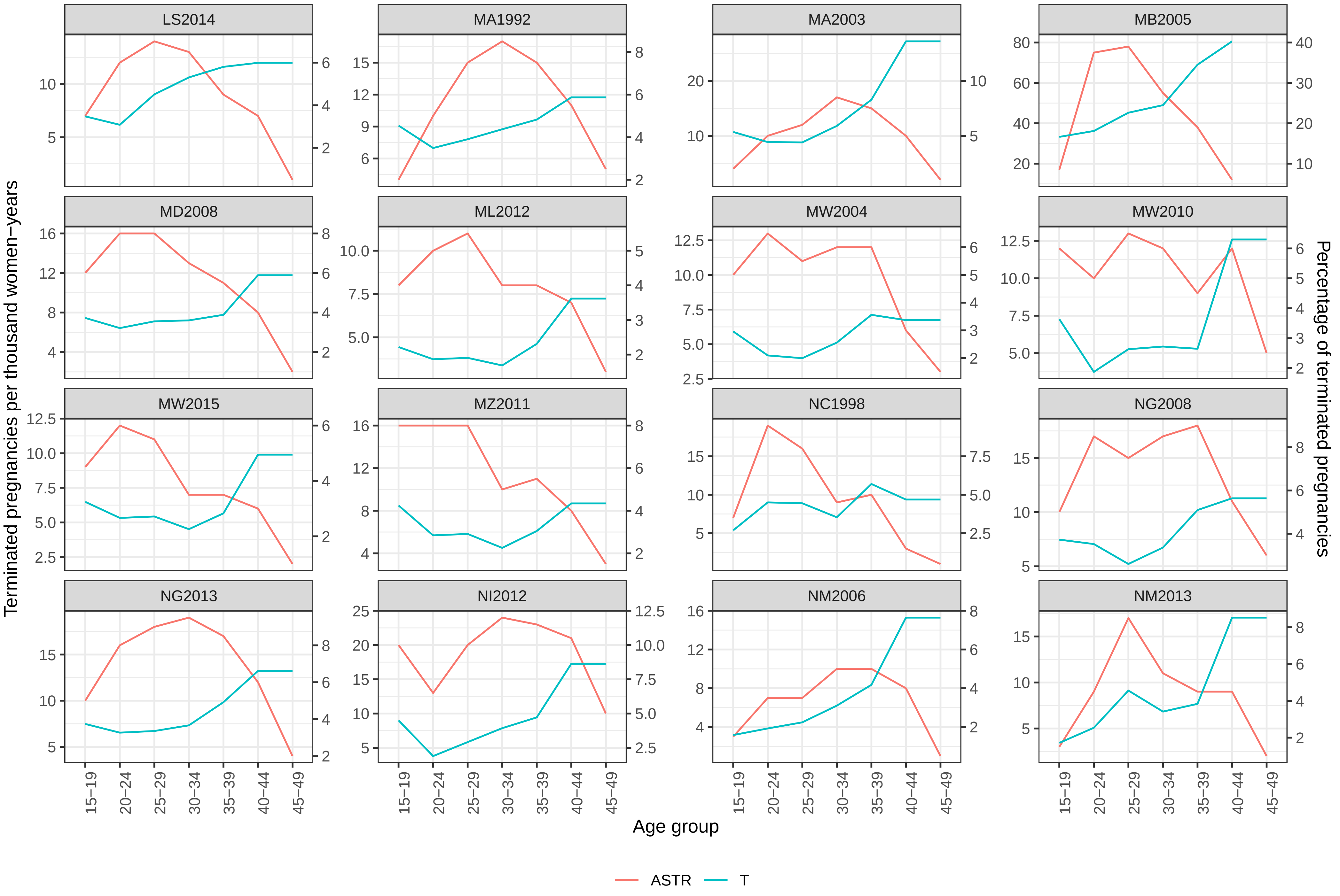
LS2014
MA1992
MA2003
MB2005
MD2008
ML2012
MW2004
MW2010
MW2015
MZ2011
NC1998
NG2008
NG2013
NI2012
NM2006
NM2013
Terminated pregnancies per thousand women−years
Percentage of terminated pregnancies
Age group
15−19
20−24
25−29
30−34
35−39
40−44
45−49
ASTR
T

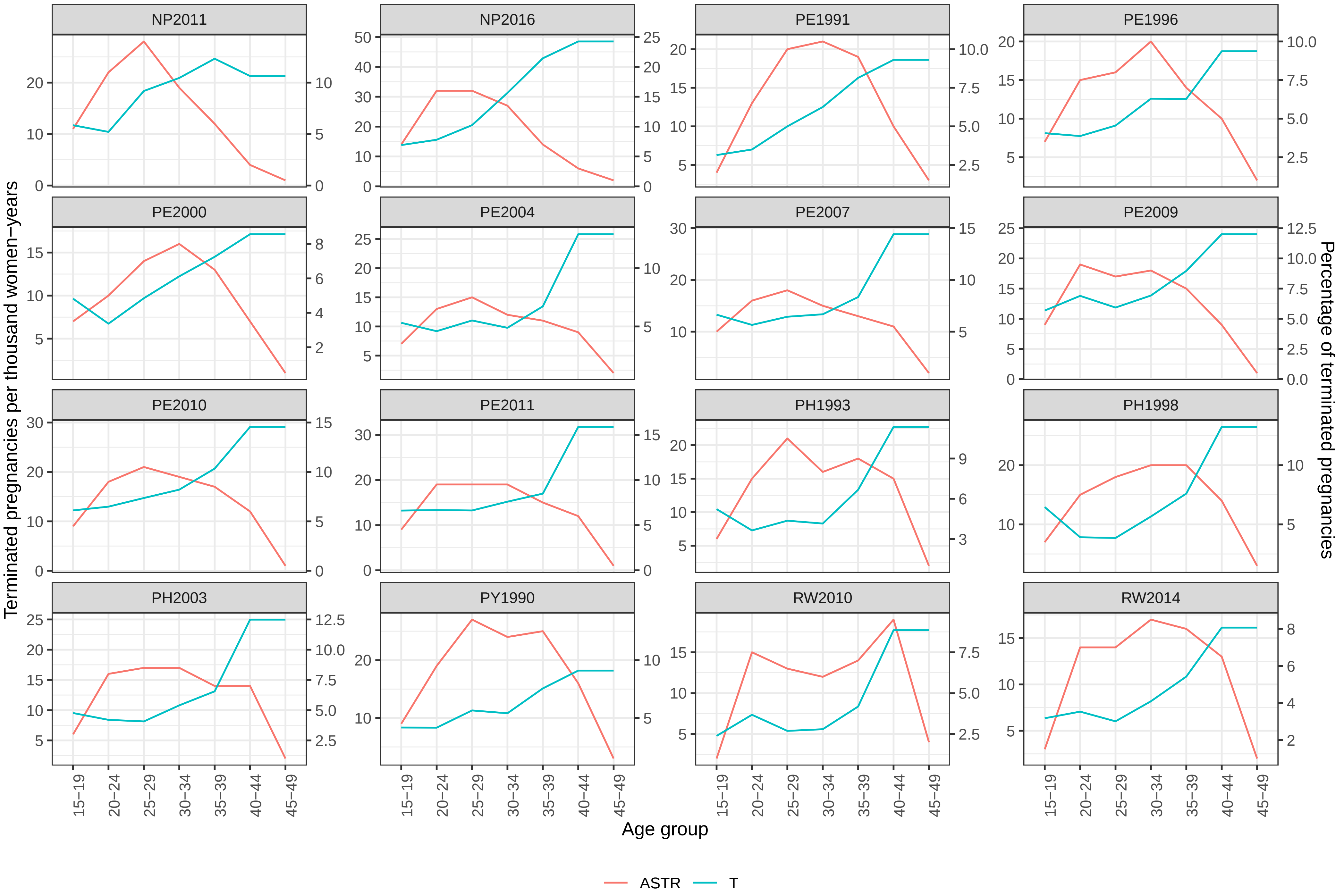
NP2011
NP2016
PE1991
PE1996
PE2000
PE2004
PE2007
PE2009
PE2010
PE2011
PH1993
PH1998
PH2003
PY1990
RW2010
RW2014
15−19
20−24
25−29
30−34
35−39
40−44
45−49
Age group
Terminated pregnancies per thousand women−years
Percentage of terminated pregnancies
ASTR
T

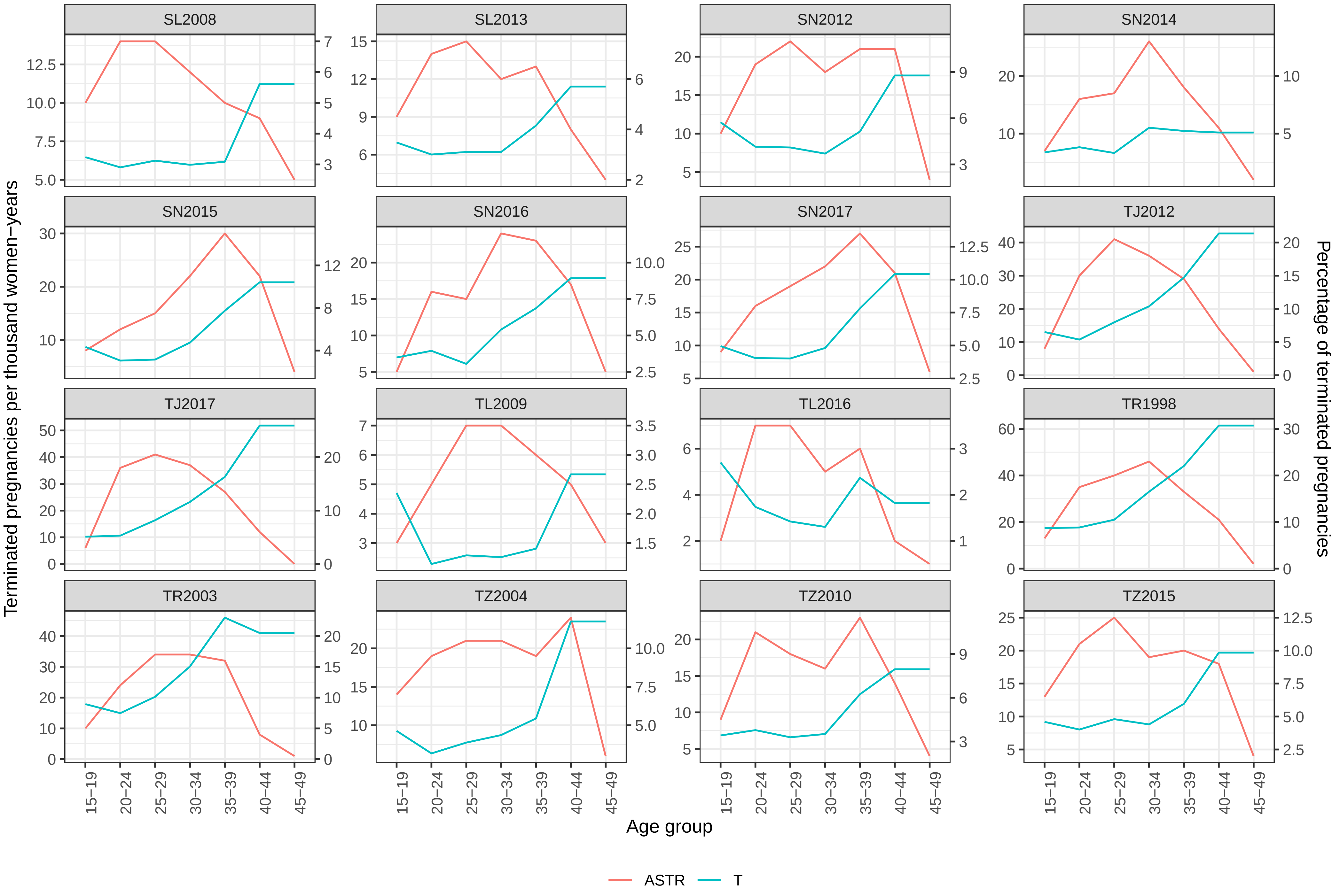
SL2008
SL2013
SN2012
SN2014
SN2015
SN2016
SN2017
TJ2012
TJ2017
TL2009
TL2016
TR1998
TR2003
TZ2004
TZ2010
TZ2015
Terminated pregnancies per thousand women−years
Percentage of terminated pregnancies
15−19
20−24
25−29
30−34
35−39
40−44
45−49
Age group
ASTR
T

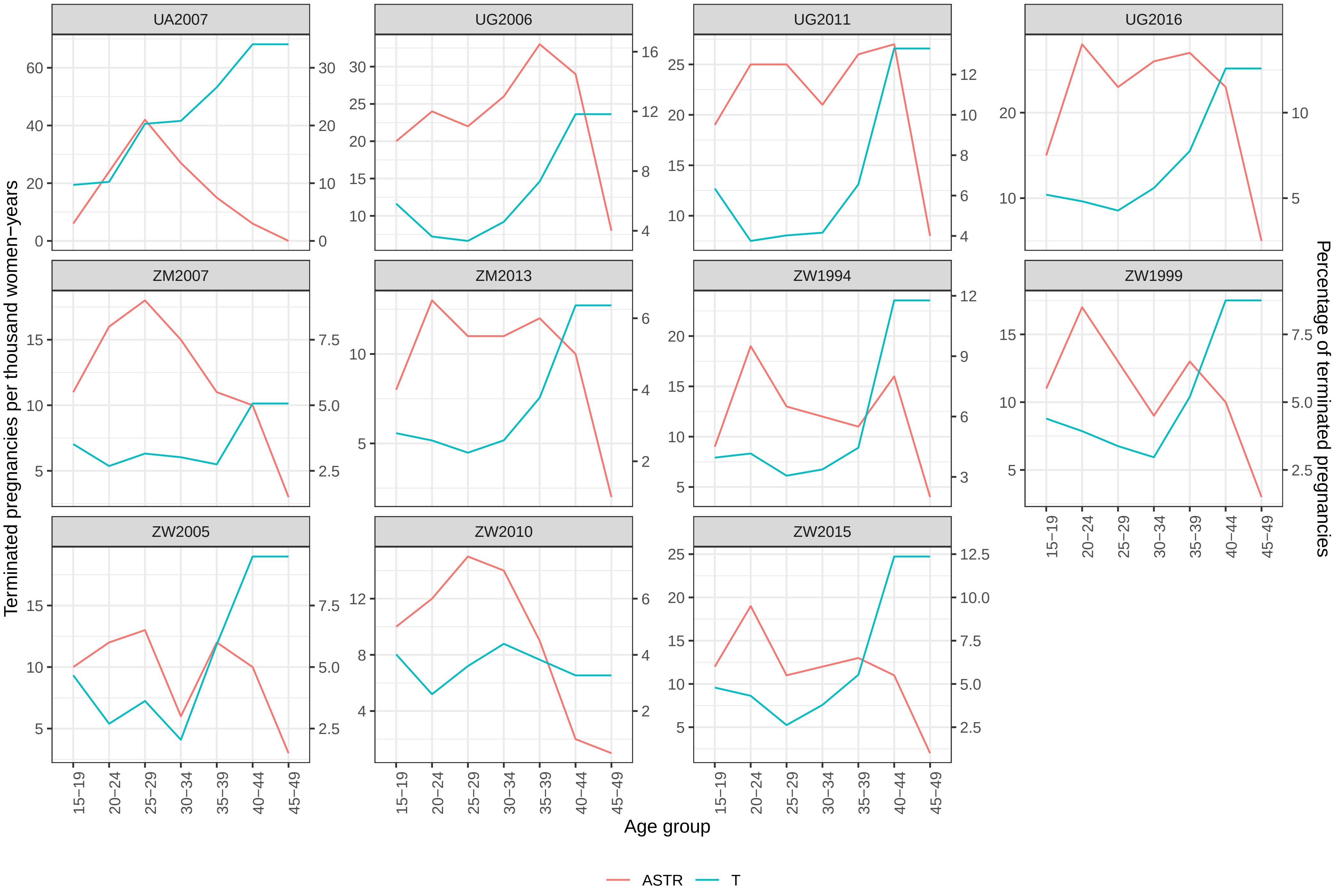

UA2007
UG2006
UG2011
UG2016
ZM2007
ZM2013
ZW1994
ZW1999
ZW2005
ZW2010
ZW2015
Terminated pregnancies per thousand women−years
Percentage of terminated pregnancies
Age group
15−19
20−24
25−29
30−34
35−39
40−44
45−49
ASTR
T
