# Supplementary material for: Reported patterns of pregnancy termination from Demographic and Health Surveys
Source: PLoS One. 2019 Aug 19;14(8):e0221178. doi: 10.1371/journal.pone.0221178 (PMC6699730; doi:10.1371/journal.pone.0221178)
Supplement: S1 Table — (PDF) [file pone.0221178.s003.pdf]

Code	Survey	cal1	cal2	cal3
**Africa**				
AO	Angola 2015	X	X	
BF	Burkina Faso 2010	X	X	
BJ	Benin 2011	X	X	
BU	Burundi 2010	X	X	
BU	Burundi 2016	X	X	
ET	Ethiopia 2005	X	X	X
ET	Ethiopia 2011	X		
ET	Ethiopia 2016	X	X	
GH	Ghana 2008	X		
GH	Ghana 2014	X	X	
KE	Kenya 1998	X	X	X
KE	Kenya 2003	X	X	X
KE	Kenya 2008	X		
KM	Comoros 2012	X	X	
LB	Liberia 2013	X	X	
LS	Lesotho 2009	X		
LS	Lesotho 2014	X	X	
MA	Morocco 1992	X	X	X
MA	Morocco 2003	X	X	
MD	Madagascar 2008	X		
ML	Mali 2012	X	X	
MW	Malawi 2004	X	X	X
MW	Malawi 2010	X		
MW	Malawi 2015	X	X	
MZ	Mozambique 2011	X	X	
NG	Nigeria 2008	X		
NG	Nigeria 2013	X	X	
NI	Niger 2012	X	X	
NM	Namibia 2006	X		
RW	Rwanda 2010	X	X	
RW	Rwanda 2014	X	X	
SL	Sierra Leone 2008	X		
SL	Sierra Leone 2013	X	X	
SN	Senegal 2012	X	X	
SN	Senegal 2014	X	X	
SN	Senegal 2015	X	X	
SN	Senegal 2016	X	X	
SN	Senegal 2017	X	X	
TZ	Tanzania 2004	X	X	X
TZ	Tanzania 2010	X		
TZ	Tanzania 2015	X	X	
UG	Uganda 2006	X		
UG	Uganda 2011	X	X	
UG	Uganda 2016	X	X	
ZM	Zambia 2007	X		
ZM	Zambia 2013	X	X	
ZW	Zimbabwe 1994	X	X	X
ZW	Zimbabwe 1999	X	X	X

ZW	Zimbabwe 2005	X	X	X
ZW	Zimbabwe 2010	X	X	
ZW	Zimbabwe 2015	X	X	
**Central and West Asia & Europe**				
AL	Albania 2008	X		
AL	Albania 2017	X	X	
AM	Armenia 2000	X	X	X
AM	Armenia 2005	X	X	X
AM	Armenia 2010	X	X	
AM	Armenia 2015	X	X	
AZ	Azerbaijan 2006	X	X	
KK	Kazakhstan 1999	X	X	X
KY	Kyrgyz Republic 2012	X	X	
MB	Moldova 2005	X	X	X
TJ	Tajikistan 2012	X	X	
TJ	Tajikistan 2017	X	X	
TR	Turkey 1998	X	X	X
TR	Turkey 2003	X	X	X
UA	Ukraine 2007	X	X	
**Latin America**				
BO	Bolivia 1994	X	X	X
BO	Bolivia 1998	X		
BR	Brazil 1996	X	X	X
CO	Colombia 1990	X	X	X
CO	Colombia 1995	X	X	X
CO	Colombia 2000	X	X	X
CO	Colombia 2005	X	X	X
CO	Colombia 2010	X	X	
CO	Colombia 2015	X	X	
DR	Dominican Republic 1991	X	X	X
DR	Dominican Republic 1996	X	X	X
DR	Dominican Republic 1999	X	X	X
DR	Dominican Republic 2002	X	X	X
GU	Guatemala 1995	X	X	X
GU	Guatemala 1998	X	X	X
GU	Guatemala 2014	X	X	
GY	Guyana 2009	X		
HN	Honduras 2005	X		
HN	Honduras 2011	X	X	
NC	Nicaragua 1998	X	X	X
PE	Peru 1991	X	X	X
PE	Peru 1996	X	X	X
PE	Peru 2000	X	X	X
PE	Peru 2004	X	X	X
PE	Peru 2007	X	X	X
PE	Peru 2009	X	X	X
PE	Peru 2010	X	X	X
PE	Peru 2011	X	X	X
PY	Paraguay 1990	X	X	X

South and Southeast Asia

IA	India 2005	X	X	X
ID	Indonesia 2012	X	X	X
KH	Cambodia 2010	X	X	
KH	Cambodia 2014	X	X	
NP	Nepal 2011	X	X	
NP	Nepal 2016	X	X	
PH	Philippines 1993	X	X	X
PH	Philippines 1998	X	X	X
PH	Philippines 2003	X	X	X
TL	Timor Leste 2009	X		
TL	Timor Leste 2016	X	X	
