# Supplementary material for: Reported patterns of pregnancy termination from Demographic and Health Surveys
Source: PLoS One. 2019 Aug 19;14(8):e0221178. doi: 10.1371/journal.pone.0221178 (PMC6699730; doi:10.1371/journal.pone.0221178)
Supplement: S2 Table — (PDF) [file pone.0221178.s004.pdf]

Code	Survey	Women	Percentage of women							Pregnancies	Pregnancies ending in	
			In-union	15-19	20-24	25-29	30-34	35-39	40-49		Birth	Termination
**Africa**												
AO	Angola 2015	25,567	65.2	25.2	21.8	17.7	12.3	11.6	11.3	8,880	8,288	592
BF	Burkina Faso 2010	31,132	82.9	21.0	20.1	18.6	14.5	12.0	13.8	10,029	9,530	499
BJ	Benin 2011	29,692	77.6	18.9	20.4	19.8	16.3	12.6	12.1	8,253	7,937	316
BU	Burundi 2010	16,403	65.4	27.4	20.9	16.5	11.6	10.7	12.8	5,428	5,032	396
BU	Burundi 2016	30,485	65.3	23.5	20.7	16.3	14.7	11.3	13.4	9,060	8,321	739
ET	Ethiopia 2005	23,964	68.2	26.7	21.4	17.6	12.4	10.8	11.1	7,078	6,770	308
ET	Ethiopia 2011	29,672	75.1	24.5	21.3	18.5	13.6	11.1	11.0	7,506	7,036	470
ET	Ethiopia 2016	27,528	73.6	24.4	19.1	19.7	14.3	12.2	10.3	7,006	6,636	370
GH	Ghana 2008	8,859	69.6	22.3	19.4	16.7	14.6	12.9	14.2	2,097	1,799	298
GH	Ghana 2014	17,169	69.2	18.6	18.7	17.9	14.8	13.8	16.1	4,390	3,593	797
KE	Kenya 1998	13,636	64.4	25.3	20.6	17.3	14.1	12.1	10.5	3,748	3,540	208
KE	Kenya 2003	14,857	61.2	24.8	21.0	17.7	13.4	10.9	12.2	4,034	3,809	225
KE	Kenya 2008	15,151	70.1	23.0	21.0	17.9	13.8	11.1	13.2	3,895	3,664	231
KM	Comoros 2012	9,059	69.3	26.4	19.1	18.2	13.5	11.8	11.0	2,205	2,038	167
LB	Liberia 2013	16,786	76.3	20.8	18.4	17.8	14.2	13.4	15.4	4,599	4,047	552
LS	Lesotho 2009	13,521	66.6	25.0	21.0	15.9	12.7	10.3	15.1	2,530	2,395	135
LS	Lesotho 2014	11,764	65.3	24.8	20.5	16.9	13.8	10.9	13.1	2,253	2,068	185
MA	Morocco 1992	14,145	67.7	15.6	22.9	17.0	16.7	13.2	14.5	3,445	3,152	293
MA	Morocco 2003	30,068	60.2	21.9	18.9	16.4	12.5	12.2	18.1	4,123	3,636	487
MD	Madagascar 2008	31,458	80.9	23.7	17.4	17.4	14.2	12.7	14.6	8,297	7,690	607
ML	Mali 2012	18,960	85.9	21.6	19.7	21.4	14.8	11.4	11.1	6,392	6,133	259
MW	Malawi 2004	20,692	73.1	25.9	24.4	15.7	13.1	10.1	10.7	7,235	6,877	358
MW	Malawi 2010	41,117	82.5	21.7	21.6	19.2	14.6	11.0	11.9	13,049	12,329	720
MW	Malawi 2015	43,386	77.7	23.8	20.9	18.1	15.8	11.7	9.6	11,077	10,450	627

Code	Survey	Women	Percentage of women							Pregnancies	Pregnancies ending in	
			In-union	15-19	20-24	25-29	30-34	35-39	40-49		Birth	Termination
MZ	Mozambique 2011	24,487	77.8	22.4	20.0	18.0	13.9	12.1	13.6	7,888	7,392	496
NG	Nigeria 2008	61,182	75.6	22.0	20.7	18.8	13.2	11.7	13.6	18,702	17,370	1,332
NG	Nigeria 2013	70,955	75.2	21.8	19.2	18.6	13.7	12.0	14.8	21,249	19,642	1,607
NI	Niger 2012	19,981	88.9	21.2	20.1	21.8	14.3	11.1	11.5	8,955	8,325	630
NM	Namibia 2006	17,254	43.3	23.9	20.1	17.3	14.0	11.9	12.7	3,385	3,205	180
NM	Namibia 2013	16,361	42.1	22.5	19.9	17.1	14.7	12.0	13.8	3,312	3,083	229
RW	Rwanda 2010	24,554	61.9	22.4	21.7	18.2	12.9	10.4	14.4	5,835	5,418	417
RW	Rwanda 2014	24,480	61.9	21.0	19.4	18.5	16.0	11.6	13.5	5,556	5,118	438
SL	Sierra Leone 2008	13,396	79.7	20.4	20.8	20.3	15.5	12.0	11.1	3,946	3,697	249
SL	Sierra Leone 2013	28,995	74.8	23.3	18.3	18.0	15.0	12.9	12.5	7,952	7,414	538
SN	Senegal 2012	15,240	71.0	26.6	21.7	17.2	12.6	10.1	11.7	4,419	4,008	411
SN	Senegal 2014	14,926	72.6	25.1	20.9	18.6	13.2	10.5	11.7	4,188	3,839	349
SN	Senegal 2015	15,692	71.9	25.8	19.8	18.9	12.7	11.4	11.5	4,294	3,903	391
SN	Senegal 2016	15,709	72.9	25.4	20.6	17.7	14.0	10.6	11.8	4,115	3,741	374
SN	Senegal 2017	29,760	71.2	25.0	18.7	18.6	14.6	11.6	11.5	7,728	6,930	798
TZ	Tanzania 2004	18,442	67.8	23.1	20.6	18.3	14.5	10.8	12.6	6,052	5,520	532
TZ	Tanzania 2010	18,097	75.0	22.1	19.6	17.1	14.3	12.6	14.3	5,535	5,088	447
TZ	Tanzania 2015	23,887	73.3	23.5	19.1	16.6	13.9	12.7	14.3	6,999	6,314	685
UG	Uganda 2006	15,203	78.2	23.2	20.3	17.8	14.6	11.3	12.8	5,778	5,217	561
UG	Uganda 2011	15,543	75.0	23.7	21.1	18.4	13.5	11.6	11.6	5,572	5,015	557
UG	Uganda 2016	33,314	73.9	24.4	20.7	17.8	13.4	11.4	12.3	10,528	9,375	1,153
ZM	Zambia 2007	12,682	73.8	23.2	22.6	19.1	14.0	10.3	10.7	4,384	4,112	272
ZM	Zambia 2013	29,627	72.3	23.9	18.9	18.2	14.8	12.2	12.0	8,592	8,108	484
ZW	Zimbabwe 1994	10,776	64.9	25.5	20.7	16.7	13.7	11.5	12.0	2,645	2,427	218
ZW	Zimbabwe 1999	9,872	62.5	28.4	23.2	16.9	9.0	10.8	11.6	2,452	2,252	200
ZW	Zimbabwe 2005	15,481	61.1	27.2	22.0	16.9	12.5	9.6	11.8	3,557	3,298	259

Code	Survey	Women	Percentage of women							Pregnancies	Pregnancies ending in	
			In-union	15-19	20-24	25-29	30-34	35-39	40-49		Birth	Termination
ZW	Zimbabwe 2010	16,255	72.0	23.9	21.8	18.5	14.8	10.8	10.2	3,981	3,702	279
ZW	Zimbabwe 2015	17,660	73.2	21.3	19.2	18.5	16.5	12.7	11.8	4,207	3,851	356
**Central and West Asia & Europe**												
AL	Albania 2008	11,904	69.4	20.4	14.7	11.9	15.5	19.6	18.0	1,049	882	167
AL	Albania 2017	17,926	80.7	9.6	16.4	14.1	15.1	16.1	28.7	1,767	1,604	163
AM	Armenia 2000	11,234	70.3	19.5	15.8	12.4	14.6	16.7	21.0	2,508	932	1,576
AM	Armenia 2005	9,783	75.2	12.6	15.2	14.8	14.3	17.0	26.2	2,035	978	1,057
AM	Armenia 2010	9,427	74.8	11.2	21.7	16.0	14.7	13.8	22.5	1,508	956	552
AM	Armenia 2015	10,568	76.1	8.0	19.2	19.8	17.1	15.3	20.7	1,549	1,048	501
AZ	Azerbaijan 2006	14,366	67.3	20.7	17.5	14.3	14.1	15.9	17.5	3,121	1,491	1,630
KK	Kazakhstan 1999	8,507	65.4	17.8	14.9	16.9	16.4	16.2	17.8	1,613	856	757
KY	Kyrgyz Republic 2012	14,831	73.6	20.1	19.7	15.9	13.1	12.5	18.8	3,436	2,665	771
MB	Moldova 2005	13,033	67.5	20.9	16.0	14.4	13.5	12.9	22.2	1,854	1,036	818
TJ	Tajikistan 2012	17,680	69.9	22.8	20.3	15.7	12.6	11.8	16.7	4,111	3,455	656
TJ	Tajikistan 2017	19,554	74.5	20.4	19.5	18.5	13.9	12.1	15.6	4,850	4,079	771
TR	Turkey 1998	13,319	81.3	15.1	18.3	17.5	17.8	14.7	16.5	2,860	2,158	702
TR	Turkey 2003	15,300	94.8	5.6	15.8	19.8	17.8	17.8	23.2	3,200	2,464	736
UA	Ukraine 2007	12,342	76.9	14.3	15.7	16.2	16.0	15.9	21.8	1,061	701	360
**Latin America**												
BO	Bolivia 1994	15,303	64.5	21.9	18.9	17.4	15.5	12.5	13.7	4,086	3,718	368
BO	Bolivia 2008	31,082	67.9	21.2	17.6	16.9	14.2	13.0	17.2	6,217	5,412	805
BR	Brazil 1996	22,715	63.1	20.2	16.9	17.3	15.8	13.7	16.2	3,386	2,927	459
CO	Colombia 1990	15,418	64.9	22.3	21.5	19.0	14.5	11.1	11.6	2,684	2,348	336
CO	Colombia 1995	20,150	57.8	19.8	19.3	17.7	14.2	13.4	15.6	3,543	3,143	400
CO	Colombia 2000	21,255	54.6	21.0	17.3	16.1	15.3	14.0	16.3	3,350	2,823	527

Code	Survey	Women	Percentage of women							Pregnancies	Pregnancies ending in	
			In-union	15-19	20-24	25-29	30-34	35-39	40-49		Birth	Termination
CO	Colombia 2005	70,147	55.4	19.0	17.6	15.3	14.8	14.3	19.0	10,185	8,374	1,811
CO	Colombia 2010	89,239	70.5	19.5	16.8	16.1	14.5	14.3	18.8	11,639	9,568	2,071
CO	Colombia 2015	66,362	71.3	18.7	16.9	15.6	14.9	13.4	20.5	7,807	6,603	1,204
DR	Dominican Republic 1991	12,546	63.4	25.5	22.5	17.7	14.3	11.0	9.0	2,877	2,463	414
DR	Dominican Republic 1996	14,905	65.3	22.9	20.1	17.2	14.7	12.9	12.3	3,255	2,709	546
DR	Dominican Republic 1999	2,028	62.6	24.1	21.3	19.4	16.9	7.7	10.6	435	340	95
DR	Dominican Republic 2002	41,477	67.7	21.7	18.4	16.6	14.9	13.9	14.4	8,065	6,761	1,304
GU	Guatemala 1995	21,716	70.9	24.3	19.2	14.8	13.6	12.8	15.4	6,179	5,811	368
GU	Guatemala 1998	10,598	71.4	24.7	19.8	15.8	13.3	12.6	13.9	2,988	2,813	175
GU	Guatemala 2014	47,045	68.1	23.1	19.1	16.0	14.2	12.3	15.4	8,300	7,649	651
GY	Guyana 2009	8,916	70.9	20.3	15.6	15.2	14.7	14.8	19.3	1,567	1,225	342
HN	Honduras 2005	36,022	73.7	23.3	19.6	16.4	13.9	11.7	15.0	6,767	6,154	613
HN	Honduras 2011	41,241	72.6	23.2	19.0	16.3	14.3	12.1	15.0	7,120	6,420	700
NC	Nicaragua 1998	23,629	67.4	25.1	19.2	17.3	14.6	12.0	11.7	5,145	4,734	411
PE	Peru 1991	28,575	59.7	23.1	19.8	16.9	14.7	12.4	13.1	5,696	5,114	582
PE	Peru 1996	52,860	63.8	21.5	18.9	17.3	15.1	12.7	14.5	10,459	9,408	1,051
PE	Peru 2000	50,579	62.2	20.7	18.1	16.4	15.5	13.5	15.8	8,027	7,201	826
PE	Peru 2004	34,361	61.3	19.6	17.3	16.3	15.6	14.6	16.6	4,531	4,019	512
PE	Peru 2007	40,992	62.6	18.7	16.7	15.9	15.5	13.2	19.9	5,949	5,116	833
PE	Peru 2009	44,210	63.1	18.7	16.5	15.9	15.4	14.7	18.9	6,514	5,599	915
PE	Peru 2010	41,908	62.6	18.5	16.3	15.9	15.7	14.7	19.0	6,115	5,150	965
PE	Peru 2011	40,991	63.2	18.1	16.1	16.0	15.8	14.8	19.2	6,109	5,184	925
PY	Paraguay 1990	10,530	64.6	22.4	19.1	17.5	14.2	12.1	14.6	2,789	2,485	304
**South and Southeast Asia**												
IA	India 2005	227,719	72.6	21.3	19.4	17.3	15.1	13.0	14.0	38,223	33,576	4,647
ID	Indonesia 2012	84,923	71.9	16.1	15.9	17.1	16.0	15.4	19.5	11,858	10,600	1,258

			Percentage of women								Pregnancies ending in	
Code	Survey	Women	In-union	15-19	20-24	25-29	30-34	35-39	40-49	Pregnancies	Birth	Termination
KH	Cambodia 2010	33,889	69.9	21.2	18.7	18.2	10.1	12.5	19.4	6,514	5,108	1,406
KH	Cambodia 2014	32,230	71.9	18.7	18.4	18.1	16.1	10.3	18.4	5,985	4,555	1,430
NP	Nepal 2011	22,776	78.0	23.1	19.4	16.8	14.4	12.6	13.7	3,848	3,275	573
NP	Nepal 2016	23,046	81.9	22.1	17.1	16.6	14.6	13.0	16.6	3,749	3,008	741
PH	Philippines 1993	26,738	63.7	21.4	18.9	17.6	14.7	13.4	14.0	6,144	5,549	595
PH	Philippines 1998	24,745	64.3	21.3	17.6	17.2	15.2	13.0	15.6	5,229	4,667	562
PH	Philippines 2003	24,282	66.2	19.6	17.9	16.5	15.9	13.5	16.6	4,787	4,288	499
TL	Timor Leste 2009	22,591	67.4	25.5	18.7	12.4	13.9	14.0	15.5	6,225	6,044	181
TL	Timor Leste 2016	21,001	63.0	25.3	17.9	16.6	12.9	10.4	16.9	4,680	4,521	159
