# Supplementary material for: Reported patterns of pregnancy termination from Demographic and Health Surveys
Source: PLoS One. 2019 Aug 19;14(8):e0221178. doi: 10.1371/journal.pone.0221178 (PMC6699730; doi:10.1371/journal.pone.0221178)
Supplement: S3 Table — (PDF) [file pone.0221178.s005.pdf]

					Contraceptive use				Union status				Age-group					
			Total		Using		Not using		In-union		Not-in-union		15-24		25-34		35-49	
Code	Survey	Cluster	P	T	P	T	P	T	P	T	P	T	P	T	P	T	P	T
**Africa**																		
AO	Angola 2015	1	8,880	6.7	38	23.7	8,842	6.6	6,507	6.4	2,374	7.3	4,240	6.6	3,324	5.6	1,316	9.6
BF	Burkina Faso 2010	1	10,029	5.0	65	13.8	9,965	4.9	9,687	4.8	342	9.6	4,171	5.4	4,248	4.2	1,610	6.1
BJ	Benin 2011	1	8,253	3.8	39	10.3	8,215	3.8	7,434	3.6	819	6.1	3,180	3.9	4,001	3.5	1,072	5.0
BU	Burundi 2010	1	5,428	7.3	95	7.4	5,333	7.3	5,157	7.3	271	7.4	2,104	7.3	2,272	5.2	1,052	11.8
BU	Burundi 2016	1	9,060	8.2	151	6.0	8,909	8.2	8,463	8.3	598	5.5	3,051	7.8	4,310	6.5	1,699	12.9
ET	Ethiopia 2005	1	7,078	4.4	62	24.2	7,016	4.2	6,935	4.3	143	7.7	2,941	4.1	3,013	3.7	1,124	6.8
ET	Ethiopia 2011	1	7,506	6.3	373	7.2	7,133	6.2	7,315	6.2	191	7.9	3,074	6.5	3,288	4.3	1,144	11.2
ET	Ethiopia 2016	1	7,006	5.3	70	14.3	6,936	5.2	6,851	5.2	155	10.3	2,692	4.2	3,219	4.9	1,095	8.9
GH	Ghana 2008	2	2,097	14.2	162	17.3	1,935	14.0	1,811	11.8	286	29.4	811	15.5	931	12.5	355	15.8
GH	Ghana 2014	2	4,390	18.2	201	29.9	4,190	17.6	3,671	14.7	719	36.2	1,474	20.6	2,072	15.8	844	19.7
KE	Kenya 1998	1	3,748	5.5	326	4.9	3,422	5.6	3,069	5.2	679	7.1	1,837	5.2	1,487	5.4	424	7.5
KE	Kenya 2003	1	4,034	5.6	324	6.8	3,710	5.5	3,419	5.7	616	5.0	1,950	5.8	1,602	4.3	482	8.7
KE	Kenya 2008	1	3,895	5.9	495	5.7	3,400	6.0	3,249	6.4	647	3.7	1,818	4.0	1,584	5.7	493	13.6
KM	Comoros 2012	1	2,205	7.6	26	3.8	2,180	7.6	2,103	7.5	102	8.8	796	5.2	1,037	7.3	372	13.4
LB	Liberia 2013	2	4,599	12.0	91	37.4	4,507	11.5	3,498	11.9	1,101	12.2	2,154	10.1	1,765	12.6	680	16.5
LS	Lesotho 2009	1	2,530	5.3	228	7.0	2,301	5.2	1,962	5.5	568	4.9	1,359	5.1	906	4.9	265	8.3
LS	Lesotho 2014	1	2,253	8.2	260	6.2	1,992	8.5	1,696	8.5	556	7.6	1,181	6.4	820	9.6	252	11.9
MA	Morocco 1992	1	3,445	8.5	435	11.3	3,010	8.1	3,430	8.5	15	6.7	1,034	7.5	1,660	8.3	751	10.4
MA	Morocco 2003	1	4,123	11.8	868	13.4	3,255	11.4	4,091	11.8	33	15.2	1,374	9.3	1,935	10.2	814	19.8
MD	Madagascar 2008	1	8,297	7.3	517	13.2	7,780	6.9	7,533	6.8	763	12.3	3,909	6.9	3,109	7.1	1,279	8.9
ML	Mali 2012	1	6,392	4.1	9	11.1	6,383	4.0	6,084	4.0	308	5.2	2,680	4.0	2,838	3.6	874	5.5
MW	Malawi 2004	1	7,235	4.9	240	3.8	6,995	5.0	6,651	4.9	584	5.1	3,932	4.8	2,474	4.4	829	7.0
MW	Malawi 2010	1	13,049	5.5	945	3.9	12,103	5.6	12,080	5.3	969	8.5	6,257	5.1	5,072	5.3	1,720	7.7
MW	Malawi 2015	1	11,077	5.7	225	6.2	10,853	5.6	9,652	5.4	1,426	7.4	5,612	5.8	4,167	5.1	1,298	6.9
MZ	Mozambique 2011	1	7,888	6.3	58	19.0	7,830	6.2	6,872	5.6	1,016	11.0	3,648	6.9	3,045	5.3	1,195	6.9
NG	Nigeria 2008	1	18,702	7.1	909	12.9	17,794	6.8	17,311	6.2	1,392	18.0	7,470	7.2	8,253	5.8	2,979	10.5

					Contraceptive use				Union status				Age-group					
			Total		Using		Not using		In-union		Not-in-union		15-24		25-34		35-49	
Code	Survey	Cluster	P	T	P	T	P	T	P	T	P	T	P	T	P	T	P	T
NG	Nigeria 2013	1	21,249	7.6	501	17.6	20,748	7.3	20,002	7.0	1,247	16.1	8,451	6.9	9,410	7.0	3,388	10.9
NI	Niger 2012	1	8,955	7.0	32	6.2	8,923	7.0	8,785	7.1	171	6.4	3,758	5.9	3,906	6.6	1,291	11.7
NM	Namibia 2006	1	3,385	5.3	309	4.2	3,076	5.4	1,705	7.1	1,680	3.5	1,486	3.6	1,385	5.3	514	10.5
NM	Namibia 2013	1	3,312	6.9	327	4.6	2,985	7.2	1,455	8.1	1,857	6.0	1,414	4.5	1,388	8.1	510	10.4
RW	Rwanda 2010	1	5,835	7.1	248	9.7	5,587	7.0	5,199	7.3	635	6.0	2,036	6.9	2,729	5.5	1,070	12.0
RW	Rwanda 2014	1	5,556	7.9	370	11.6	5,186	7.6	4,747	8.2	809	5.9	1,803	6.9	2,794	7.0	959	12.3
SL	Sierra Leone 2008	1	3,946	6.3	94	9.6	3,853	6.2	3,502	6.1	444	8.1	1,685	6.1	1,687	6.1	574	7.7
SL	Sierra Leone 2013	1	7,952	6.8	104	21.2	7,848	6.6	6,790	6.5	1,163	8.5	3,373	6.4	3,332	6.2	1,247	9.1
SN	Senegal 2012	1	4,419	9.3	33	12.1	4,386	9.3	4,209	9.4	209	6.7	1,773	9.4	1,886	7.9	760	12.6
SN	Senegal 2014	1	4,188	8.3	28	10.7	4,159	8.3	3,915	8.4	273	7.0	1,570	7.4	1,913	8.4	705	10.2
SN	Senegal 2015	1	4,294	9.1	55	14.5	4,239	9.1	4,066	9.3	229	5.7	1,567	7.0	1,947	7.6	780	17.1
SN	Senegal 2016	1	4,115	9.1	91	9.9	4,024	9.1	3,930	8.9	185	12.4	1,523	7.6	1,898	8.1	694	15.0
SN	Senegal 2017	1	7,728	10.3	63	6.3	7,665	10.4	7,326	10.5	402	7.7	2,754	8.8	3,558	8.7	1,416	17.4
TZ	Tanzania 2004	1	6,052	8.8	255	13.7	5,796	8.6	5,288	8.3	764	12.3	2,765	7.5	2,492	8.1	795	15.5
TZ	Tanzania 2010	1	5,535	8.1	309	4.2	5,226	8.3	4,938	8.3	597	6.0	2,392	7.3	2,251	6.8	892	13.5
TZ	Tanzania 2015	1	6,999	9.8	368	12.2	6,631	9.7	6,053	9.7	946	10.5	3,121	8.5	2,719	9.3	1,159	14.5
UG	Uganda 2006	1	5,778	9.7	328	11.6	5,450	9.6	5,291	9.5	487	12.3	2,586	8.9	2,332	7.7	860	17.4
UG	Uganda 2011	1	5,572	10.0	253	9.9	5,319	10.0	5,040	9.6	532	13.5	2,587	9.4	2,187	8.1	798	17.0
UG	Uganda 2016	2	10,528	11.0	422	17.3	10,106	10.7	9,152	10.8	1,376	11.8	5,025	9.9	4,064	9.6	1,439	18.4
ZM	Zambia 2007	1	4,384	6.2	468	6.4	3,917	6.2	3,828	6.1	556	7.0	1,957	6.0	1,814	6.2	613	6.9
ZM	Zambia 2013	1	8,592	5.6	536	5.0	8,056	5.7	7,181	5.6	1,411	6.0	3,819	5.3	3,522	4.8	1,251	9.0
ZW	Zimbabwe 1994	1	2,645	8.2	279	9.7	2,366	8.1	2,231	8.2	414	8.2	1,295	8.2	992	6.5	358	13.4
ZW	Zimbabwe 1999	1	2,452	8.2	222	8.6	2,230	8.1	2,027	8.2	425	8.2	1,349	8.2	808	6.6	295	12.2
ZW	Zimbabwe 2005	1	3,557	7.3	358	6.4	3,199	7.4	3,028	7.4	529	6.6	1,940	6.9	1,274	6.0	343	14.3
ZW	Zimbabwe 2010	1	3,981	7.0	283	6.7	3,698	7.0	3,404	7.1	578	6.6	1,987	6.2	1,599	7.8	395	7.6
ZW	Zimbabwe 2015	1	4,207	8.5	346	7.2	3,860	8.6	3,634	8.4	572	9.1	1,879	9.0	1,805	6.2	523	14.3
**Central and West Asia & Europe**																		

					Contraceptive use				Union status				Age-group					
			Total		Using		Not using		In-union		Not-in-union		15-24		25-34		35-49	
Code	Survey	Cluster	P	T	P	T	P	T	P	T	P	T	P	T	P	T	P	T
AL	Albania 2008	2	1,049	15.9	221	18.6	828	15.3	996	16.6	53	3.8	372	10.2	580	15.3	97	41.2
AL	Albania 2017	1	1,767	9.2	82	22.0	1,686	8.6	1,665	9.6	103	2.9	591	7.3	994	7.8	182	23.1
AM	Armenia 2000	4	2,508	62.8	1,080	85.2	1,428	45.9	2,495	62.8	13	61.5	988	40.1	1,096	74.0	424	87.0
AM	Armenia 2005	4	2,035	51.9	586	83.3	1,449	39.3	1,991	51.9	43	53.5	830	31.1	960	62.7	245	80.4
AM	Armenia 2010	3	1,508	36.6	276	67.4	1,232	29.7	1,487	36.9	20	15.0	690	22.8	677	46.1	141	58.9
AM	Armenia 2015	3	1,549	32.3	220	75.5	1,328	25.3	1,522	32.4	27	29.6	571	21.9	838	36.8	140	48.6
AZ	Azerbaijan 2006	4	3,121	52.2	664	82.4	2,457	44.1	3,069	52.6	52	32.7	1,234	31.4	1,382	60.6	505	80.4
KK	Kazakhstan 1999	4	1,613	46.9	374	78.3	1,238	37.6	1,458	45.5	154	61.0	653	35.2	746	51.3	214	67.3
KY	Kyrgyz Rep. 2012	3	3,436	22.4	213	50.2	3,222	20.6	3,317	22.4	119	23.5	1,458	17.6	1,543	24.4	435	31.7
MB	Moldova 2005	4	1,854	44.1	536	67.9	1,318	34.4	1,713	43.3	141	53.2	869	35.4	790	46.7	195	72.3
TJ	Tajikistan 2012	3	4,111	16.0	74	54.1	4,037	15.3	4,034	15.9	77	16.9	2,003	11.2	1,707	17.6	401	32.4
TJ	Tajikistan 2017	2	4,850	15.9	41	39.0	4,809	15.7	4,771	15.9	80	13.8	2,462	10.5	2,027	18.7	361	36.8
TR	Turkey 1998	3	2,860	24.5	615	45.7	2,244	18.8	2,849	24.5	10	30.0	1,316	17.6	1,233	25.9	311	48.9
TR	Turkey 2003	3	3,200	23.0	851	37.6	2,350	17.7	3,199	22.9	2	100.0	1,393	15.7	1,398	23.8	409	45.0
UA	Ukraine 2007	3	1,061	33.9	264	65.2	797	23.6	974	34.5	87	27.6	447	20.4	502	41.0	112	56.2
**Latin America**																		
BO	Bolivia 1994	1	4,086	9.0	776	13.8	3,310	7.9	3,651	9.2	435	7.6	1,625	6.9	1,806	10.4	655	10.5
BO	Bolivia 2008	1	6,217	12.9	1,522	15.5	4,695	12.1	5,150	13.1	1,067	12.0	2,618	10.6	2,578	13.3	1,021	18.1
BR	Brazil 1996	2	3,386	13.6	697	16.8	2,689	12.7	2,699	13.0	687	15.7	1,658	12.5	1,326	11.7	402	24.1
CO	Colombia 1990	2	2,684	12.5	521	17.1	2,163	11.4	2,312	13.1	372	8.6	1,344	10.5	1,079	13.1	261	20.7
CO	Colombia 1995	1	3,543	11.3	965	14.5	2,578	10.1	2,867	11.6	675	9.9	1,751	10.0	1,420	11.7	372	15.9
CO	Colombia 2000	2	3,350	15.7	1,119	16.9	2,230	15.2	2,489	15.2	861	17.1	1,613	13.3	1,353	15.7	384	26.0
CO	Colombia 2005	2	10,185	17.8	2,937	21.5	7,248	16.3	7,425	16.7	2,760	20.7	5,200	16.2	3,746	17.4	1,239	25.8
CO	Colombia 2010	2	11,639	17.8	2,543	21.0	9,096	16.9	8,714	18.2	2,925	16.7	5,995	15.8	4,303	17.8	1,341	26.5
CO	Colombia 2015	2	7,807	15.4	1,582	19.3	6,224	14.4	5,908	15.3	1,899	15.7	3,913	13.2	3,099	16.5	795	22.3
DR	Dominican Rep. 1991	2	2,877	14.4	327	21.7	2,549	13.5	2,722	14.4	155	14.2	1,534	10.7	1,145	17.9	198	22.7
DR	Dominican Rep. 1996	2	3,255	16.8	398	19.1	2,857	16.5	2,933	15.9	322	25.2	1,818	15.4	1,234	17.2	203	26.6

Code	Survey	Cluster	Total		Contraceptive use				Union status				Age-group					
					Using		Not using		In-union		Not-in-union		15-24		25-34		35-49	
			P	T	P	T	P	T	P	T	P	T	P	T	P	T	P	T
DR	Dominican Rep. 1999	2	435	21.8	60	20.0	375	22.1	394	18.5	41	53.7	224	18.3	181	24.9	30	30.0
DR	Dominican Rep. 2002	2	8,065	16.2	1,044	21.6	7,021	15.4	7,094	14.6	971	27.9	4,557	15.3	2,969	16.3	539	22.4
GU	Guatemala 1995	1	6,179	6.0	245	11.0	5,934	5.7	5,845	6.0	334	4.8	2,952	5.1	2,355	5.6	872	9.7
GU	Guatemala 1998	1	2,988	5.9	197	11.2	2,791	5.4	2,736	5.7	252	7.1	1,451	4.5	1,125	6.7	412	8.5
GU	Guatemala 2014	1	8,300	7.8	935	10.8	7,365	7.5	7,313	8.0	987	6.8	4,193	6.3	3,179	8.2	928	13.7
GY	Guyana 2009	2	1,567	21.8	195	34.9	1,372	20.0	1,254	22.2	313	20.1	768	15.5	583	25.2	216	35.2
HN	Honduras 2005	1	6,767	9.1	1,053	12.5	5,713	8.4	6,241	9.0	526	10.1	3,417	7.1	2,545	8.8	805	18.1
HN	Honduras 2011	1	7,120	9.8	757	14.3	6,363	9.3	6,281	10.2	838	6.8	3,709	8.1	2,658	9.8	753	18.5
NC	Nicaragua 1998	1	5,145	8.0	469	12.4	4,677	7.5	4,860	7.8	285	10.9	2,781	7.3	1,828	8.2	536	10.8
PE	Peru 1991	1	5,696	10.2	1,643	13.1	4,053	9.0	4,993	10.2	703	10.4	2,342	6.8	2,473	11.0	881	17.0
PE	Peru 1996	1	10,459	10.0	3,037	12.4	7,422	9.1	9,026	10.0	1,433	10.1	4,453	7.9	4,347	10.6	1,659	14.4
PE	Peru 2000	1	8,027	10.3	1,976	13.9	6,052	9.1	6,565	10.0	1,462	11.5	3,310	7.8	3,406	10.8	1,311	15.1
PE	Peru 2004	1	4,531	11.3	1,249	14.7	3,282	10.0	3,667	11.0	864	12.6	1,813	9.7	1,917	10.4	801	17.0
PE	Peru 2007	2	5,949	14.0	1,810	17.6	4,139	12.4	4,762	14.0	1,187	14.1	2,385	12.0	2,548	13.1	1,016	20.9
PE	Peru 2009	2	6,514	14.0	2,026	17.8	4,488	12.3	5,239	12.9	1,275	18.6	2,513	12.8	2,839	12.8	1,162	19.8
PE	Peru 2010	2	6,115	15.8	1,906	21.2	4,209	13.3	4,930	14.8	1,185	19.8	2,419	12.6	2,606	15.5	1,090	23.4
PE	Peru 2011	2	6,109	15.1	1,963	20.2	4,146	12.7	4,905	14.0	1,204	19.8	2,297	13.3	2,709	14.2	1,103	21.3
PY	Paraguay 1990	1	2,789	10.9	414	19.8	2,375	9.3	2,453	11.3	336	8.3	1,088	8.4	1,212	11.1	489	16.0
**South and Southeast Asia**																		
IA	India 2005	2	38,223	12.2	1,591	27.9	36,632	11.5	38,134	12.1	89	22.5	23,470	11.0	13,046	13.5	1,707	18.1
ID	Indonesia 2012	1	11,858	10.6	843	11.6	11,016	10.5	11,369	10.8	489	6.1	4,052	8.3	5,788	9.9	2,018	17.3
KH	Cambodia 2010	3	6,514	21.6	409	49.1	6,105	19.7	6,359	21.7	155	17.4	2,545	15.1	2,859	20.4	1,110	39.5
KH	Cambodia 2014	3	5,985	23.9	584	53.3	5,401	20.7	5,863	23.9	122	24.6	2,401	17.2	2,860	23.7	724	47.0
NP	Nepal 2011	2	3,848	14.9	191	40.3	3,657	13.6	3,807	14.9	41	14.6	2,158	10.9	1,401	19.2	289	23.9
NP	Nepal 2016	3	3,749	19.8	187	41.7	3,563	18.6	3,713	19.9	36	5.6	2,196	14.9	1,358	24.0	195	44.6
PH	Philippines 1993	1	6,144	9.7	749	12.1	5,395	9.3	5,842	9.8	302	7.3	2,143	8.0	2,939	8.5	1,062	16.2
PH	Philippines 1998	1	5,229	10.7	1,007	11.4	4,221	10.6	4,910	10.9	319	8.2	1,770	9.1	2,544	9.2	915	18.1

					Contraceptive use				Union status				Age-group					
			Total		Using		Not using		In-union		Not-in-union		15-24		25-34		35-49	
Code	Survey	Cluster	P	T	P	T	P	T	P	T	P	T	P	T	P	T	P	T
PH	Philippines 2003	1	4,787	10.4	655	10.8	4,133	10.4	4,414	10.8	373	5.6	1,747	8.7	2,183	9.2	857	17.0
TL	Timor Leste 2009	1	6,225	2.9	31	6.5	6,194	2.9	6,109	2.8	117	6.8	2,041	2.8	2,728	2.5	1,456	3.7
TL	Timor Leste 2016	1	4,680	3.4	16	0.0	4,664	3.4	4,449	3.4	231	2.6	1,616	4.0	2,340	2.7	724	4.3
