# Supplementary material for: Reported patterns of pregnancy termination from Demographic and Health Surveys
Source: PLoS One. 2019 Aug 19;14(8):e0221178. doi: 10.1371/journal.pone.0221178 (PMC6699730; doi:10.1371/journal.pone.0221178)
Supplement: S4 Table — (PDF) [file pone.0221178.s006.pdf]

	15-19	20-24	25-29	30-34	35-39	40-44	45-49
**Africa**							
**Angola 2015**							
Using	0.0	36.8	0.0	0.0	0.0		
Not using	6.7	6.2	5.3	6.3	7.3	15.7	15.7
All	6.6	6.5	5.2	6.3	7.3	15.7	15.7
ASTR	12.0	18.0	15.0	16.0	15.0	17.0	4.0
**Burkina Faso 2010**							
Using	2.6	22.8	6.1	18.5	20.1		
Not using	7.2	4.3	3.7	4.7	4.7	9.0	9.0
All	7.2	4.4	3.7	4.8	4.8	9.0	9.0
ASTR	10.0	12.0	10.0	12.0	10.0	9.0	2.0
**Benin 2011**							
Using	0.0	9.8	0.0	23.3	0.0	60.3	60.3
Not using	4.5	3.6	3.1	4.0	5.5	3.4	3.4
All	4.5	3.6	3.1	4.1	5.4	4.0	4.0
ASTR	4.0	9.0	8.0	9.0	7.0	3.0	1.0
**Burundi 2010**							
Using	0.0	11.1	0.0	2.8	0.0	42.2	42.2
Not using	10.8	6.1	4.4	6.9	8.8	18.1	18.1
All	10.8	6.1	4.3	6.8	8.6	19.0	19.0
ASTR	8.0	18.0	14.0	20.0	21.0	24.0	7.0
**Burundi 2016**							
Using	0.0	4.9	0.0	6.4	13.0	15.6	15.6
Not using	8.6	7.6	6.5	6.7	10.8	16.9	16.9
All	8.6	7.6	6.4	6.7	10.9	16.8	16.8
ASTR	5.0	18.0	18.0	18.0	23.0	20.0	4.0
**Ethiopia 2005**							
Using	6.5	28.9	15.9	17.5	21.9	81.3	81.3
Not using	3.2	4.4	3.2	4.1	4.9	9.1	9.1
All	3.2	4.7	3.3	4.1	5.2	9.6	9.6
ASTR	3.0	11.0	8.0	10.0	9.0	9.0	4.0
**Ethiopia 2011**							
Using	2.9	6.3	6.2	9.1	3.8	59.3	59.3
Not using	6.5	6.5	3.3	5.6	8.8	15.9	15.9
All	6.4	6.5	3.4	5.7	8.6	16.9	16.9
ASTR	5.0	14.0	8.0	12.0	14.0	14.0	6.0
**Ethiopia 2016**							
Using	41.4	3.8	18.7	12.1	4.1		
Not using	5.0	3.7	4.5	5.3	6.7	14.8	14.8
All	5.3	3.7	4.7	5.4	6.7	14.8	14.8
ASTR	4.0	8.0	10.0	11.0	10.0	12.0	4.0
**Ghana 2008**							
Using	20.0	20.8	20.0	9.4	19.0	0.0	0.0
Not using	17.1	14.2	9.9	15.0	13.8	20.7	20.7
All	17.3	14.7	10.8	14.6	14.3	19.7	19.7
ASTR	14.0	30.0	25.0	30.0	20.0	14.0	2.0

	15-19	20-24	25-29	30-34	35-39	40-44	45-49
**Ghana 2014**							
Using	10.3	46.4	40.5	15.8	21.6	23.7	23.7
Not using	19.5	20.1	14.5	15.9	18.4	22.7	22.7
All	19.2	21.3	15.7	15.9	18.5	22.8	22.8
ASTR	18.0	44.0	37.0	37.0	31.0	15.0	5.0
**Kenya 1998**							
Using	9.2	0.9	4.9	2.9	26.0	0.0	0.0
Not using	6.0	5.0	5.7	5.5	5.3	10.5	10.5
All	6.2	4.6	5.6	5.2	6.6	9.5	9.5
ASTR	7.0	12.0	13.0	10.0	8.0	5.0	2.0
**Kenya 2003**							
Using	12.1	6.9	2.5	5.0	10.6	4.0	4.0
Not using	6.2	5.3	3.7	5.4	8.2	9.7	9.7
All	6.6	5.4	3.6	5.4	8.5	9.3	9.3
ASTR	8.0	14.0	9.0	11.0	11.0	6.0	2.0
**Kenya 2008**							
Using	3.2	7.0	5.5	4.1	4.2	15.7	15.7
Not using	3.1	4.2	6.3	5.2	12.4	18.8	18.8
All	3.1	4.5	6.2	5.0	11.3	18.4	18.4
ASTR	3.0	11.0	14.0	9.0	15.0	11.0	3.0
**Comoros 2012**							
Using	0.0	0.0	2.8	0.0	0.0	100.0	100.0
Not using	5.3	5.1	7.9	6.6	12.0	18.1	18.1
All	5.3	5.0	7.9	6.4	11.9	18.8	18.8
ASTR	4.0	9.0	17.0	14.0	18.0	15.0	7.0
**Liberia 2013**							
Using	29.6	37.5	38.9	56.4	36.4	45.1	45.1
Not using	8.2	10.8	12.2	11.7	11.8	27.0	27.0
All	8.7	11.2	12.9	12.2	12.6	27.4	27.4
ASTR	14.0	28.0	30.0	25.0	19.0	19.0	5.0
**Lesotho 2009**							
Using	4.4	8.6	2.8	6.2	10.5	29.7	29.7
Not using	4.4	5.2	4.3	5.9	10.0	4.0	4.0
All	4.4	5.5	4.1	5.9	10.1	5.6	5.6
ASTR	4.0	10.0	7.0	7.0	8.0	2.0	0.0
**Lesotho 2014**							
Using	13.9	1.3	3.1	11.3	6.5	6.2	6.2
Not using	6.3	6.8	9.8	10.5	12.7	12.8	12.8
All	7.0	6.2	9.0	10.6	11.6	12.0	12.0
ASTR	7.0	12.0	14.0	13.0	9.0	7.0	1.0
**Morocco 1992**							
Using	11.1	4.6	9.0	9.4	13.4	25.5	25.5
Not using	9.0	7.2	7.6	8.6	8.9	8.5	8.5
All	9.1	7.0	7.8	8.7	9.7	11.7	11.7
ASTR	4.0	10.0	15.0	17.0	15.0	11.0	5.0
**Morocco 2003**							
Using	18.1	11.1	10.2	12.3	15.8	21.8	21.8

	15-19	20-24	25-29	30-34	35-39	40-44	45-49
Not using	10.1	8.5	8.4	11.7	16.9	29.8	29.8
All	10.7	8.9	8.8	11.8	16.6	27.2	27.2
ASTR	4.0	10.0	12.0	17.0	15.0	10.0	2.0
**Madagascar 2008**							
Using	18.9	9.2	14.2	12.4	10.8	21.1	21.1
Not using	7.0	6.2	6.5	6.8	7.5	11.4	11.4
All	7.5	6.4	7.1	7.2	7.8	11.8	11.8
ASTR	12.0	16.0	16.0	13.0	11.0	8.0	2.0
**Mali 2012**							
Using		0.0	11.5	0.0	0.0		
Not using	4.4	3.7	3.8	3.4	4.6	7.2	7.2
All	4.4	3.7	3.8	3.4	4.6	7.2	7.2
ASTR	8.0	10.0	11.0	8.0	8.0	7.0	3.0
**Malawi 2004**							
Using	14.9	0.6	0.0	7.5	6.0	5.1	5.1
Not using	5.8	4.3	4.1	5.0	7.2	6.9	6.9
All	5.9	4.2	4.0	5.1	7.1	6.7	6.7
ASTR	10.0	13.0	11.0	12.0	12.0	6.0	3.0
**Malawi 2010**							
Using	5.3	2.4	3.1	5.8	5.5	4.5	4.5
Not using	7.3	3.9	5.5	5.4	5.3	13.1	13.1
All	7.3	3.7	5.3	5.4	5.3	12.6	12.6
ASTR	12.0	10.0	13.0	12.0	9.0	12.0	5.0
**Malawi 2015**							
Using	7.8	2.5	8.0	10.0	3.3	0.0	0.0
Not using	6.5	5.4	5.4	4.3	5.7	10.0	10.0
All	6.5	5.3	5.4	4.5	5.7	9.9	9.9
ASTR	9.0	12.0	11.0	7.0	7.0	6.0	2.0
**Mozambique 2011**							
Using	20.4	41.1	3.8	0.0	17.7	19.5	19.5
Not using	8.4	5.4	5.8	4.5	6.1	8.6	8.6
All	8.5	5.7	5.8	4.5	6.1	8.7	8.7
ASTR	16.0	16.0	16.0	10.0	11.0	8.0	3.0
**Nigeria 2008**							
Using	28.5	17.4	6.9	9.5	14.8	10.0	10.0
Not using	6.8	6.6	5.1	6.5	9.9	11.3	11.3
All	7.5	7.1	5.2	6.7	10.2	11.3	11.3
ASTR	10.0	17.0	15.0	17.0	18.0	11.0	6.0
**Nigeria 2013**							
Using	39.5	21.5	12.0	8.0	26.7	20.8	20.8
Not using	7.1	6.3	6.6	7.3	9.3	13.0	13.0
All	7.5	6.5	6.7	7.3	9.8	13.2	13.2
ASTR	10.0	16.0	18.0	19.0	17.0	12.0	4.0
**Niger 2012**							
Using	22.0	0.0	8.5	0.0	0.0		
Not using	9.0	3.8	5.8	7.9	9.5	17.3	17.3
All	9.0	3.8	5.8	7.9	9.4	17.3	17.3

	15-19	20-24	25-29	30-34	35-39	40-44	45-49
ASTR	20.0	13.0	20.0	24.0	23.0	21.0	10.0
**Namibia 2006**							
Using	3.4	1.4	5.1	4.3	4.6	27.6	27.6
Not using	3.1	4.1	4.4	6.4	8.6	14.5	14.5
All	3.2	3.9	4.5	6.2	8.4	15.3	15.3
ASTR	3.0	7.0	7.0	10.0	10.0	8.0	1.0
**Namibia 2013**							
Using	2.7	2.5	7.5	5.5	6.8	6.7	6.7
Not using	3.5	5.4	9.3	7.0	7.8	17.9	17.9
All	3.4	5.1	9.1	6.8	7.7	17.0	17.0
ASTR	3.0	9.0	17.0	11.0	9.0	9.0	2.0
**Rwanda 2010**							
Using	52.3	4.0	3.2	11.8	12.4	17.7	17.7
Not using	4.5	7.4	5.5	5.2	8.1	17.7	17.7
All	4.8	7.4	5.4	5.6	8.4	17.7	17.7
ASTR	2.0	15.0	13.0	12.0	14.0	19.0	4.0
**Rwanda 2014**							
Using	0.0	3.7	8.6	11.1	17.5	17.1	17.1
Not using	6.4	7.2	5.9	7.9	9.9	16.0	16.0
All	6.3	7.1	6.0	8.2	10.9	16.1	16.1
ASTR	3.0	14.0	14.0	17.0	16.0	13.0	2.0
**Sierra Leone 2008**							
Using	47.1	6.4	8.2	0.0	0.0	100.0	100.0
Not using	6.1	5.8	6.2	6.1	6.3	10.1	10.1
All	6.5	5.8	6.2	6.0	6.2	11.2	11.2
ASTR	10.0	14.0	14.0	12.0	10.0	9.0	5.0
**Sierra Leone 2013**							
Using	21.5	12.0	13.3	52.5	11.2	59.7	59.7
Not using	6.8	5.9	6.1	5.8	8.2	10.7	10.7
All	7.0	6.0	6.2	6.2	8.3	11.4	11.4
ASTR	9.0	14.0	15.0	12.0	13.0	8.0	4.0
**Senegal 2012**							
Using		7.7	0.0	11.4	0.0	41.0	41.0
Not using	11.5	8.3	8.2	7.3	10.4	17.2	17.2
All	11.5	8.3	8.2	7.4	10.3	17.6	17.6
ASTR	10.0	19.0	22.0	18.0	21.0	21.0	4.0
**Senegal 2014**							
Using		0.0	19.2	0.0	0.0	0.0	0.0
Not using	6.8	7.7	6.5	11.0	10.5	10.3	10.3
All	6.8	7.6	6.6	11.0	10.5	10.2	10.2
ASTR	7.0	16.0	17.0	26.0	18.0	11.0	2.0
**Senegal 2015**							
Using	0.0	0.0	48.0	0.0	14.6	23.1	23.1
Not using	8.8	6.2	5.9	9.7	15.5	20.8	20.8
All	8.7	6.1	6.3	9.5	15.5	20.8	20.8
ASTR	8.0	12.0	15.0	22.0	30.0	22.0	4.0
**Senegal 2016**							

	15-19	20-24	25-29	30-34	35-39	40-44	45-49
Using		4.7	0.0	16.6	6.6	36.6	36.6
Not using	7.0	7.9	6.2	10.6	14.1	17.5	17.5
All	7.0	7.9	6.1	10.8	13.7	17.9	17.9
ASTR	5.0	16.0	15.0	24.0	23.0	17.0	5.0
**Senegal 2017**							
Using	0.0	0.0	7.5	4.3	10.7	0.0	0.0
Not using	9.9	8.1	8.0	9.7	15.7	21.1	21.1
All	9.9	8.1	8.0	9.6	15.6	20.8	20.8
ASTR	9.0	16.0	19.0	22.0	27.0	21.0	6.0
**Tanzania 2004**							
Using	0.0	11.1	18.3	12.9	7.6	31.2	31.2
Not using	9.4	6.2	7.1	8.5	11.1	23.2	23.2
All	9.3	6.3	7.8	8.7	10.9	23.5	23.5
ASTR	14.0	19.0	21.0	21.0	19.0	24.0	6.0
**Tanzania 2010**							
Using	0.0	3.3	3.2	5.8	7.5	4.4	4.4
Not using	7.0	7.8	6.8	7.1	12.8	17.1	17.1
All	6.8	7.6	6.6	7.0	12.5	15.9	15.9
ASTR	9.0	21.0	18.0	16.0	23.0	14.0	4.0
**Tanzania 2015**							
Using	23.5	5.9	13.9	11.5	10.2	15.5	15.5
Not using	8.8	8.1	9.3	8.6	12.0	20.1	20.1
All	9.2	8.0	9.6	8.8	11.9	19.7	19.7
ASTR	13.0	21.0	25.0	19.0	20.0	18.0	4.0
**Uganda 2006**							
Using	14.0	8.9	8.7	7.7	25.1	29.2	29.2
Not using	11.5	7.1	6.5	9.3	13.9	23.5	23.5
All	11.6	7.2	6.6	9.2	14.6	23.6	23.6
ASTR	20.0	24.0	22.0	26.0	33.0	29.0	8.0
**Uganda 2011**							
Using	3.3	10.9	5.7	4.4	18.2	55.6	55.6
Not using	13.0	7.3	8.2	8.6	12.9	25.3	25.3
All	12.7	7.5	8.1	8.3	13.1	26.6	26.6
ASTR	19.0	25.0	25.0	21.0	26.0	27.0	8.0
**Uganda 2016**							
Using	12.9	18.4	13.6	17.2	27.3	17.7	17.7
Not using	10.4	9.3	8.3	10.9	14.9	25.7	25.7
All	10.4	9.6	8.6	11.2	15.5	25.2	25.2
ASTR	15.0	28.0	23.0	26.0	27.0	23.0	5.0
**Zambia 2007**							
Using	11.3	6.1	4.9	10.9	2.3	0.0	0.0
Not using	6.8	5.3	6.5	5.3	6.0	11.2	11.2
All	7.0	5.4	6.3	6.0	5.5	10.1	10.1
ASTR	11.0	16.0	18.0	15.0	11.0	10.0	3.0
**Zambia 2013**							
Using	3.2	2.6	3.4	2.6	14.6	3.0	3.0
Not using	5.6	5.3	4.6	5.4	6.8	13.9	13.9

	15-19	20-24	25-29	30-34	35-39	40-44	45-49
All	5.6	5.2	4.5	5.2	7.6	12.7	12.7
ASTR	8.0	13.0	11.0	11.0	12.0	10.0	2.0
**Zimbabwe 1994**							
Using	4.4	6.0	10.2	9.6	14.2	22.8	22.8
Not using	8.2	8.6	5.6	6.4	7.8	23.7	23.7
All	7.9	8.3	6.1	6.7	8.9	23.5	23.5
ASTR	9.0	19.0	13.0	12.0	11.0	16.0	4.0
**Zimbabwe 1999**							
Using	13.9	15.0	5.0	0.0	4.1	12.7	12.7
Not using	8.6	7.1	7.0	6.8	11.2	18.0	18.0
All	8.8	7.9	6.8	5.9	10.4	17.5	17.5
ASTR	11.0	17.0	13.0	9.0	13.0	10.0	3.0
**Zimbabwe 2005**							
Using	26.3	2.2	4.9	2.6	6.7	22.6	22.6
Not using	8.4	5.8	7.5	4.3	12.4	18.5	18.5
All	9.3	5.4	7.2	4.1	11.8	19.0	19.0
ASTR	10.0	12.0	13.0	6.0	12.0	10.0	3.0
**Zimbabwe 2010**							
Using	24.2	5.4	3.2	11.2	5.8	0.0	0.0
Not using	7.8	5.2	7.5	8.5	7.9	7.6	7.6
All	8.0	5.2	7.2	8.8	7.7	6.5	6.5
ASTR	10.0	12.0	15.0	14.0	9.0	2.0	1.0
**Zimbabwe 2015**							
Using	0.0	4.1	8.3	9.7	11.8	10.0	10.0
Not using	9.9	9.1	4.9	7.3	11.0	26.6	26.6
All	9.6	8.6	5.2	7.6	11.1	24.7	24.7
ASTR	12.0	19.0	11.0	12.0	13.0	11.0	2.0
**Central and West Asia & Europe**							
**Albania 2008**							
Using	0.0	22.9	14.6	19.9	23.9		
Not using	12.0	7.0	10.4	22.2	38.4	71.3	71.3
All	11.2	9.9	11.4	21.5	35.8	71.3	71.3
ASTR	2.0	10.0	16.0	18.0	8.0	2.0	0.0
**Albania 2017**							
Using	0.0	19.4	16.3	24.9	39.9	72.2	72.2
Not using	5.8	7.0	7.0	7.4	19.5	38.9	38.9
All	5.6	7.6	7.5	8.3	20.2	41.2	41.2
ASTR	1.0	7.0	10.0	8.0	7.0	2.0	0.0
**Armenia 2000**							
Using	53.4	75.7	86.0	88.6	93.7	97.1	97.1
Not using	19.1	30.7	50.3	73.0	74.6	86.7	86.7
All	23.1	45.3	68.8	81.3	85.3	91.9	91.9
ASTR	15.0	123.0	194.0	152.0	93.0	34.0	0.0
**Armenia 2005**							
Using	44.7	67.9	86.0	96.4	89.3	74.3	74.3
Not using	19.4	24.1	44.2	59.1	72.3	85.0	85.0
All	20.4	33.2	57.8	73.7	79.8	80.8	80.8

	15-19	20-24	25-29	30-34	35-39	40-44	45-49
ASTR	8.0	74.0	147.0	104.0	63.0	17.0	
**Armenia 2010**							
Using	6.1	49.1	69.2	72.7	75.5	89.1	89.1
Not using	20.9	20.4	32.0	50.2	47.1	54.6	54.6
All	20.7	23.3	40.7	57.2	54.4	68.8	68.8
ASTR	7.0	42.0	70.0	56.0	29.0	11.0	
**Armenia 2015**							
Using	75.2	56.0	76.7	78.3	95.5	91.8	91.8
Not using	14.9	19.1	27.7	31.8	29.8	53.5	53.5
All	16.1	22.8	34.8	40.9	43.8	68.1	68.1
ASTR	5.0	37.0	62.0	38.0	19.0	9.0	2.0
**Azerbaijan 2006**							
Using	94.7	64.9	78.8	88.6	86.7	100.0	100.0
Not using	20.2	29.2	49.0	56.7	72.6	84.1	84.1
All	22.6	33.1	55.8	67.3	77.5	89.2	89.2
ASTR	10.0	84.0	142.0	124.0	86.0	33.0	
**Kazakhstan 1999**							
Using	66.9	58.1	80.9	86.1	93.2	85.2	85.2
Not using	29.2	31.1	40.1	38.1	55.2	59.6	59.6
All	34.8	35.4	51.2	51.9	66.3	68.9	68.9
ASTR	21.0	91.0	111.0	69.0	47.0	20.0	
**Kyrgyz Rep. 2012**							
Using	17.0	48.4	51.9	52.6	48.0	81.5	81.5
Not using	9.5	17.8	22.4	21.7	29.8	30.0	30.0
All	9.8	19.2	24.5	24.3	31.4	31.9	31.9
ASTR	5.0	51.0	67.0	48.0	37.0	13.0	0.0
**Moldova 2005**							
Using	56.8	59.4	72.1	72.5	71.5	90.0	90.0
Not using	27.6	28.9	33.3	35.6	67.2	75.4	75.4
All	33.3	36.2	45.2	49.0	69.0	80.6	80.6
ASTR	17.0	75.0	78.0	55.0	38.0	12.0	
**Tajikistan 2012**							
Using		26.0	52.0	67.3	65.6	70.2	70.2
Not using	13.0	10.6	15.0	19.5	27.9	41.1	41.1
All	13.0	10.7	16.0	20.8	29.4	42.7	42.7
ASTR	8.0	30.0	41.0	36.0	29.0	14.0	1.0
**Tajikistan 2017**							
Using		21.7	41.0	78.4	40.3	100.0	100.0
Not using	10.2	10.5	16.1	22.8	32.4	51.7	51.7
All	10.2	10.6	16.4	23.2	32.6	51.8	51.8
ASTR	6.0	36.0	41.0	37.0	27.0	12.0	0.0
**Turkey 1998**							
Using	48.0	33.9	32.7	50.0	71.3	77.5	77.5
Not using	13.5	14.4	17.7	26.8	29.4	48.3	48.3
All	17.4	17.7	21.0	33.0	44.1	61.4	61.4
ASTR	13.0	35.0	40.0	46.0	33.0	21.0	2.0
**Turkey 2003**							

	15-19	20-24	25-29	30-34	35-39	40-44	45-49
Using	18.6	21.8	29.3	50.6	57.5	52.0	52.0
Not using	17.8	13.3	16.9	18.4	36.7	30.3	30.3
All	17.9	15.0	20.3	30.1	46.0	41.0	41.0
ASTR	10.0	24.0	34.0	34.0	32.0	8.0	1.0
**Ukraine 2007**							
Using	52.3	41.0	75.4	71.5	77.4	85.6	85.6
Not using	12.9	15.6	28.5	30.0	36.5	53.8	53.8
All	19.4	20.5	40.6	41.6	53.2	68.2	68.2
ASTR	6.0	24.0	42.0	27.0	15.0	6.0	0.0
**Latin America**							
**Bolivia 1994**							
Using	14.6	8.5	14.0	17.0	13.7	19.8	19.8
Not using	6.6	6.1	8.1	10.1	10.5	6.4	6.4
All	7.7	6.5	9.2	11.8	11.1	9.4	9.4
ASTR	8.0	16.0	23.0	25.0	17.0	7.0	2.0
**Bolivia 2008**							
Using	8.3	15.0	15.7	16.1	18.8	24.3	24.3
Not using	10.3	9.8	11.2	14.4	16.8	18.0	18.0
All	9.9	11.0	12.3	14.9	17.4	19.4	19.4
ASTR	10.0	22.0	24.0	22.0	20.0	10.0	2.0
**Brazil 1996**							
Using	19.7	20.6	11.6	11.0	23.2	30.6	30.6
Not using	11.2	10.6	12.2	11.2	21.0	31.7	31.7
All	12.3	12.6	12.1	11.1	21.5	31.4	31.4
ASTR	12.0	22.0	17.0	10.0	13.0	7.0	1.0
**Colombia 1990**							
Using	20.9	15.0	14.8	24.7	12.1	26.2	26.2
Not using	9.9	9.3	10.8	13.4	19.0	35.1	35.1
All	11.1	10.2	11.7	15.9	16.9	32.4	32.4
ASTR	9.0	19.0	20.0	18.0	13.0	9.0	1.0
**Colombia 1995**							
Using	13.3	13.8	15.0	14.4	12.0	28.1	28.1
Not using	8.1	9.3	10.0	11.1	11.7	23.8	23.8
All	9.1	10.4	11.5	12.1	11.8	25.3	25.3
ASTR	9.0	20.0	19.0	14.0	7.0	8.0	1.0
**Colombia 2000**							
Using	13.1	16.4	13.0	18.6	26.4	28.5	28.5
Not using	10.8	13.7	15.6	16.2	24.6	27.0	27.0
All	11.5	14.6	14.7	17.0	25.3	27.6	27.6
ASTR	11.0	24.0	22.0	20.0	17.0	6.0	1.0
**Colombia 2005**							
Using	17.1	22.4	17.9	22.1	33.9	24.0	24.0
Not using	14.7	14.7	15.8	17.3	21.0	30.4	30.4
All	15.3	16.8	16.4	18.8	25.1	28.2	28.2
ASTR	16.0	27.0	23.0	18.0	15.0	6.0	1.0
**Colombia 2010**							
Using	15.4	22.6	18.1	26.5	22.1	32.9	32.9

	15-19	20-24	25-29	30-34	35-39	40-44	45-49
Not using	12.8	16.4	15.8	18.3	23.8	34.6	34.6
All	13.4	17.7	16.3	20.1	23.4	34.2	34.2
ASTR	13.0	26.0	20.0	18.0	12.0	6.0	1.0
**Colombia 2015**							
Using	17.7	16.5	23.8	20.6	19.4	16.6	16.6
Not using	10.0	13.7	16.3	13.3	19.5	36.2	36.2
All	11.6	14.3	17.9	14.4	19.5	31.5	31.5
ASTR	10.0	19.0	20.0	11.0	9.0	4.0	0.0
**Dominican Rep. 1991**							
Using	7.1	17.9	25.5	18.4	36.5	100.0	100.0
Not using	10.7	9.9	15.3	20.1	17.4	24.3	24.3
All	10.4	10.8	16.8	19.9	20.0	30.8	30.8
ASTR	10.0	25.0	35.0	29.0	14.0	5.0	5.0
**Dominican Rep. 1996**							
Using	15.0	16.7	15.0	24.9	48.7	66.7	66.7
Not using	16.7	14.2	18.5	14.1	24.9	22.5	22.5
All	16.6	14.6	18.0	15.7	26.7	29.0	29.0
ASTR	22.0	34.0	35.0	21.0	14.0	6.0	0.0
**Dominican Rep. 1999**							
Using	13.8	10.1	35.4	3.6	0.0	29.0	29.0
Not using	18.1	20.3	22.8	26.8	21.6	73.6	73.6
All	17.6	19.0	25.3	24.6	20.4	53.0	53.0
ASTR	21.0	36.0	44.0	31.0	13.0	9.0	1.0
**Dominican Rep. 2002**							
Using	20.1	23.3	14.9	24.7	26.5	49.2	49.2
Not using	13.6	14.7	14.5	18.3	16.0	43.4	43.4
All	14.3	16.0	14.6	19.1	17.5	44.0	44.0
ASTR	19.0	36.0	25.0	23.0	9.0	6.0	1.0
**Guatemala 1995**							
Using	0.0	19.4	2.8	13.7	5.4	16.5	16.5
Not using	4.5	4.9	5.2	5.9	7.6	15.4	15.4
All	4.4	5.6	5.1	6.2	7.5	15.5	15.5
ASTR	6.0	15.0	13.0	13.0	11.0	10.0	2.0
**Guatemala 1998**							
Using	11.7	5.9	14.1	3.5	0.4	73.5	73.5
Not using	5.7	3.7	6.1	6.4	4.5	13.3	13.3
All	6.0	3.8	6.9	6.2	4.2	17.2	17.2
ASTR	7.0	11.0	18.0	12.0	6.0	12.0	1.0
**Guatemala 2014**							
Using	8.9	9.9	9.0	9.9	16.3	22.2	22.2
Not using	7.4	5.0	7.5	8.6	9.7	21.3	21.3
All	7.4	5.5	7.7	8.8	10.8	21.5	21.5
ASTR	7.0	10.0	12.0	11.0	8.0	7.0	1.0
**Guyana 2009**							
Using	17.6	24.9	48.0	41.4	30.3	20.6	20.6
Not using	12.5	16.5	20.6	21.5	30.9	52.4	52.4
All	12.7	17.7	25.2	24.9	30.9	51.1	51.1

	15-19	20-24	25-29	30-34	35-39	40-44	45-49
ASTR	15.0	35.0	39.0	34.0	25.0	14.0	4.0
**Honduras 2005**							
Using	6.8	11.8	16.3	8.3	20.0	16.9	16.9
Not using	6.8	6.6	6.9	9.3	14.0	26.9	26.9
All	6.8	7.4	8.5	9.1	15.1	25.2	25.2
ASTR	7.0	13.0	14.0	12.0	13.0	11.0	2.0
**Honduras 2011**							
Using	13.4	11.6	12.7	13.3	18.5	45.7	45.7
Not using	8.0	7.3	8.7	10.6	14.0	27.3	27.3
All	8.5	7.7	9.1	10.9	14.6	29.8	29.8
ASTR	9.0	13.0	14.0	12.0	11.0	8.0	1.0
**Nicaragua 1998**							
Using	12.9	15.3	13.4	5.2	7.2	17.0	17.0
Not using	4.7	8.3	8.3	7.2	11.7	9.1	9.1
All	5.4	9.0	8.9	7.1	11.4	9.4	9.4
ASTR	7.0	19.0	16.0	9.0	10.0	3.0	1.0
**Peru 1991**							
Using	8.4	7.4	12.7	13.8	21.8	26.2	26.2
Not using	5.7	6.9	8.8	11.8	13.6	14.3	14.3
All	6.3	7.0	10.0	12.5	16.3	18.6	18.6
ASTR	4.0	13.0	20.0	21.0	19.0	10.0	3.0
**Peru 1996**							
Using	14.1	8.6	11.9	15.2	11.9	18.4	18.4
Not using	6.4	7.4	7.9	11.3	12.9	18.9	18.9
All	8.1	7.7	9.1	12.6	12.6	18.7	18.7
ASTR	7.0	15.0	16.0	20.0	14.0	10.0	2.0
**Peru 2000**							
Using	13.6	7.3	15.1	14.3	19.3	21.8	21.8
Not using	8.8	6.6	7.9	11.4	12.4	15.0	15.0
All	9.6	6.7	9.7	12.2	14.5	17.1	17.1
ASTR	7.0	10.0	14.0	16.0	13.0	7.0	1.0
**Peru 2004**							
Using	16.9	14.5	13.2	11.2	16.7	24.7	24.7
Not using	8.8	7.2	10.1	9.2	12.0	26.3	26.3
All	10.6	9.2	11.0	9.8	13.4	25.8	25.8
ASTR	7.0	13.0	15.0	12.0	11.0	9.0	2.0
**Peru 2007**							
Using	17.7	14.3	18.5	18.1	18.9	23.2	23.2
Not using	11.5	10.0	10.4	11.4	15.5	31.3	31.3
All	13.3	11.3	12.9	13.4	16.7	28.8	28.8
ASTR	10.0	16.0	18.0	15.0	13.0	11.0	2.0
**Peru 2009**							
Using	18.7	18.7	15.2	18.2	20.6	14.2	14.2
Not using	7.7	11.3	10.5	12.0	16.9	29.1	29.1
All	11.4	13.8	11.9	13.9	17.9	24.0	24.0
ASTR	9.0	19.0	17.0	18.0	15.0	9.0	1.0
**Peru 2010**							

	15-19	20-24	25-29	30-34	35-39	40-44	45-49
Using	16.3	16.9	22.9	22.4	27.2	31.5	31.5
Not using	10.2	11.2	11.5	13.6	17.6	28.1	28.1
All	12.2	13.0	14.7	16.4	20.7	29.1	29.1
ASTR	9.0	18.0	21.0	19.0	17.0	12.0	1.0
**Peru 2011**							
Using	15.0	17.9	20.2	20.2	23.2	36.0	36.0
Not using	12.3	11.1	10.2	13.1	13.9	29.4	29.4
All	13.2	13.3	13.2	15.2	17.0	31.7	31.7
ASTR	9.0	19.0	19.0	19.0	15.0	12.0	1.0
**Paraguay 1990**							
Using	14.5	21.3	17.9	18.8	20.9	33.7	33.7
Not using	7.8	6.0	10.0	9.2	13.9	15.8	15.8
All	8.4	8.3	11.3	10.8	15.1	18.2	18.2
ASTR	9.0	19.0	27.0	24.0	25.0	16.0	3.0
**South and Southeast Asia**							
**India 2005**							
Using	13.4	20.5	28.2	43.6	37.0	48.4	48.4
Not using	12.2	10.0	11.2	15.0	17.3	9.9	9.9
All	12.2	10.4	12.0	17.0	19.3	13.6	13.6
ASTR	13.0	24.0	19.0	13.0	6.0	1.0	0.0
**Indonesia 2012**							
Using	29.0	5.6	3.3	12.4	19.9	17.5	17.5
Not using	9.1	7.9	9.0	11.7	15.5	21.2	21.2
All	9.4	7.8	8.7	11.7	16.1	20.7	20.7
ASTR	5.0	12.0	14.0	14.0	12.0	5.0	1.0
**Cambodia 2010**							
Using	26.9	31.7	37.9	60.2	57.9	74.6	74.6
Not using	16.7	14.2	16.7	21.6	32.5	44.2	44.2
All	16.9	14.5	18.3	25.2	35.2	47.9	47.9
ASTR	9.0	29.0	37.0	41.0	39.0	26.0	4.0
**Cambodia 2014**							
Using	74.1	35.7	44.8	52.6	75.1	83.1	83.1
Not using	15.9	16.1	19.7	20.9	31.4	55.0	55.0
All	17.2	17.1	22.6	25.4	39.3	59.4	59.4
ASTR	12.0	34.0	44.0	35.0	33.0	25.0	6.0
**Nepal 2011**							
Using	42.2	18.5	48.5	63.5	35.6	11.1	11.1
Not using	11.4	10.2	16.5	16.8	22.9	23.1	23.1
All	11.7	10.4	18.4	20.9	24.7	21.3	21.3
ASTR	11.0	22.0	28.0	19.0	12.0	4.0	1.0
**Nepal 2016**							
Using	30.8	24.2	46.0	60.9	56.8	50.0	50.0
Not using	13.3	15.3	19.0	28.5	41.7	48.0	48.0
All	13.8	15.6	20.5	31.3	42.9	48.5	48.5
ASTR	14.0	32.0	32.0	27.0	14.0	6.0	2.0
**Philippines 1993**							
Using	13.9	7.8	12.7	10.3	12.4	27.1	27.1

	15-19	20-24	25-29	30-34	35-39	40-44	45-49
Not using	10.3	7.2	8.1	8.0	13.5	21.9	21.9
All	10.5	7.3	8.7	8.3	13.3	22.7	22.7
ASTR	6.0	15.0	21.0	16.0	18.0	15.0	2.0
**Philippines 1998**							
Using	11.9	9.0	5.8	15.3	13.5	32.4	32.4
Not using	13.0	7.6	8.2	10.1	15.7	25.1	25.1
All	12.9	7.8	7.7	11.3	15.2	26.5	26.5
ASTR	7.0	15.0	18.0	20.0	20.0	14.0	3.0
**Philippines 2003**							
Using	16.3	6.5	8.0	14.9	11.1	17.6	17.6
Not using	9.0	8.6	8.2	9.9	13.4	26.6	26.6
All	9.5	8.4	8.1	10.8	13.1	25.0	25.0
ASTR	6.0	16.0	17.0	17.0	14.0	14.0	2.0
**Timor Leste 2009**							
Using	0.0	0.0	0.0	14.4	13.7		
Not using	4.7	2.3	2.6	2.4	2.7	5.3	5.3
All	4.7	2.3	2.6	2.5	2.8	5.3	5.3
ASTR	3.0	5.0	7.0	7.0	6.0	5.0	3.0
**Timor Leste 2016**							
Using			0.0	0.0	0.0	0.0	0.0
Not using	5.4	3.5	2.9	2.6	4.8	3.6	3.6
All	5.4	3.5	2.8	2.6	4.7	3.6	3.6
ASTR	2.0	7.0	7.0	5.0	6.0	2.0	1.0

Note: Cells left in blank correspond to categories with less than 10 unweighted pregnancies.
