# Supplementary material for: Reported patterns of pregnancy termination from Demographic and Health Surveys
Source: PLoS One. 2019 Aug 19;14(8):e0221178. doi: 10.1371/journal.pone.0221178 (PMC6699730; doi:10.1371/journal.pone.0221178)
Supplement: S5 Table — (PDF) [file pone.0221178.s007.pdf]

	In-union						Not-in-union	
	15-19	20-24	25-29	30-34	35-39	40-49	15-24	25-49
**Africa**								
**Angola 2015**								
Using	*12.4*	59.1	*8.7*	*10.8*	*11.8*	*22.3*	0	*21.3*
Not using	6.3	5.5	5.1	5.5	7.6	15.6	7.3	7.8
All	6.3	6	5.1	5.5	7.6	15.6	7.2	7.8
**Burkina Faso 2010**								
Using	*12.4*	17.2	6.2	18.5	*11.8*	*22.3*	*14.6*	*21.3*
Not using	7	4.1	3.6	4.7	4.7	9	8.5	18.5
All	7	4.2	3.6	4.8	4.8	9	8.7	18.5
**Benin 2011**								
Using	*12.4*	*9.9*	0	*10.8*	*11.8*	*22.3*	*14.6*	*21.3*
Not using	3.8	3.5	2.8	3.8	5.1	3.5	4.9	10.2
All	3.8	3.6	2.8	3.8	5.1	3.6	4.9	10.3
**Burundi 2010**								
Using	*12.4*	13.5	0	2.8	0	42.2	*14.6*	*21.3*
Not using	11.4	5.9	4.4	7	8.9	18.2	8.7	1.8
All	11.4	6	4.3	6.9	8.7	19	8.8	1.8
**Burundi 2016**								
Using	*12.4*	5.1	0	6.4	13	*22.3*	*14.6*	*21.3*
Not using	10.3	7.7	6.6	6.7	10.9	16.9	5.7	4.9
All	10.3	7.7	6.5	6.7	10.9	17	5.7	4.9
**Ethiopia 2005**								
Using	0	27.4	14.5	17.5	21.9	*22.3*	*14.6*	*21.3*
Not using	3	4.2	3.3	4.2	5	9.1	10.8	0
All	3	4.5	3.4	4.2	5.3	9.2	10.9	1.1
**Ethiopia 2011**								
Using	1.6	6.1	6.2	9.3	3.8	*22.3*	13.8	*21.3*
Not using	6.1	6.6	3.3	5.5	8.9	15.9	8.4	3.3
All	6	6.5	3.5	5.7	8.6	16	8.7	3.8
**Ethiopia 2016**								
Using	*12.4*	0	19.1	12.5	4.1	*22.3*	*14.6*	*21.3*
Not using	4.5	3.5	4.4	5.2	6.7	14.8	9.9	12.3
All	4.5	3.5	4.6	5.3	6.7	14.8	10	12.5
**Ghana 2008**								
Using	*13.9*	10	17.7	4.6	19	*30*	39.2	*30.1*
Not using	9.4	10.7	8.5	14.5	13.2	20.7	26.8	34
All	9.7	10.6	9.3	13.9	13.8	21.2	28.1	33.4
**Ghana 2014**								
Using	*13.9*	25.3	23.4	15.8	22.8	*30*	52.5	80.4
Not using	11.4	13.7	11.7	14.8	17.3	22.6	32.4	39.6
All	11.5	14.1	12.1	14.9	17.5	22.8	33.7	42.7
**Kenya 1998**								
Using	5.4	1.3	5.3	3	12.5	0	5	24.2
Not using	4.5	5.1	5.4	5.4	5.5	9.6	6.6	8.3
All	4.5	4.8	5.4	5.1	5.9	8.6	6.4	9.7

	In-union						Not-in-union	
	15-19	20-24	25-29	30-34	35-39	40-49	15-24	25-49
**Kenya 2003**								
Using	17.9	6.9	1.3	5.3	11.1	*22.3*	7	7.4
Not using	7.6	5.4	3.9	5.3	8.2	8.2	4.3	6.1
All	8.1	5.5	3.7	5.3	8.6	9.2	4.5	6.2
**Kenya 2008**								
Using	6	8	5.5	4.1	4.3	15.7	0	5.1
Not using	3.3	4.4	6.5	4.4	12.5	19	3.1	9
All	3.4	4.8	6.3	4.4	11.4	18.6	2.9	8.3
**Comoros 2012**								
Using	*12.4*	*9.9*	*8.7*	*10.8*	*11.8*	*22.3*	*14.6*	*21.3*
Not using	5.7	4.6	8.1	5.8	12	18.1	6.6	17.9
All	5.7	4.7	8.1	5.9	12	18.2	6.6	17.9
**Liberia 2013**								
Using	*13.9*	29.3	39.4	*23.2*	36.4	*30*	38.7	*30.1*
Not using	7.7	8.3	12.4	11.9	11.7	27	11.7	10.9
All	7.8	8.6	13.1	12	12.5	27	12.4	10.9
**Lesotho 2009**								
Using	7.7	2.7	2.9	6.6	11.1	*22.3*	15.3	*21.3*
Not using	4.3	6	4.6	5.1	9.3	4.1	3.7	7.7
All	4.5	5.7	4.4	5.3	9.6	5.3	4.5	8.6
**Lesotho 2014**								
Using	22.4	1.2	1.9	11.7	6.8	*22.3*	5	6.9
Not using	9.8	4.8	10.1	10.1	12.4	13.1	7.1	10.5
All	10.7	4.4	9.2	10.3	11.4	14.3	6.9	10
**Morocco 1992**								
Using	*12.4*	4.6	9.2	9.4	13.6	25.5	*14.6*	*21.3*
Not using	9.1	7.1	7.7	8.6	8.9	8.5	*7.5*	*9.2*
All	9.2	6.9	7.8	8.8	9.7	11.8	7.5	14.4
**Morocco 2003**								
Using	18.1	11.1	10.2	12.3	15.8	21.8	*14.6*	*21.3*
Not using	10	8.5	8.2	11.7	16.9	29.8	8.6	*9.2*
All	10.7	8.9	8.6	11.9	16.6	27.2	8.9	9.2
**Madagascar 2008**								
Using	11	8.3	14.3	12	11	21.1	31.8	*21.3*
Not using	5.2	5.7	6.4	6.8	7.6	11.4	11.6	9.2
All	5.4	5.9	7	7.2	7.8	11.8	12.5	10.5
**Mali 2012**								
Using	*12.4*	*9.9*	*8.7*	*10.8*	*11.8*	*22.3*	*14.6*	*21.3*
Not using	4.5	3.5	3.8	3.4	4.6	7.2	5.7	2.4
All	4.5	3.5	3.8	3.4	4.6	7.2	5.7	2.4
**Malawi 2004**								
Using	12.5	0.6	0	7.5	6	5.1	*14.6*	*21.3*
Not using	5.8	4.4	4.3	5	7	6.4	5	5.5
All	5.9	4.2	4.1	5.1	7	6.3	5	5.6
**Malawi 2010**								

	In-union						Not-in-union	
	15-19	20-24	25-29	30-34	35-39	40-49	15-24	25-49
Using	6.7	2.4	2.5	5.9	5.5	4.5	1.3	*21.3*
Not using	6.8	3.7	5.1	5.4	5.3	13.2	7.9	16.8
All	6.8	3.6	4.9	5.5	5.3	12.7	7.7	17.3
**Malawi 2015**								
Using	9.4	2.6	8.2	10	3.3	*22.3*	*14.6*	*21.3*
Not using	6.2	4.9	5.3	4.3	5.8	10.2	7.6	6.3
All	6.3	4.8	5.4	4.5	5.7	10.3	7.6	6.5
**Mozambique 2011**								
Using	*12.4*	34.4	3.8	*10.8*	*11.8*	*22.3*	33.2	*21.3*
Not using	7.7	4.2	5.5	4.4	5.8	8.7	10.7	10.8
All	7.7	4.4	5.5	4.4	5.8	8.8	11.1	10.8
**Nigeria 2008**								
Using	15.7	6.1	3.9	8	15.2	10	48.5	31.4
Not using	5.2	5.2	4.7	6.2	10	11.3	16.2	13.4
All	5.4	5.3	4.7	6.4	10.3	11.2	19	15.4
**Nigeria 2013**								
Using	18.6	16.8	7.5	7.4	26.7	20.8	43.7	66.8
Not using	6.3	5.4	6.1	7.2	9.3	13.1	13.7	17
All	6.3	5.6	6.1	7.2	9.9	13.3	15.2	19.3
**Niger 2012**								
Using	*12.4*	*9.9*	8.5	*10.8*	*11.8*	*22.3*	*14.6*	*21.3*
Not using	9.1	3.8	5.8	7.9	9.5	17.3	6.8	0
All	9.1	3.9	5.9	7.9	9.5	17.3	6.8	0
**Namibia 2006**								
Using	*12.4*	3.7	6	6.6	3	*22.3*	1.6	4.7
Not using	1.5	6	4.9	7.6	9	16.4	3.3	4
All	1.6	5.8	5	7.5	8.5	16.6	3.1	4.1
**Namibia 2013**								
Using	*12.4*	4.8	10.1	3.9	10.6	*22.3*	2.4	5
Not using	2.5	5.5	11.5	6	7.9	14.4	4.8	8.5
All	2.8	5.5	11.4	5.8	8.1	15	4.4	8.2
**Rwanda 2010**								
Using	*12.4*	4	3.2	11.8	12.7	17.7	*14.6*	*21.3*
Not using	5.1	7.6	5.1	5.3	8.3	18.1	5.6	7.1
All	5.1	7.5	5	5.7	8.6	18	5.6	7.2
**Rwanda 2014**								
Using	*12.4*	4	8.8	10.5	17.5	17.6	*14.6*	*21.3*
Not using	10.5	7.2	5.9	7.7	10	16.2	5.6	6.8
All	10.6	7.1	6.1	8	11	16.4	5.7	7.2
**Sierra Leone 2008**								
Using	*12.4*	7.7	9.1	0	0	*22.3*	18.9	*21.3*
Not using	5.4	5.1	6.2	6	6.4	10.1	8.5	6.2
All	5.4	5.2	6.3	5.8	6.3	10.3	8.8	7.1
**Sierra Leone 2013**								
Using	*12.4*	3.5	16	57.7	11.2	*22.3*	12.5	*21.3*

	In-union						Not-in-union	
	15-19	20-24	25-29	30-34	35-39	40-49	15-24	25-49
Not using	6.2	5.4	5.9	5.7	7.9	10.6	7.9	11.5
All	6.2	5.4	6	6.1	8	10.8	8	11.8
**Senegal 2012**								
Using	*12.4*	*9.9*	*8.7*	11.4	*11.8*	*22.3*	*14.6*	*21.3*
Not using	11.2	8.7	8.4	7.4	10.4	17.2	8.3	1.1
All	11.2	8.7	8.4	7.5	10.4	17.3	8.5	1.1
**Senegal 2014**								
Using	*12.4*	*9.9*	19.2	*10.8*	*11.8*	*22.3*	*14.6*	*21.3*
Not using	6.5	7.7	6.7	11.1	10.5	10.1	7.6	4
All	6.5	7.8	6.8	11.1	10.5	10.2	7.6	4
**Senegal 2015**								
Using	*12.4*	*9.9*	*8.7*	0	*11.8*	*22.3*	*14.6*	*21.3*
Not using	9.2	6.5	6	9.4	15.6	20.8	5	8.2
All	9.2	6.5	6.1	9.2	15.5	20.8	5	8.2
**Senegal 2016**								
Using	*12.4*	*9.9*	0	16.6	6.6	*22.3*	*14.6*	*21.3*
Not using	5.4	8.1	5.7	10.7	14.1	17.5	11	18.7
All	5.4	8.1	5.6	10.9	13.7	17.6	11	18.7
**Senegal 2017**								
Using	*12.4*	*9.9*	*8.7*	4.9	10.7	*22.3*	*14.6*	*21.3*
Not using	10.4	8.4	8	9.5	15.7	21.1	6.1	14
All	10.4	8.5	8	9.5	15.6	21.1	6.1	14.2
**Tanzania 2004**								
Using	*12.4*	8.7	18.7	10.9	2.5	*22.3*	14.5	*21.3*
Not using	8.8	5.7	6.1	8.5	11.2	22.3	10.1	16.2
All	8.8	5.9	6.9	8.6	10.8	22.3	10.2	16.3
**Tanzania 2010**								
Using	*12.4*	2.5	3.3	6	7.7	4.4	3.9	*21.3*
Not using	8.5	7.2	7.1	7.3	13	17.2	7.3	0.8
All	8.5	7	6.9	7.2	12.7	16	7.2	2.2
**Tanzania 2015**								
Using	5.5	6.3	15.1	11.5	9.6	15.5	19.5	*21.3*
Not using	9	7.7	8.6	8.7	12.3	20.1	9.3	14.4
All	8.9	7.6	9	8.9	12.1	19.7	9.8	15
**Uganda 2006**								
Using	25.1	9.7	8.7	7.9	25.1	*22.3*	1.6	*21.3*
Not using	9.4	7.3	6.4	9.2	13.9	23.5	13	13.7
All	10	7.4	6.6	9.1	14.6	23.4	12.2	14
**Uganda 2011**								
Using	0	12.6	4.1	4.4	18.2	*22.3*	8	*21.3*
Not using	12.9	6.6	8	8.6	12.5	25.5	13.3	17.3
All	12.5	6.9	7.8	8.3	12.7	25.3	13.1	17.5
**Uganda 2016**								
Using	7	15.9	14.1	17.9	27.4	17.7	35.2	*30.1*
Not using	10.3	9.1	7.9	10.5	15	25.5	10.5	18.3

	In-union						Not-in-union	
	15-19	20-24	25-29	30-34	35-39	40-49	15-24	25-49
All	10.3	9.4	8.2	10.8	15.7	25	10.9	18.9
**Zambia 2007**								
Using	6.1	5.2	5	11	2.3	0	15.6	*21.3*
Not using	7.7	5.2	6.2	5.4	6	10.8	5.7	12.4
All	7.6	5.2	6.1	6.1	5.5	9.7	6.4	13.2
**Zambia 2013**								
Using	4	2.4	3.5	2.6	14.6	3	2.8	*21.3*
Not using	5.6	5.5	4.2	5.3	6.4	13.9	5.2	11.4
All	5.6	5.3	4.1	5.1	7.2	12.7	5.2	11.7
**Zimbabwe 1994**								
Using	6	5.5	7.2	9.8	14.2	27.3	5.3	24.9
Not using	10.4	8.4	4.9	6	7	24.5	6.6	12.5
All	10	8.1	5.2	6.4	8.3	24.9	6.5	13.6
**Zimbabwe 1999**								
Using	13.2	16.5	5.5	0	4.2	*22.3*	10	*21.3*
Not using	8.5	7	6.5	7.1	11.6	18.9	8.2	8.2
All	8.8	7.9	6.4	6.2	10.7	19.1	8.3	9.4
**Zimbabwe 2005**								
Using	30.4	2.3	5.2	2.7	6.9	23.9	5.5	0
Not using	9.4	5.9	7.1	4.6	12.1	18.2	5.8	10.9
All	10.6	5.5	6.8	4.4	11.6	18.8	5.8	10
**Zimbabwe 2010**								
Using	*12.4*	5	3.3	11.3	5.9	0	21.8	*21.3*
Not using	9.7	5	7.3	8	8.1	7.7	5.4	12.4
All	9.8	5	7	8.4	7.8	6.8	5.7	12.7
**Zimbabwe 2015**								
Using	0	4	8.5	9.7	11.8	10	*14.6*	*21.3*
Not using	9.4	9.2	5	7.1	11.1	26.6	9.7	6
All	9	8.7	5.4	7.4	11.1	24.7	9.8	6.3
**Central and West Asia & Europe**								
**Albania 2008**								
Using	*13.9*	24.1	14.9	20.2	23.9	*30*	*26.5*	*30.1*
Not using	12	6.9	11	22.4	38.8	71.3	9.1	0
All	12.1	10.2	12	21.8	36.1	71.3	11.8	4.2
**Albania 2017**								
Using	*12.4*	20	16.3	24.9	*11.8*	*22.3*	*14.6*	*21.3*
Not using	6.7	6.9	7.6	7.6	19.6	38.9	5.5	0
All	6.8	7.6	8	8.5	19.3	37.7	5.6	0
**Armenia 2000**								
Using	53.4	75.7	86	88.6	93.7	97.1	*49.4*	*66.1*
Not using	18.5	30.6	50.1	73.1	74.6	86.7	*36.7*	*46.5*
All	22.6	45.3	68.7	81.4	85.3	91.9	36.7	46.5
**Armenia 2005**								
Using	*61.9*	64.3	86	96.3	89.3	74.3	*49.4*	*66.1*
Not using	19	24.3	45.3	59.5	72.6	91.5	*36.7*	25.5

	In-union						Not-in-union	
	15-19	20-24	25-29	30-34	35-39	40-49	15-24	25-49
All	20.7	31.9	58.8	74	80.2	84.5	46.5	28.2
**Armenia 2010**								
Using	*33.7*	49.1	69.2	72.7	75.5	89.1	*23.6*	*26.1*
Not using	20.2	20.6	32.4	50.2	47.5	54.6	20.8	*22.5*
All	20.4	23.5	41.1	57.2	54.8	68.8	20.8	22.5
**Armenia 2015**								
Using	*33.7*	56	76.7	78.3	95.5	91.4	*23.6*	*26.1*
Not using	13.7	18.7	27.8	32	29.8	53.5	32.3	*22.5*
All	14	22.5	34.9	41	43.8	67.4	32.3	22.9
**Azerbaijan 2006**								
Using	94.7	64.9	78.8	88.6	86.7	100	*49.4*	*66.1*
Not using	17.9	29.5	49.3	57	72.6	84.1	31	35.3
All	20.6	33.4	56.2	67.5	77.4	89.2	31	35.3
**Kazakhstan 1999**								
Using	46.7	59.1	80.5	86.3	92.7	84	79.1	88.9
Not using	22.4	28.8	39.7	37.8	50.9	55.3	49.8	65.2
All	25.2	33.8	50.6	51.6	63.2	67	54	72.5
**Kyrgyz Rep. 2012**								
Using	*33.7*	49.9	50.5	52.6	48	*73.7*	*23.6*	*26.1*
Not using	9.3	17.5	22.5	21.8	29.8	27.9	19.9	27.7
All	10	19	24.4	24.4	31.4	29.6	20.1	27.6
**Moldova 2005**								
Using	60.1	59.1	71.9	71.9	70.2	90	53.1	88.3
Not using	25	26.9	31.5	34.3	66.8	74.7	43.8	65.3
All	32.4	34.8	44.1	48.2	68.3	80.5	45.1	70.3
**Tajikistan 2012**								
Using	*33.7*	26	52	67.3	65.6	*73.7*	*23.6*	*26.1*
Not using	12.1	10.7	15.1	19.2	27.9	41.1	14.7	25
All	12.1	10.8	16	20.4	29.4	42.9	14.7	25
**Tajikistan 2017**								
Using	*13.9*	21.7	41	*23.2*	*25.8*	*30*	*26.5*	*30.1*
Not using	9.4	10.7	16	22.8	32.5	51.7	11.5	25.1
All	9.4	10.7	16.3	22.8	32.3	51.6	11.5	25.1
**Turkey 1998**								
Using	48	33.9	32.7	50	71.3	77.5	*23.6*	*26.1*
Not using	13.7	14.4	17.7	26.4	29.1	48.3	*18.3*	*22.5*
All	17.6	17.7	21	32.9	43.9	61.4	18.3	22.5
**Turkey 2003**								
Using	18.6	21.8	29.3	50.6	57.5	52	23.6	*26.1*
Not using	17.8	13.3	16.9	18	36.7	30.3	*18.3*	*22.5*
All	17.9	15	20.3	29.9	46	41		22.5
**Ukraine 2007**								
Using	*33.7*	45.3	75.9	71.5	76.8	85.6	40.3	*26.1*
Not using	13	15.9	27.1	28.9	42.3	53.8	12.5	41.9
All	15.4	21.2	39.6	41.2	57.4	68.2	20.6	39.1

	In-union						Not-in-union	
	15-19	20-24	25-29	30-34	35-39	40-49	15-24	25-49
**Latin America**								
**Bolivia 1994**								
Using	12.6	7.6	14.6	17.2	13.7	20	19.9	0
Not using	7.2	6.2	7.9	10	10.6	6.4	5.5	11.1
All	8.1	6.5	9.2	11.8	11.2	9.4	6.9	9.9
**Bolivia 2008**								
Using	8.7	13.9	15	16.6	18.8	24.3	13.3	18.6
Not using	9.6	10	10.8	14.4	16.7	17.5	10.2	16.8
All	9.4	11	11.8	15	17.3	19.1	10.8	17.3
**Brazil 1996**								
Using	20.4	20	10.5	11	24.8	33.5	20.6	13.8
Not using	9.7	9.1	11.9	11.1	20.7	30.3	14.3	17
All	10.9	11.1	11.6	11	21.6	31	15.6	16.2
**Colombia 1990**								
Using	7	16.8	16	25.1	10.8	26.2	21.5	6.7
Not using	11.6	10.3	10.2	13.6	19.2	35.1	5.5	14.5
All	11.1	11.4	11.6	16.3	16.7	32.4	7.5	13
**Colombia 1995**								
Using	18.6	14.9	15.2	15.6	9.6	26.1	7.7	15.5
Not using	8.6	9.9	9.5	11.1	9.3	23.9	7.1	14.9
All	10.2	11.2	11.1	12.6	9.4	24.7	7.2	15.1
**Colombia 2000**								
Using	8	14.8	12.9	15.2	25.8	29	19.2	24.5
Not using	8.6	14.2	16.4	16.5	21.1	25.6	13.4	18.4
All	8.5	14.4	15.2	16.1	23	26.9	15.4	20.5
**Colombia 2005**								
Using	18.3	21	16.6	21.6	33.1	19.3	20.4	27.5
Not using	10.4	13.8	14.3	17	20.6	29	18.8	22.3
All	12.1	15.6	15	18.4	24.8	25.5	19.3	23.8
**Colombia 2010**								
Using	13	22.1	17.3	27.4	22.3	32.9	20	21.4
Not using	11.7	16.1	15.1	19	25.2	35.1	15.3	16.8
All	12	17.3	15.6	20.8	24.5	34.5	16.4	17.8
**Colombia 2015**								
Using	11.5	16.6	25.1	21.2	19.5	16.6	19.6	17.1
Not using	7.8	12.3	15.7	13.7	21.6	37.1	14.7	14.7
All	8.5	13.2	17.7	14.8	21.1	32	15.9	15.2
**Dominican Rep. 1991**								
Using	5.7	16.2	26.3	18.4	36.5	*30*	27	*30.1*
Not using	11	9.8	14.8	20.1	18	25.1	9.5	23
All	10.7	10.5	16.6	19.9	20.5	25.5	12.2	23.8
**Dominican Rep. 1996**								
Using	14.9	15.8	16.7	20.5	*25.8*	*30*	22.8	41.7
Not using	16.2	12.6	18.2	14.2	24.1	19.4	24.3	22.8
All	16.1	13	18	15	24.2	20.2	24.1	27.3

	In-union						Not-in-union	
	15-19	20-24	25-29	30-34	35-39	40-49	15-24	25-49
**Dominican Rep. 1999**								
Using	*13.9*	10.1	28.8	*23.2*	*25.8*	*30*	*26.5*	*30.1*
Not using	13.7	18.2	20.3	22.9	20.5	*31.9*	45.4	56.5
All	13.7	17.1	22	22.9	20.8	30.5	45	53.9
**Dominican Rep. 2002**								
Using	18.1	21.1	14	25.5	26.9	50.9	35	21.5
Not using	11.7	12.3	13.8	16.8	14.3	40.5	26.5	29.6
All	12.4	13.7	13.8	17.9	15.9	41.7	27.5	28.6
**Guatemala 1995**								
Using	0	21.3	2.8	13.7	3.2	16.5	*14.6*	*21.3*
Not using	4.8	4.9	5.3	6	7	15.3	4	8.3
All	4.7	5.6	5.2	6.3	6.8	15.3	4.5	8.5
**Guatemala 1998**								
Using	17.2	7	14.1	3.6	0.6	*22.3*	*14.6*	*21.3*
Not using	6.3	3.2	6	6.5	4.5	13.3	5.6	6.5
All	6.7	3.4	6.9	6.3	4.3	13.6	6.3	9.4
**Guatemala 2014**								
Using	10.3	10.3	9.4	9.9	16.3	22.2	4.3	0
Not using	7.4	5.2	7.3	8.6	9.6	19.9	5.7	13.3
All	7.6	5.8	7.5	8.8	10.8	20.2	5.6	12.5
**Guyana 2009**								
Using	*13.9*	21	49	41	30.3	*30*	23.7	43.6
Not using	9.8	16	19.3	21.8	32.8	53.3	17.3	22.5
All	10	16.8	24.1	24.8	32.5	52.4	17.9	26.9
**Honduras 2005**								
Using	3.9	11.5	15.1	8.4	20	16.9	16.1	*21.3*
Not using	6.5	6.6	6.7	9.2	14.1	26.1	7.3	14
All	6.3	7.4	8.1	9.1	15.2	24.5	8.6	14.9
**Honduras 2011**								
Using	13.9	12.4	13.1	14.1	17	47.3	10.6	11
Not using	8.6	7.6	9	10.4	14	26.7	5.6	8.5
All	9	8.1	9.5	10.9	14.4	29.5	6.3	8.8
**Nicaragua 1998**								
Using	13.3	15.9	13.5	5.4	7.2	*22.3*	*14.6*	*21.3*
Not using	4.6	7.9	8.2	6.9	11.7	9.3	10.2	13.3
All	5.3	8.6	8.8	6.7	11.3	9.8	10.4	14.2
**Peru 1991**								
Using	6	5.5	13.1	13.1	21.6	26.7	15.3	16.2
Not using	4.9	6.7	8.9	11.4	13.5	13.9	7.6	12.4
All	5.1	6.4	10.2	12	16.2	18.6	9.3	13.2
**Peru 1996**								
Using	13.6	8.9	11.1	14.4	12.1	18.1	10.9	21.7
Not using	5.6	7.3	7.8	11.4	12.8	19.3	8	10.3
All	7.4	7.7	8.8	12.4	12.5	18.8	8.7	13.6
**Peru 2000**								

	In-union						Not-in-union	
	15-19	20-24	25-29	30-34	35-39	40-49	15-24	25-49
Using	8.9	6.4	15.1	12.8	17.8	21.6	13.4	24.7
Not using	7.3	6.9	7.8	10.9	12.7	12.1	8.4	13
All	7.5	6.8	9.6	11.5	14.3	15.3	9.6	15.8
**Peru 2004**								
Using	19.1	14.1	11.9	11.5	16.7	22.2	15.2	20.7
Not using	7	5.9	10	9.1	12	27.3	11.3	10.8
All	9.2	8	10.6	9.8	13.4	25.7	12.4	13.1
**Peru 2007**								
Using	8.8	11.7	17.8	16.2	17.9	23.8	22	28.8
Not using	11.4	11.1	11	11.3	16.5	31.8	9.3	9.6
All	10.8	11.3	13.2	12.7	17	29.2	13.7	14.9
**Peru 2009**								
Using	12.5	12.5	13.9	18.5	19.8	14.6	27	21.2
Not using	6.7	9.3	10.5	11.2	16	29.3	13	16.7
All	8.6	10.2	11.4	13.5	17.1	24.4	18.8	17.9
**Peru 2010**								
Using	12.2	14.4	20.2	19.1	25.4	29	21.3	47.4
Not using	9.3	9.9	10.8	13.2	17	29.4	13.5	17.3
All	10.1	11.2	13.5	15.1	19.7	29.3	16.5	26.1
**Peru 2011**								
Using	14.1	17	17.7	18.7	20.5	35.9	17.8	35.6
Not using	10.9	8.1	9.6	12.1	13.8	28.7	17.2	17.9
All	11.9	10.9	12	14.1	16.1	31.3	17.4	24
**Paraguay 1990**								
Using	9.7	20.5	17.3	17.5	19.5	34.5	26.9	*21.3*
Not using	9.1	6.5	10.2	9	14.4	15.7	4.6	10
All	9.2	8.8	11.4	10.4	15.3	18.2	6.4	11.1
**South and Southeast Asia**								
**India 2005**								
Using	13.4	20.5	28.2	43.6	37	48.4	*26.5*	*30.1*
Not using	12.2	10	11.1	14.9	17.3	9.9	12.4	53.4
All	12.2	10.4	11.9	16.9	19.2	13.6	12.4	53.4
**Indonesia 2012**								
Using	29.8	5.6	3.3	12.4	19.9	17.5	*14.6*	*21.3*
Not using	10.2	8.1	9	11.7	15.6	21.1	5.2	9.7
All	10.5	7.9	8.7	11.7	16.2	20.6	5.3	9.8
**Cambodia 2010**								
Using	24	31.7	37.3	60.2	57.9	74.6	*23.6*	*26.1*
Not using	16	14.3	16.8	21.8	32.7	43.9	16.8	13.6
All	16.1	14.7	18.3	25.4	35.4	47.7	17	14.2
**Cambodia 2014**								
Using	74.1	35.7	44.9	52	75.1	83.1	*23.6*	*26.1*
Not using	15.4	15.8	19.7	21.1	31.5	55	24.9	17.7
All	16.8	16.9	22.5	25.4	39.3	59.4	24.9	18.4
**Nepal 2011**								

	In-union						Not-in-union	
	15-19	20-24	25-29	30-34	35-39	40-49	15-24	25-49
Using	32.1	17.7	48.5	63.5	35.6	*30*	*26.5*	*30.1*
Not using	11.5	10.1	16.5	16.9	22.9	23.1	13.2	*22.7*
All	11.7	10.3	18.4	21	24.7	24.1	13.7	22.7
**Nepal 2016**								
Using	31.3	24.2	46	60.9	56.8	50	*23.6*	*26.1*
Not using	13.5	15.3	19	28.6	41.7	48	6.5	*22.5*
All	14	15.7	20.5	31.4	42.9	48.5	6.7	22.5
**Philippines 1993**								
Using	13.9	7.9	12.8	10.3	12.4	27.1	*14.6*	*21.3*
Not using	9.3	7.5	8.3	8.1	13.4	22.1	8.8	3.8
All	9.5	7.5	8.9	8.4	13.3	22.9	8.8	4.1
**Philippines 1998**								
Using	6.6	7.6	5.9	15.1	13.5	32.4	30.9	*21.3*
Not using	14.2	7.6	8.6	10.3	16	24.9	7.7	2.7
All	13.5	7.6	7.9	11.4	15.4	26.3	9.6	3.5
**Philippines 2003**								
Using	19.7	6	8.1	14.9	11.3	17.6	8.4	*21.3*
Not using	9.6	9.2	8.5	9.8	13.6	26.7	5.8	5.5
All	10.4	8.8	8.4	10.7	13.2	25	5.9	6
**Timor Leste 2009**								
Using	*12.4*	*9.9*	*8.7*	14.4	*11.8*	*22.3*	*14.6*	*21.3*
Not using	4.5	2	2.6	2.4	2.8	5.3	8	0
All	4.6	2.1	2.7	2.5	2.8	5.3	8	0
**Timor Leste 2016**								
Using	*12.4*	*9.9*	*8.7*	*10.8*	*11.8*	*22.3*	*14.6*	*21.3*
Not using	5.2	3.8	2.9	2.7	4.7	3.6	2.7	2.9
All	5.2	3.8	2.9	2.7	4.8	3.7	2.7	2.9
**Cluster means**								
**Cluster 1**								
Using	12.4	9.9	8.7	10.8	11.8	22.3	14.6	21.3
Not using	7.3	6.2	6.5	7.4	10.1	16	7.5	9.2
**Cluster 2**								
Using	13.9	18.1	23	23.2	25.8	30	26.5	30.1
Not using	10.6	11.4	13.4	15.8	20.4	31.9	16.8	22.7
**Cluster 3**								
Using	33.7	37.4	51.4	61.6	68	73.7	23.6	26.1
Not using	14.4	15.6	21.5	26.8	34.9	45.6	18.3	22.5
**Cluster 4**								
Using	61.9	64.6	80.6	86.3	86.5	89.1	49.4	66.1
Not using	20.5	28	43.2	52.3	67.5	78.4	36.7	46.5

Note:
Values in italics correspond to imputed probabilities from the cluster means.
