# Supplementary material for: Reported patterns of pregnancy termination from Demographic and Health Surveys
Source: PLoS One. 2019 Aug 19;14(8):e0221178. doi: 10.1371/journal.pone.0221178 (PMC6699730; doi:10.1371/journal.pone.0221178)
Supplement: S6 Table — TFR: Total fertility rate. TTR: Total termination rate. TPR: Total pregnancy rate. GFR: General fertility rate. GTR: General termination rate. GPR: General pregnancy rate. (PDF) [file pone.0221178.s008.pdf]

			Probability PT (%)										
				Model estimates		Contraception (%)		Total rates			General rates		
Code	Survey	Cluster	T	IA	ST	Any	Modern	TFR	TTR	TPR	GFR	GTR	GPR
**Africa**													
AO	Angola 2015	1	6.7	1.6	5.1	13.3	12.5	6.2	0.5	6.7	216	15.4	231.4
BF	Burkina Faso 2010	1	5.0	1.1	3.9	15.3	14.3	6.0	0.3	6.3	206	10.8	216.8
BJ	Benin 2011	1	3.8	0.8	3.1	14.0	9.0	4.9	0.2	5.1	175	7.0	182.0
BU	Burundi 2010	1	7.3	1.8	5.5	13.4	11.0	6.4	0.6	7.0	203	16.0	219.0
BU	Burundi 2016	1	8.2	2.1	6.1	17.9	14.6	5.5	0.5	6.0	180	16.0	196.0
ET	Ethiopia 2005	1	4.3	0.9	3.4	10.3	9.7	5.4	0.3	5.7	179	8.1	187.1
ET	Ethiopia 2011	1	6.3	1.4	4.8	19.6	18.7	4.8	0.4	5.2	161	10.7	171.7
ET	Ethiopia 2016	1	5.3	1.2	4.1	25.3	24.9	4.6	0.3	4.9	156	8.7	164.7
GH	Ghana 2008	2	14.2	4.8	9.4	19.3	13.5	4.0	0.7	4.7	136	22.6	158.6
GH	Ghana 2014	2	18.2	7.2	10.9	22.8	18.2	4.2	0.9	5.1	143	31.7	174.7
KE	Kenya 1998	1	5.5	1.2	4.3	29.9	23.6	4.7	0.3	5.0	166	9.7	175.7
KE	Kenya 2003	1	5.6	1.2	4.3	28.4	22.7	4.9	0.3	5.2	171	10.1	181.1
KE	Kenya 2008	1	5.9	1.3	4.6	32.0	28.0	4.6	0.3	4.9	161	10.2	171.2
KM	Comoros 2012	1	7.6	1.9	5.7	13.7	9.9	4.3	0.4	4.7	142	11.7	153.7
LB	Liberia 2013	2	12.0	3.7	8.3	21.7	20.5	4.7	0.7	5.4	168	22.9	190.9
LS	Lesotho 2009	1	5.3	1.2	4.2	35.9	34.9	3.3	0.2	3.5	119	6.7	125.7
LS	Lesotho 2014	1	8.2	2.1	6.1	48.9	48.5	3.3	0.3	3.6	118	10.6	128.6
MA	Morocco 1992	1	8.5	2.2	6.3	22.9	19.7	4.0	0.4	4.4	127	11.8	138.8
MA	Morocco 2003	1	11.8	3.6	8.2	33.3	29.0	2.5	0.4	2.8	81	10.9	91.9
MD	Madagascar 2008	1	7.3	1.8	5.5	31.7	23.0	4.8	0.4	5.2	168	13.3	181.3
ML	Mali 2012	1	4.1	0.8	3.2	9.9	9.6	6.1	0.3	6.4	214	9.0	223.0
MW	Malawi 2004	1	4.9	1.1	3.9	25.7	22.4	6.0	0.3	6.3	215	11.2	226.2
MW	Malawi 2010	1	5.5	1.2	4.3	35.4	32.6	5.7	0.4	6.1	202	11.8	213.8
MW	Malawi 2015	1	5.7	1.3	4.4	46.0	45.2	4.4	0.3	4.7	158	9.5	167.5
MZ	Mozambique 2011	1	6.3	1.4	4.8	12.3	12.1	5.9	0.4	6.3	206	13.8	219.8
NG	Nigeria 2008	1	7.1	1.7	5.4	15.4	10.5	5.7	0.5	6.2	195	15.0	210.0
NG	Nigeria 2013	1	7.6	1.9	5.7	16.0	11.1	5.5	0.5	6.0	190	15.5	205.5

			Probability PT (%)										
				Model estimates		Contraception (%)		Total rates			General rates		
Code	Survey	Cluster	T	IA	ST	Any	Modern	TFR	TTR	TPR	GFR	GTR	GPR
NI	Niger 2012	1	7.0	1.7	5.4	12.5	11.0	7.6	0.7	8.3	269	20.4	289.4
NM	Namibia 2006	1	5.3	1.2	4.1	46.6	45.7	3.6	0.2	3.8	122	6.8	128.8
NM	Namibia 2013	1	6.9	1.6	5.3	50.2	49.7	3.6	0.3	3.9	125	9.3	134.3
RW	Rwanda 2010	1	7.1	1.7	5.4	28.6	25.2	4.6	0.4	5.0	151	11.6	162.6
RW	Rwanda 2014	1	7.9	2	5.9	30.9	27.8	4.2	0.4	4.6	142	12.2	154.2
SL	Sierra Leone 2008	1	6.3	1.5	4.9	10.2	8.2	5.1	0.4	5.5	180	12.1	192.1
SL	Sierra Leone 2013	1	6.8	1.6	5.2	22.1	20.9	4.9	0.4	5.3	169	12.3	181.3
SN	Senegal 2012	1	9.3	2.5	6.8	12.6	11.4	5.3	0.6	5.9	172	17.6	189.6
SN	Senegal 2014	1	8.3	2.1	6.2	16.0	14.7	5.0	0.5	5.5	167	15.2	182.2
SN	Senegal 2015	1	9.1	2.4	6.7	16.9	15.3	4.9	0.6	5.5	161	16.1	177.1
SN	Senegal 2016	1	9.1	2.4	6.7	18.0	16.6	4.7	0.5	5.2	156	15.6	171.6
SN	Senegal 2017	1	10.3	2.9	7.4	19.9	18.9	4.6	0.6	5.2	152	17.5	169.5
TZ	Tanzania 2004	1	8.8	2.3	6.5	22.5	17.6	5.7	0.6	6.3	199	19.2	218.2
TZ	Tanzania 2010	1	8.1	2	6.0	28.8	23.6	5.4	0.5	5.9	188	16.5	204.5
TZ	Tanzania 2015	1	9.8	2.7	7.1	32.4	27.1	5.2	0.6	5.8	178	19.3	197.3
UG	Uganda 2006	1	9.7	2.7	7.1	19.6	15.4	6.7	0.8	7.5	230	24.7	254.7
UG	Uganda 2011	1	10.0	2.8	7.2	23.6	20.7	6.2	0.8	7.0	217	24.1	241.1
UG	Uganda 2016	2	10.9	3.2	7.8	30.3	27.3	5.4	0.7	6.1	189	23.2	212.2
ZM	Zambia 2007	1	6.2	1.4	4.8	29.9	24.6	6.2	0.4	6.6	214	14.2	228.2
ZM	Zambia 2013	1	5.6	1.3	4.4	35.1	32.5	5.3	0.3	5.6	184	11.0	195.0
ZW	Zimbabwe 1994	1	8.2	2.1	6.1	35.1	31.1	4.3	0.4	4.7	148	13.3	161.3
ZW	Zimbabwe 1999	1	8.2	2.1	6.1	37.7	35.6	4.0	0.4	4.4	141	12.5	153.5
ZW	Zimbabwe 2005	1	7.3	1.8	5.5	40.1	39.1	3.8	0.3	4.1	137	10.7	147.7
ZW	Zimbabwe 2010	1	7.0	1.7	5.3	41.3	40.5	4.1	0.3	4.4	150	11.3	161.3
ZW	Zimbabwe 2015	1	8.5	2.2	6.3	48.6	47.9	4.0	0.4	4.4	144	13.3	157.3
**Central and West Asia & Europe**													
AL	Albania 2008	2	16.0	**7.2**	8.7	48.0	7.9	1.6	0.3	1.9	46	8.7	54.7
AL	Albania 2017	1	9.2	2.4	6.7	33.2	2.8	1.8	0.2	2.0	57	5.8	62.8
AM	Armenia 2000	4	62.8	58.5	4.4	39.0	14.4	1.7	3.1	4.8	56	94.7	150.7

Code	Survey	Cluster	Probability PT (%)			Contraception (%)		Total rates			General rates		
				Model estimates									
			T	IA	ST	Any	Modern	TFR	TTR	TPR	GFR	GTR	GPR
AM	Armenia 2005	4	51.9	**44.8**	7.2	33.1	12.3	1.7	2.1	3.8	58	62.7	120.7
AM	Armenia 2010	3	36.6	**29.6**	7.0	33.9	16.9	1.7	1.1	2.8	61	35.2	96.2
AM	Armenia 2015	3	32.4	**22.2**	10.2	36.7	18.1	1.7	0.9	2.6	64	30.6	94.6
AZ	Azerbaijan 2006	4	52.2	**45.7**	6.5	32.0	9.0	2.0	2.4	4.4	66	72.1	138.1
KK	Kazakhstan 1999	4	46.9	38.6	8.4	48.0	38.7	2.0	1.8	3.8	67	59.3	126.3
KY	Kyrgyz Rep. 2012	3	22.4	**12.3**	10.1	24.4	22.7	3.6	1.1	4.7	125	36.2	161.2
MB	Moldova 2005	4	44.1	**32.6**	11.5	49.8	32.8	1.7	1.4	3.1	55	43.4	98.4
TJ	Tajikistan 2012	3	16.0	**7.8**	8.2	18.9	17.5	3.8	0.8	4.6	134	25.4	159.4
TJ	Tajikistan 2017	2	15.9	**8.2**	7.7	21.3	19.7	3.8	0.8	4.6	141	26.6	167.6
TR	Turkey 1998	3	24.5	12.4	12.1	44.2	26.1	2.6	1.0	3.6	94	30.6	124.6
TR	Turkey 2003	3	23.0	**11.2**	11.8	71.0	42.5	2.2	0.7	2.9	79	23.6	102.6
UA	Ukraine 2007	3	34.0	**25.9**	8.1	50.9	38.3	1.2	0.6	1.8	39	20.1	59.1
**Latin America**													
BO	Bolivia 1994	1	9.0	2.4	6.6	30.1	11.9	4.8	0.5	5.3	163	16.2	179.2
BO	Bolivia 2008	1	12.9	4.1	8.8	41.3	24.0	3.5	0.6	4.0	121	18.0	139.0
BR	Brazil 1996	2	13.5	4.4	9.1	55.4	51.0	2.5	0.4	2.9	89	13.9	102.9
CO	Colombia 1990	2	12.5	3.9	8.6	39.9	33.0	2.8	0.4	3.2	105	15.0	120.0
CO	Colombia 1995	1	11.3	3.3	8.0	48.1	39.5	3.0	0.4	3.4	107	13.6	120.6
CO	Colombia 2000	2	15.7	5.7	10.1	52.8	43.8	2.6	0.5	3.1	92	17.2	109.2
CO	Colombia 2005	2	17.8	7	10.8	56.4	49.4	2.4	0.5	2.9	84	18.2	102.2
CO	Colombia 2010	2	17.8	7	10.8	61.2	56.9	2.1	0.5	2.6	74	16.0	90.0
CO	Colombia 2015	2	15.4	**3**	12.4	64.9	61.4	2.0	0.4	2.4	70	12.8	82.8
DR	Dominican Rep. 1991	2	14.4	4.9	9.5	36.8	33.9	3.3	0.6	3.9	125	21.0	146.0
DR	Dominican Rep. 1996	2	16.8	6.3	10.5	44.6	41.3	3.2	0.7	3.9	120	24.2	144.2
DR	Dominican Rep. 1999	2	21.8	10	11.8	48.8	45.6	2.7	0.8	3.5	100	27.9	127.9
DR	Dominican Rep. 2002	2	16.2	5.9	10.2	51.2	48.2	3.0	0.6	3.6	110	21.2	131.2
GU	Guatemala 1995	1	6.0	1.3	4.6	21.4	18.4	5.1	0.4	5.4	177	11.2	188.2
GU	Guatemala 1998	1	5.8	1.3	4.5	26.6	21.7	5.0	0.3	5.3	177	11.0	188.0
GU	Guatemala 2014	1	7.8	2	5.9	39.4	32.2	3.1	0.3	3.4	112	9.5	121.5

			Probability PT (%)										
				Model estimates		Contraception (%)		Total rates			General rates		
Code	Survey	Cluster	T	IA	ST	Any	Modern	TFR	TTR	TPR	GFR	GTR	GPR
GY	Guyana 2009	2	21.8	10	11.8	34.6	32.5	2.8	0.8	3.6	94	26.3	120.3
HN	Honduras 2005	1	9.1	2.4	6.7	43.2	37.7	3.3	0.4	3.7	117	11.7	128.7
HN	Honduras 2011	1	9.8	2.7	7.1	48.8	42.9	2.9	0.3	3.2	107	11.7	118.7
NC	Nicaragua 1998	1	8.0	2	6.0	40.8	39.0	3.6	0.3	3.9	132	11.5	143.5
PE	Peru 1991	1	10.2	2.9	7.4	35.7	19.9	3.5	0.4	4.0	121	13.8	134.8
PE	Peru 1996	1	10.0	2.8	7.3	40.9	26.4	3.5	0.4	3.9	122	13.6	135.6
PE	Peru 2000	1	10.3	2.9	7.4	44.0	32.0	2.8	0.3	3.1	98	11.2	109.2
PE	Peru 2004	1	11.3	3.3	8.0	45.8	30.9	2.6	0.3	2.9	87	11.1	98.1
PE	Peru 2007	2	14.0	4.7	9.3	48.0	33.0	2.5	0.4	2.9	85	13.8	98.8
PE	Peru 2009	2	14.0	4.7	9.3	49.2	34.2	2.6	0.4	3.0	88	14.4	102.4
PE	Peru 2010	2	15.8	5.7	10.1	50.1	34.7	2.5	0.5	3.0	86	16.1	102.1
PE	Peru 2011	2	15.1	5.3	9.8	50.9	35.3	2.6	0.5	3.1	87	15.5	102.5
PY	Paraguay 1990	1	10.9	3.2	7.8	32.7	23.6	4.7	0.6	5.3	160	19.6	179.6
**South and Southeast Asia**													
IA	India 2005	2	12.2	3.7	8.4	43.8	38.0	2.7	0.4	3.1	101	14.0	115.0
ID	Indonesia 2012	1	10.6	**0.2**	10.5	45.7	42.7	2.6	0.3	2.9	88	10.4	98.4
KH	Cambodia 2010	3	21.6	9.8	11.8	31.4	21.7	3.0	0.9	3.9	105	28.9	133.9
KH	Cambodia 2014	3	23.9	11.8	12.1	38.5	26.6	2.7	0.9	3.6	98	30.8	128.8
NP	Nepal 2011	2	14.9	**7.1**	7.8	38.2	33.2	2.6	0.5	3.1	96	16.8	112.8
NP	Nepal 2016	3	19.8	**8.9**	10.8	40.8	33.2	2.3	0.6	2.9	88	21.7	109.7
PH	Philippines 1993	1	9.7	2.6	7.0	24.2	15.1	4.1	0.5	4.6	138	14.8	152.8
PH	Philippines 1998	1	10.8	3.1	7.7	28.9	17.2	3.7	0.5	4.2	126	15.2	141.2
PH	Philippines 2003	1	10.4	**0.6**	9.8	31.6	21.6	3.5	0.4	3.9	119	13.8	132.8
TL	Timor Leste 2009	1	2.9	0.6	2.4	13.6	12.8	5.7	0.2	5.9	175	5.3	180.3
TL	Timor Leste 2016	1	3.4	0.7	2.7	16.1	14.8	4.2	0.2	4.4	136	4.8	140.8

Note:
Values in boldface correspond to induced abortion estimates from reported data.
